# Borane-catalyzed cascade Friedel–Crafts alkylation/[1,5]-hydride transfer/Mannich cyclization to afford tetrahydroquinolines†

**DOI:** 10.1039/d1sc05629h

**Published:** 2021-12-20

**Authors:** Bei-Bei Zhang, Shuo Peng, Feiyi Wang, Cuifen Lu, Junqi Nie, Zuxing Chen, Guichun Yang, Chao Ma

**Affiliations:** Hubei Collaborative Innovation Centre for Advanced Organic Chemical Materials, College of Chemistry and Chemical Engineering, Ministry of Education Key Laboratory for the Synthesis and Application of Organic Functional Molecules, Hubei University Wuhan 430062 P. R. China machao@hubu.edu.cn yangguichun@hubu.edu.cn

## Abstract

An unprecedented redox-neutral annulation reaction of tertiary anilines with electron-deficient alkynes was developed that proceeds through a cascade Friedel–Crafts alkylation/[1,5]-hydride transfer/Mannich cyclization sequence. Under B(C_6_F_5_)_3_ catalysis, a range of functionalized 1,2,3,4-tetrahydroquinolines were facilely constructed in moderate to good yields with exclusive 3,4-*anti*-stereochemistry. The commercial availability of the catalyst and the high atom and step economy of the procedure, together with metal-free and external oxidant-free conditions, make this an attractive method in organic synthesis.

## Abstract 2

We report a redox-neutral annulation reaction of tertiary amines with electron-deficient alkynes under metal-free and oxidant-free conditions.

## Graphical abstract

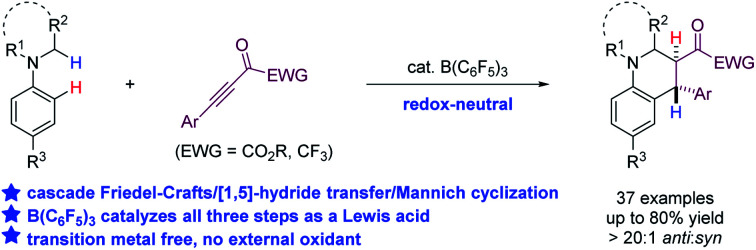

## Introduction

**Scheme 1:**
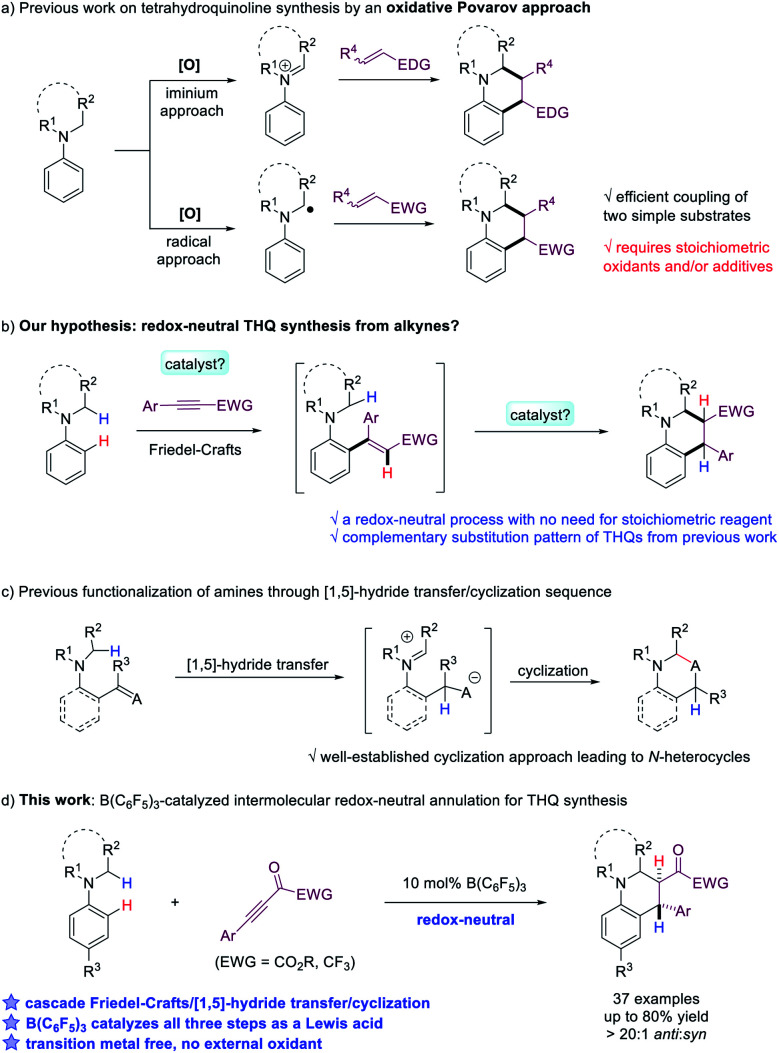
Different catalytic strategies for THQ synthesis.

Tetrahydroquinoline (THQ) is one of the most valuable N-heterocyclic scaffolds that exists in a large number of natural products and bioactive molecules.^1^ Due to its importance, development of efficient methods to construct the THQ skeleton has been extensively explored in organic synthesis. Various approaches have been established, including intramolecular cyclization,^2^ Povarov reactions,^3^ hydrogenation of quinolines^4^ and others.^5^ Among all the strategies, the oxidative Povarov reaction, a dehydrogenative [4 + 2] annulation reaction between *N*-alkylanilines and alkenes, represents a promising protocol for the construction of THQs from simple starting materials (Scheme 1a).^6^ In this reaction, two new C–C bonds were formed *via* a cascade sp^3^ and sp^2^ C–H functionalization process. However, such a process requires stoichiometric oxidants to convert *N*-alkylanilines to iminium ions or α-aminoalkyl radical intermediates, which then undergo reaction with activated olefins. The development of redox-neutral [4 + 2] annulation reaction for the synthesis of THQs, which avoids the use of external oxidants, will be highly desirable. We hypothesize that the combination of *N*-alkylanilines and electron-deficient alkynes represents a promising approach (Scheme 1b). In addition to the attractive feature of high atom-economy, it is worth noting that THQs produced in this fashion also possess complementary substitution patterns compared to previous work. However, challenges need to be addressed to identify an effective catalytic system that promotes an efficient cascade Friedel–Crafts type addition to the alkyne followed by cyclization.

For the proposed cyclization step, it is noteworthy that a [1,5]-hydride transfer/cyclization process has been developed into a powerful and versatile protocol for the synthesis of structurally diverse O- or N-heterocycles including THQs in recent years (Scheme 1c).^7^ In such processes, *ortho*-substituted functionalities such as aldehydes, ketones, imines and electron-deficient alkenes serve as hydride acceptors to construct N-heterocycles with high efficiency and fidelity.^8^ However, it is necessary to construct *ortho*-disubstituted substrates for intramolecular cyclization to proceed in literature precedents. If the substrate synthesis from readily available building blocks can be coupled with the cyclization into a cascade process, it should lead to a highly attractive and flexible synthesis of valuable heterocycles.

The last decade has witnessed an explosive development of tris(pentafluorophenyl)borane-catalyzed reactions.^9^ In addition, B(C_6_F_5_)_3_-promoted hydride transfer of amines and *N*-alkylanilines^10^ has also resulted in various step- and atom-economical transformations of these valuable substrates.^11–14^ In particular, B(C_6_F_5_)_3_-catalyzed intramolecular cyclization of *ortho*-substituted *N*,*N*-dialkyl arylamines *via* hydride transfer to afford tetrahydroquinoline derivatives was realized by the Paradies group and Wang group, respectively.^15,16^ Nevertheless, the synthesis of tetrahydroquinolines through B(C_6_F_5_)_3_-catalyzed intermolecular redox-neutral annulation still remains elusive in the literature.

Herein, we report an unprecedented intermolecular redox-neutral annulation reaction of *N*-alkylanilines with electron-deficient alkynes catalyzed by B(C_6_F_5_)_3_ to deliver a range of poly-substituted tetrahydroquinolines in good to high yields (Scheme 1d). Our mechanistic studies indicate that this efficient and flexible [4 + 2] annulation reaction involves a cascade Friedel–Crafts alkylation/[1,5]-hydride transfer/Mannich cyclization sequence. In all the steps, B(C_6_F_5_)_3_ serves as an effective Lewis acid catalyst with the cooperative effect from TMSOTf.

## Results and discussion

At the outset, we intend to study the annulation reaction of *N*-alkylanilines with alkynes by using B(C_6_F_5_)_3_ as the catalyst. To our delight, the reaction between *N*,*N*-4-trimethylaniline 1a and β,γ-alkynyl-α-ketoester 2a occurred smoothly with 10 mol% B(C_6_F_5_)_3_ in toluene at 80 °C. The desired 3,4-*anti*-tetrahydroquinoline 3a was obtained in 46% yield and > 20 : 1 dr with concomitant formation of the conjugate addition product 4a (Table 1, entry 1). Several other common Lewis acids such as Cu(OTf)_2_, Mg(OTf)_2_, In(OTf)_3_ and BF_3_·OEt_2_ turned out to be ineffective for the reaction (entries 2–5). Gratifyingly, the use of a catalytic amount of TMSOTf as an additive, which has been proved to improve the reaction efficiency by the Wang group^15*b*^ and the Oestreich group,^12*g*^ was beneficial for the [4 + 2] annulation reaction, affording the desired product 3a in 69% yield, while no byproduct 4a was observed (entry 6). Next, the solvent effect was evaluated. While dioxane, *m*-xylene and DCM gave slightly lower or similar yields (entries 7–9), no product or significantly decreased yield was obtained by the use of MeCN or THF as the solvent (entries 10–11). No improvement was obtained by increasing the amount of TMSOTf (entry 12). It is noteworthy that no product could be detected when TMSOTf itself was employed as the catalyst (entry 13).

**Table 1:** Optimization of the reaction conditions^a^

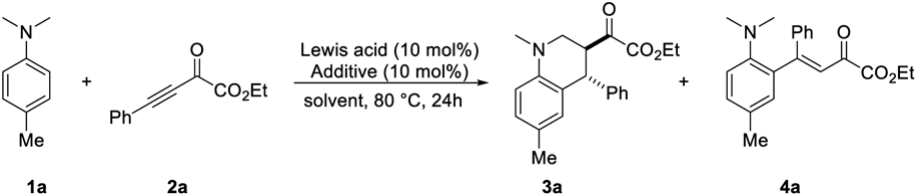					
Entry	Lewis acid	Additive	Solvent	Yield^b^ of 3a (%)	Yield^b^ of 4a (%)
1	B(C_6_F_5_)_3_	None	Toluene	46	27
2	Cu(OTf)_2_	None	Toluene	<2	<2
3	Mg(OTf)_2_	None	Toluene	<2	<2
4	In(OTf)_3_	None	Toluene	<2	<2
5	BF_3_·OEt_2_	None	Toluene	<2	<2
**6**	**B(C**_**6**_ **F**_**5**_ **)**_**3**_	**TMSOTf**	**Toluene**	**69**	**<2**
7	B(C_6_F_5_)_3_	TMSOTf	Dioxane	58	19
8	B(C_6_F_5_)_3_	TMSOTf	*m*-Xylene	63	<5
9	B(C_6_F_5_)_3_	TMSOTf	DCM	69	<2
10	B(C_6_F_5_)_3_	TMSOTf	MeCN	<2	<2
11	B(C_6_F_5_)_3_	TMSOTf	THF	<5	10
12^c^	B(C_6_F_5_)_3_	TMSOTf	Toluene	68	<2
13	None	TMSOTf	Toluene	<2	<2

a Unless otherwise noted, the reactions were carried out by stirring 1a (0.24 mmol), 2a (0.2 mmol), B(C_6_F_5_)_3_ (0.02 mmol) and additive (0.02 mmol) in 1 mL of solvent at 80 °C for 24 hours under N_2_.

b Isolated yield, >20 : 1 dr of 3a was determined by ^1^H NMR.

c With 20 mol% TMSOTf.

**Scheme 2:**
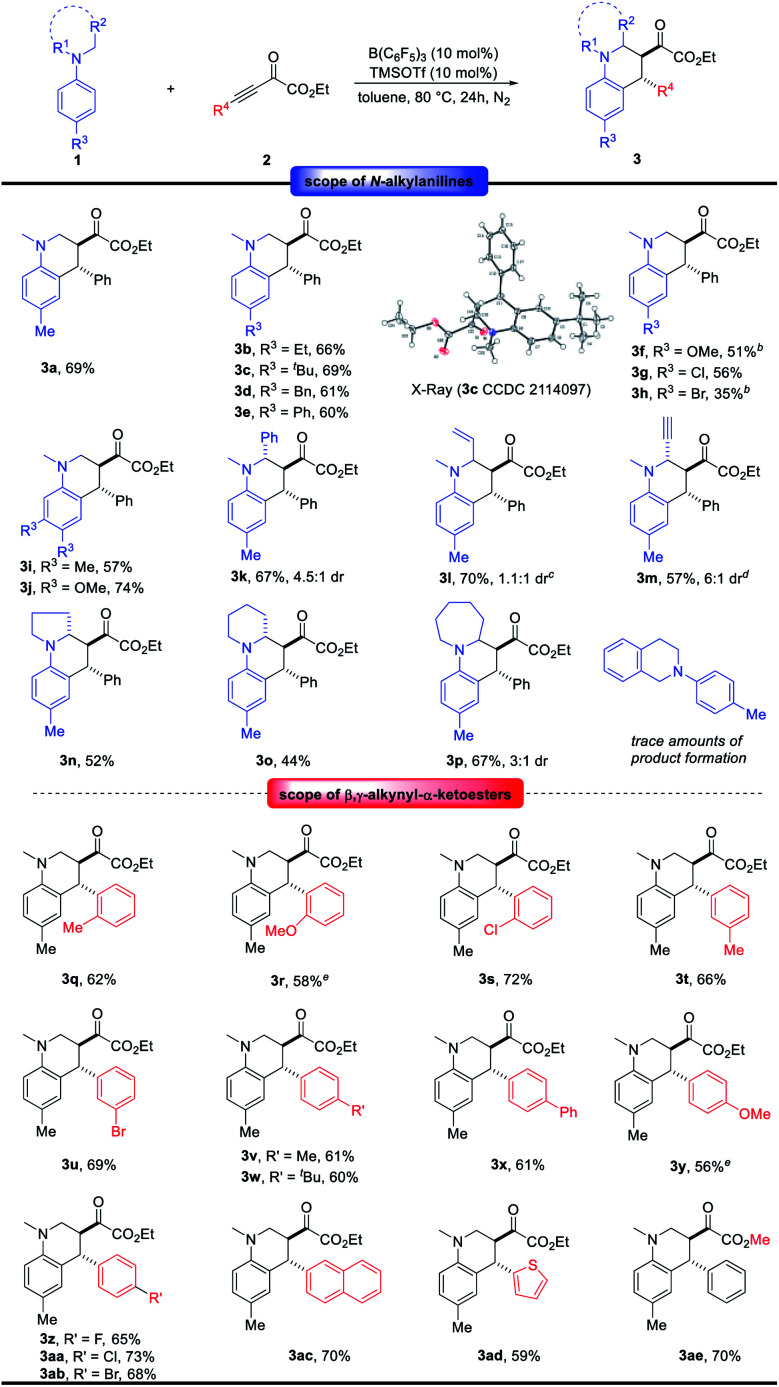
Scope of redox-neutral annulation between *N*-alkylanilines and β,γ-alkynyl-α-ketoesters. ^*a*^ See Table 1 and ESI† for a detailed procedure, unless otherwise noted, the dr of 3 was >20 : 1. ^*b*^ The reaction was performed with 10 mol% B(C_6_F_5_)_3_ and 20 mol% TMSOTf for 48 hours. ^*c*^ The reaction was performed at 25 °C for 48 hours. ^*d*^ The reaction was performed with 15 mol% B(C_6_F_5_)_3_ and 10 mol% TMSOTf at 25 °C for 72 hours. ^*e*^ The reaction was performed for 36 hours.

With the optimal conditions in hand (Table 1, entry 6), the generality of this B(C_6_F_5_)_3_-catalyzed redox-neutral annulation reaction between *N*-alkylanilines 1 and electron-deficient alkynes 2 was investigated (Scheme 2). Various *para*-substituted *N*,*N*-dimethylanilines 1 were compatible in this reaction and delivered the corresponding products 3a–3g in 51–69% yields. The structure of 3c has been unambiguously confirmed by X-ray crystallographic analysis. A lower yield was obtained for 4-bromo-*N*,*N*-dimethylaniline. Disubstituted *N*,*N*-dimethylanilines underwent conjugate addition favorably at the sterically less demanding position, furnishing products 3i and 3j in 57 and 74% yields, respectively. When one of the methyl groups on the amino moiety was replaced by the benzyl group, the reaction proceeded chemoselectively at the benzyl group to form 3k in 67% yield with 4.5 : 1 dr, which indicates the superior hydride donor capability of benzylic over primary C–H bonds.^8*d*,*i*,*k*,*t*^ The structure of 3k has been unambiguously confirmed by X-ray crystallographic analysis (see ESI†). Additionally, different functional groups on the amino moiety including alkene and alkyne were well tolerated under this catalytic system at ambient reaction temperature. The desired products 3l and 3m were obtained in moderate yields. Cyclic amine derivatives showed good reactivity in this reaction, and products 3n–3p were obtained in 44–67% yields. When studying further *N*,*N*-dialkyl arylamines, we found that *N*-aryl tetrahydro-isoquinoline was not compatible with the reaction conditions, and only trace amounts of product were observed. Next, the variation of β,γ-alkynyl-α-ketoesters 2 was studied. Substrates 2 containing electron-neutral, electron-donating and electron-withdrawing groups on the *ortho*-, *meta*- and *para*-positions on the aryl ring were well tolerated under the standard reaction conditions, giving products 3q–3ab in 56–73% yields and high *anti* diastereoselectivity. In addition, 2-naphthyl and 2-thienyl substituents have been tested, providing products 3ac and 3ad in 70 and 59% yields, respectively. The reaction of methyl ester with 1a led to the desired product 3ae in 70% yield.

**Scheme 3:**
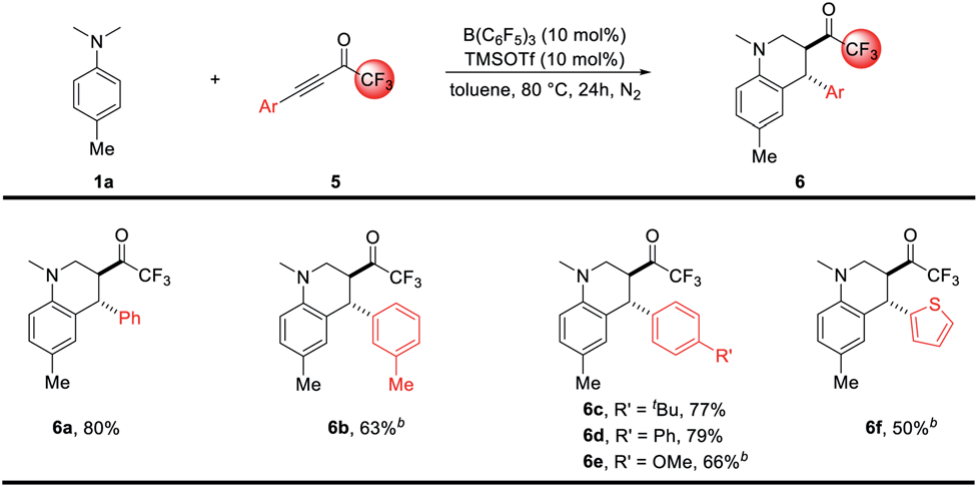
Scope of redox-neutral annulation between *N*-alkylanilines and trifluoromethyl-α,β-ynones. ^*a*^ See Table 1 and ESI† for a detailed procedure, the dr of 6 was >20 : 1. ^*b*^ The reaction was performed with 10 mol% B(C_6_F_5_)_3_ and 20 mol% TMSOTf for 52 hours.

The redox-neutral annulation between *N*,*N*-4-trimethyl-aniline 1a and trifluoromethyl-α,β-ynones 5 was also investigated. As exemplified in Scheme 3, the reactions went smoothly under the current B(C_6_F_5_)_3_ catalytic system. An array of trifluoromethylated tetrahydroquinolines 6a–6f were prepared in moderate to good yields with high diastereoselectivity (>20 : 1, *anti* : *syn*).

**Scheme 4:**
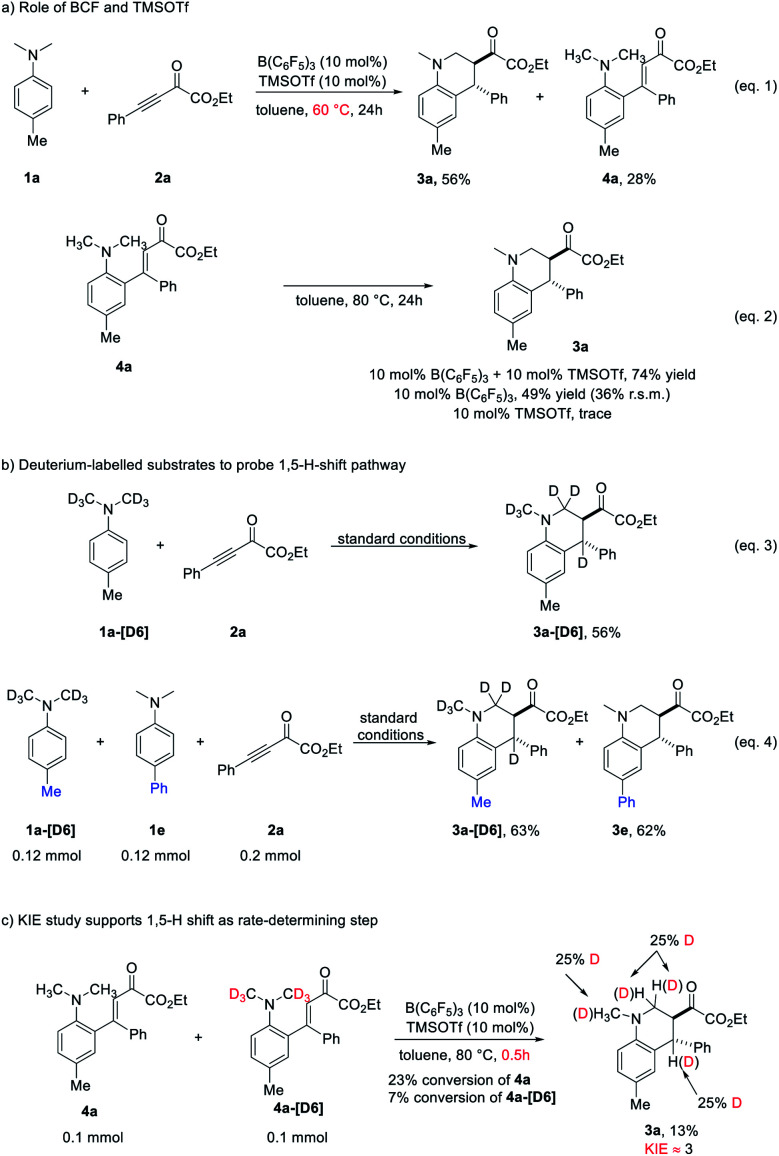
Control experiments.

To shed light on the mechanism of the redox-neutral annulation, a series of control experiments were performed (Scheme 4). When the reaction between 1a and 2a was performed at a slightly lower temperature (60 °C) for 24 hours, the starting material 2a was consumed and the desired product 3a was obtained in diminished yield with concomitant formation of 4a (Scheme 4a, eqn (1)). Subsequently, annulation of 4a could occur under the standard conditions to generate 3a in 74% yield (Scheme 4a, eqn (2)). These results indicated that a cascade Friedel–Crafts alkylation/intramolecular hydride transfer/Mannich cyclization sequence was included in the redox-neutral [4 + 2] annulation reaction. The hydride transfer is probably the rate-determining step. We performed the intramolecular cyclization of 4a without the addition of TMSOTf or B(C_6_F_5_)_3_ (Scheme 4a, eqn (2)). The reactivity of intramolecular cyclization could be enhanced in the presence of TMSOTf. However, probably due to the relatively weak Lewis acidity, TMSOTf itself was unable to promote the reaction. Next, the reaction of isotope-labelled 1a-[D6] with 2a under standard conditions was performed, giving 3a-[D6] in 56% yield with deuterium exclusively transferred to the benzylic position (Scheme 4b, eqn (3)). Besides, the reaction of 2a with a mixture of 1a-[D6] and 1e under standard conditions provided a mixture of 3a-[D6] and 3e without exchange of H/D (Scheme 4b, eqn (4)). These observations suggested that a [1,5]-hydride shift process was involved in the reaction and excluded the hydride abstraction mediated by the borane.^15^ A crossover experiment using 4a and 4a-[D6] was performed then in a shortened reaction time (Scheme 4c). At low conversions, a significant kinetic isotope effect (KIE) of 3.0 was observed, revealing that the [1,5]-hydride transfer could be involved in the rate-determining step.

**Scheme 5:**
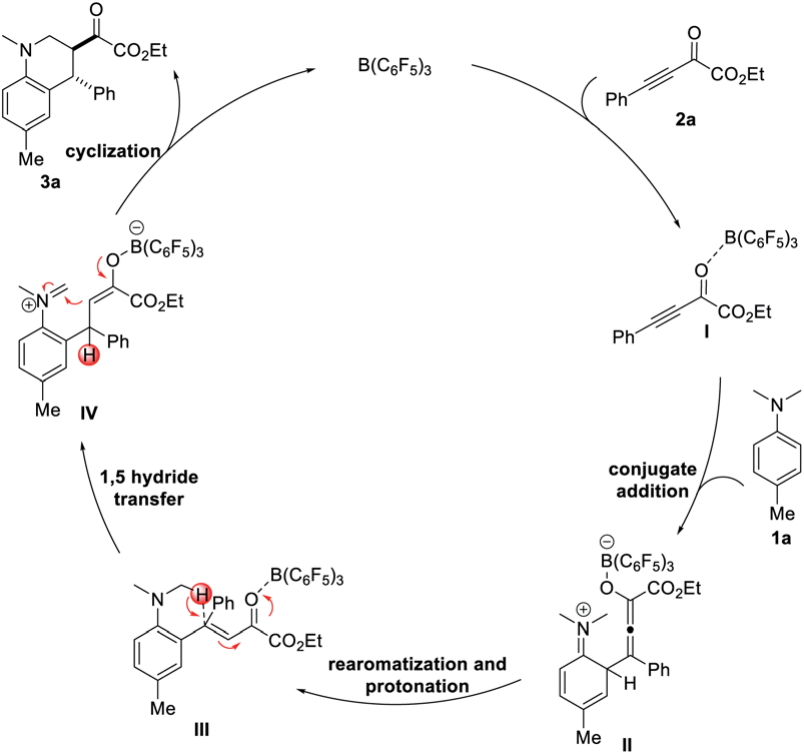
Proposed catalytic cycle.

Based on the experimental results and previous report,^15*c*^ a plausible mechanism for the B(C_6_F_5_)_3_-catalyzed redox-neutral [4 + 2] annulation of *N*-alkylanilines and electron-deficient alkynes is proposed in Scheme 5. The reaction is initiated by the activation of β,γ-alkynyl-α-ketoester 2a with B(C_6_F_5_)_3_, which results in the formation of electrophilic alkyne I. *N*,*N*-4-trimethylaniline 1a reacts with intermediate I to form allenolate intermediate II. Subsequent rearomatization and protonation of the allenolate generate intermediate III. 1,5-Hydride transfer then occurs with the assistance of B(C_6_F_5_)_3_ to afford iminium enolate IV. Final intramolecular Mannich cyclization produced tetrahydroquinoline product 3a with the regeneration of B(C_6_F_5_)_3_.

## Conclusions

In summary, we have developed a B(C_6_F_5_)_3_-catalyzed cascade reaction of tertiary anilines with electron-deficient alkynes to construct poly-substituted tetrahydroquinoline derivatives with excellent step and atom economy. This redox neutral reaction involves a sequential Friedel–Crafts alkylation/[1,5]-hydride transfer/Mannich cyclization sequence without using a transition metal or an external oxidant. An array of 1,2,3,4-tetrahydroquinolines were accessed in high efficiency and diastereoselectivity.

## Data availability

All experimental and characterization data in this article are available in the ESI.† Crystallographic data for compounds 3c and 3k have been deposited in the Cambridge Crystallographic Data Centre (CCDC) under accession numbers CCDC 2114097 and 2114110, respectively.†

## Author contributions

B.-B. Z. performed the synthetic experiments and analysed the data, with help from S. P., F. W., C. L., J. N. and Z. C.; C. M. and G. Y. directed the project and wrote the manuscript. All authors discussed the results and commented on the manuscript.

## Conflicts of interest

There are no conflicts to declare.

## Supplementary Material

SC-013-D1SC05629H-s001

SC-013-D1SC05629H-s002

## References

[cit1] Sridharan V., Suryavanshi P. A., Menéndez J. C. (2011). Chem. Rev..

[cit2] Kretzschmar M., Hofmann F., Moock D., Schneider C. (2018). Angew. Chem., Int. Ed..

[cit3] Bush T. S., Yap G. P. A., Chain W. J. (2018). Org. Lett..

[cit4] Sorribes I., Liu L., Doménech-Carbó A., Corma A. (2018). ACS Catal..

[cit5] Zhang M.-M., Wang Y.-N., Wang B.-C., Chen X.-W., Lu L.-Q., Xiao W.-J. (2019). Nat. Commun..

[cit6] Murata S., Miura M., Nomura M. (1989). J. Org. Chem..

[cit7] Peng B., Maulide N. (2013). Chem.–Eur. J..

[cit8] Verboom W. D., Reinhoudt N., Visser R., Harkema S. (1984). J. Org. Chem..

[cit9] Melen R. L. (2014). Chem. Commun..

[cit10] Millot N., Santini C. C., Fenet B., Basset J. M. (2002). Eur. J. Inorg. Chem..

[cit11] Shang M., Chan J. Z., Cao M., Chang Y., Wang Q., Cook B., Torker S., Wasa M. (2018). J. Am. Chem. Soc..

[cit12] Zhang J., Park S., Chang S. (2018). J. Am. Chem. Soc..

[cit13] Maier A. F. G., Tussing S., Schneider T., Flörke U., Qu Z.-W., Grimme S., Paradies J. (2016). Angew. Chem., Int. Ed..

[cit14] Farrell J. M., Heiden Z. M., Stephan D. W. (2011). Organometallics.

[cit15] Maier A. F. G., Tussing S., Zhu H., Wicker G., Tzvetkova P., Flörke U., Daniliuc C. G., Grimme S., Paradies J. (2018). Chem.–Eur. J..

[cit16] Gandhamsetty N., Joung S., Park S.-W., Park S., Chang S. (2014). J. Am. Chem. Soc..

