# Supplementary material for: Borane-catalyzed cascade Friedel–Crafts alkylation/[1,5]-hydride transfer/Mannich cyclization to afford tetrahydroquinolines
Source: Chem Sci. 2021 Dec 20;13(3):775–80. doi: 10.1039/d1sc05629h (PMC8768868; doi:10.1039/d1sc05629h)

Electronic Supplementary Material (ESI) for Chemical Science.
This journal is © The Royal Society of Chemistry 2021

Borane-Catalyzed Cascade Friedel-Crafts Alkylation/[1,5]-Hydride Transfer/Mannich Cyclization to Afford Tetrahydroquinolines

Bei-Bei Zhang, Shuo Peng, Feiyi Wang, Cuifen Lu, Junqi Nie, Zuxing Chen, Guichun Yang*, and Chao Ma*,

Hubei Collaborative Innovation Center for Advanced Organic Chemical Materials, College of Chemistry and Chemical Engineering, Ministry of Education Key Laboratory for the Synthesis and Application of Organic Functional Molecules, Hubei University, Wuhan 430062, P.R. China.

Email: machao@hubu.edu.cn; yangguichun@hubu.edu.cn

Contents:

1. General information S2

2. General procedure for syntheses of functionalized 1,2,3,4-tetrahydroquinolines S3

3. Analytical data for products S4

4. Mechanistic studies S25

5. X-Ray crystallography data S30

6. References S33

7. NMR spectra of products S34

1. General information

^{1}H and **^{13}C NMR** spectra were recorded on a Bruker (400 MHz) spectrometer. Chemical shifts were reported in parts per million (ppm), and the residual solvent peak was used as an internal reference: proton (chloroform δ 7.26), carbon (chloroform δ 77.0) or tetramethylsilane (TMS δ 0.00) was used as a reference. Multiplicity was indicated as follows: s (singlet), d (doublet), t (triplet), q (quartet), m (multiplet), dd (doublet of doublet), bs (broad singlet). Coupling constants were reported in Hertz (Hz). All high resolution mass spectra (**HRMS**) were obtained on Agilent 1260-6224 LC-MS TOF using ESI (electrospray ionization). For thin layer chromatography (**TLC**), Merck pre-coated TLC plates (Merck 60 F254) were used, and compounds were visualized with a UV light at 254 nm. Further visualization was achieved by staining with I_2 and $KMnO_4$.

All reactions were carried out under nitrogen atmosphere. All commercially available reagents were used as received for the reactions without any purification. All solvents were dried on alumina columns using a solvent dispensing system. $B(C_6F_5)_3$ were purchased from TCI. Tertiary anilines **1** were synthesized following the reported procedure[1]. Alkynones **2** and **5** were synthesized following the reported procedure[2,3].

2. General procedure for syntheses of functionalized 1,2,3,4-tetrahydroquinolines

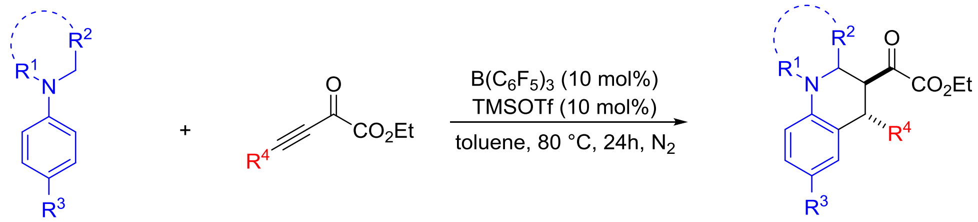

1 **2** **3**

To a Schlenk tube equipped with a dried stir bar was added $B(C_6F_5)_3$ (0.02 mmol), tertiary aniline **1** (0.24 mmol), alkynone **2** (0.20 mmol), TMSOTf (0.02 mmol) and toluene (1.0 mL) in the glovebox. The Schlenk tube was sealed with a Teflon screw cap. The reaction mixture was taken outside the glovebox and allowed to stir at 80 ℃ for 24 hours. The crude reaction mixture was concentrated under reduced pressure and directly purified by silica gel chromatography (ethyl acetate:hexanes = 1:10) to afford the desired functionalized 1,2,3,4-tetrahydroquinolines **3**.

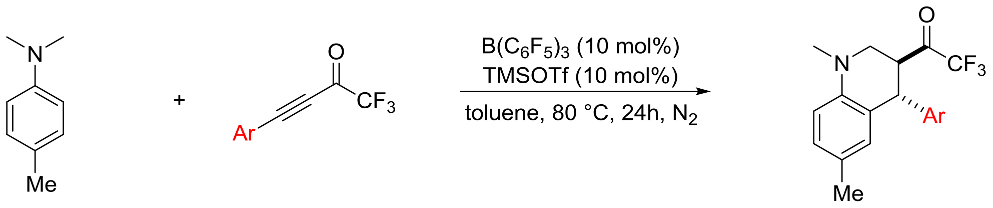

1a **5** **6**

To a Schlenk tube equipped with a dried stir bar was added $B(C_6F_5)_3$ (0.02 mmol), *N*,*N*,4-trimethylaniline **1a** (0.24 mmol), trifluoromethyl-α,β-ynones **5** (0.20 mmol), TMSOTf (0.02 mmol) and toluene (1.0 mL) in the glovebox. The Schlenk tube was sealed with a Teflon screw cap. The reaction mixture was taken outside the glovebox and allowed to stir at 80 ℃ for 24 hours. The crude reaction mixture was concentrated under reduced pressure and directly purified by silica gel chromatography (ethyl acetate:hexanes = 1:10) to afford the desired functionalized tetrahydroquinolines **6**.

3. Analytical data for products

ethyl 2-(*trans*-1,6-dimethyl-4-phenyl-1,2,3,4-tetrahydroquinolin-3-yl)-2-oxo-acetate (3a):

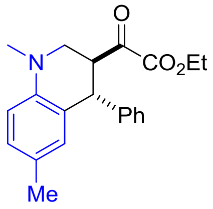

3a

Flash column chromatography (eluent: EtOAc/Hexanes = 1/10, v/v) to afford **3a** as a yellow oil, 46.5 mg, 69% yield, >20:1 dr. Rf = 0.47 (1:5 EtOAc/Hexanes).

^{1}H NMR (400 MHz, Chloroform-*d*) δ 7.31 – 7.26 (m, 2H), 7.24 – 7.20 (m, 1H), 7.14 – 7.12 (m, 2H), 6.95 (dd, *J* = 8.3, 2.2 Hz, 1H), 6.64 – 6.61 (m, 2H), 4.50 (d, *J* = 6.5 Hz, 1H), 4.25 – 4.17 (m, 2H), 3.80 (td, *J* = 6.7, 3.4 Hz, 1H), 3.42 (dd, *J* = 11.3, 6.4 Hz, 1H), 3.33 (dd, *J* = 11.7, 3.4 Hz, 1H), 2.93 (s, 3H), 2.13 (s, 3H), 1.28 (t, *J* = 7.1 Hz, 3H).

^{13}C NMR (101 MHz, Chloroform-*d*) δ 194.66, 161.26, 144.53, 143.93, 130.72, 129.11, 128.44, 128.28, 126.68, 123.29, 111.91, 62.30, 49.76, 44.59, 39.68, 20.24, 13.89.

HRMS (ESI): m/z Calcd. for $[C_{21}H_{24}NO_3, M+H]^+$: 338.1750; Found: 338.1750.

ethyl 2-(*trans*-6-ethyl-1-methyl-4-phenyl-1,2,3,4-tetrahydroquinolin-3-yl)-2-oxoacetate (3b):

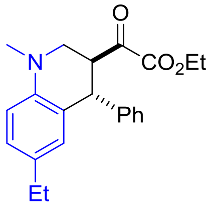

3b

Flash column chromatography (eluent: EtOAc/Hexanes = 1/10, v/v) to afford **3b** as a yellow solid, 46.3 mg, 66% yield, >20:1 dr. m.p.: 78~79 ℃. Rf = 0.47 (1:5 EtOAc/Hexanes).

^{1}H NMR (400 MHz, Chloroform-*d*) δ 7.31 – 7.26 (m, 2H), 7.24 – 7.20 (m, 1H), 7.15 – 7.11 (m, 2H), 6.98 (dd, *J* = 8.4, 2.8 Hz, 1H), 6.65 (d, *J* = 8.4 Hz, 1H), 6.62 (d, *J* = 2.2 Hz, 1H), 4.51 (d, *J* = 6.6 Hz, 1H), 4.24 – 4.16 (m, 2H), 3.82 (td, *J* = 6.8, 3.4 Hz, 1H), 3.43 (dd, *J* = 11.7, 7.0 Hz, 1H), 3.33 (dd, *J* = 11.7, 3.4 Hz, 1H), 2.93 (s, 3H), 2.43 (q, *J* = 7.6 Hz, 2H), 1.27 (t, *J* = 7.2 Hz, 3H), 1.09 (t, *J* = 7.6 Hz, 3H).

^{13}C NMR (101 MHz, Chloroform-*d*) δ 194.74, 161.25, 144.46, 144.11, 133.27, 129.65, 129.13, 128.43, 126.98, 126.68, 123.31, 111.85, 62.30, 49.85, 49.77, 44.75, 39.67, 27.74, 15.78, 13.89.

HRMS (ESI): m/z Calcd. for [$C_{22}H_{26}NO_3$, M+H]$^+$:352.1907; Found: 352.1907.

ethyl 2-(*trans*-6-(tert-butyl)-1-methyl-4-phenyl-1,2,3,4-tetrahydroquinolin-3-yl)-2-oxoacetate (3c):

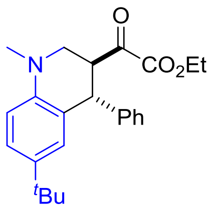

3c

Flash column chromatography (eluent: EtOAc/Hexanes = 1/10, v/v) to afford **3c** as a yellow solid, 52.2 mg, 69% yield, >20:1 dr. m.p.: 102~104 ℃. Rf = 0.47 (1:5 EtOAc/Hexanes).

^{1}H NMR (400 MHz, Chloroform-*d*) δ 7.28 – 7.24 (m, 2H), 7.22 – 7.17 (m, 1H), 7.16 – 7.09 (m, 3H), 6.77 (d, *J* = 1.5 Hz, 1H), 6.64 (d, *J* = 8.6 Hz, 1H), 4.50 (d, *J* = 6.8 Hz, 1H), 4.21 – 4.13 (m, 2H), 3.81 (td, *J* = 7.0, 3.3 Hz, 1H), 3.42 (dd, *J* = 11.4, 6.8 Hz, 1H), 3.30 (dd, *J* = 11.7, 3.3 Hz, 1H), 2.92 (s, 3H), 1.25 (t, *J* = 7.1 Hz, 3H), 1.14 (s, 9H).

^{13}C NMR (101 MHz, Chloroform-*d*) δ 194.85, 161.28, 144.34, 143.79, 140.03, 129.11, 128.38, 127.27, 126.67, 124.39, 122.89, 111.31, 62.29, 49.89, 49.75, 45.06, 39.54, 33.70, 31.34, 13.90.

HRMS (ESI): m/z Calcd. for [$C_{24}H_{30}NO_3$, M+H]$^+$:380.2220; Found: 380.2222.

ethyl 2-(*trans*-6-benzyl-1-methyl-4-phenyl-1,2,3,4-tetrahydroquinolin-3-yl)-2-oxo acetate (3d):

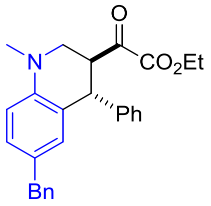

3d

Flash column chromatography (eluent: EtOAc/Hexanes = 1/10, v/v) to afford **3d** as a yellow oil, 50.6 mg, 61% yield, > 20:1 dr. Rf = 0.47 (1:5 EtOAc/Hexanes).

^{1}H NMR (400 MHz, Chloroform-*d*) δ 7.31 – 7.27 (m, 2H), 7.26 – 7.22 (m, 3H), 7.17 – 7.12 (m, 3H), 7.10 – 7.08 (m, 2H), 6.94 (dd, *J* = 8.4, 2.2 Hz, 1H), 6.68 (d, *J* = 2.0 Hz, 1H), 6.64 (d, *J* = 8.4 Hz, 1H), 4.52 (d, *J* = 6.5 Hz, 1H), 4.24 – 4.16 (m, 2H), 3.82 (td, *J* = 6.7, 3.4 Hz, 1H), 3.77 (s, 2H), 3.45 (dd, *J* = 11.4, 6.5 Hz, 1H), 3.34 (dd, *J* = 11.8, 3.4 Hz, 1H), 2.93 (s, 3H), 1.28 (t, 3H).

^{13}C NMR (101 MHz, Chloroform-*d*) δ 194.64, 161.22, 144.38, 141.84, 130.81 , 129.83, 129.08, 128.63, 128.42, 128.20, 126.70, 125.67, 123.21, 111.91, 62.31, 49.66, 44.63, 40.81, 39.56, 13.88.

HRMS (ESI): m/z Calcd. for $[C_{27}H_{28}NO_3, M+H]^+$:414.2063; Found: 414.2064.

ethyl 2-(*trans*-1-methyl-4,6-diphenyl-1,2,3,4-tetrahydroquinolin-3-yl)-2-oxo-acetate (3e):

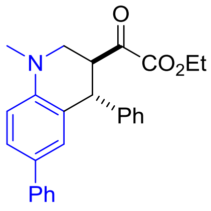

3e

Flash column chromatography (eluent: EtOAc/Hexanes = 1/10, v/v) to afford **3e** as a yellow oil, 48.0 mg, 60% yield, > 20:1 dr. Rf = 0.44 (1:5 EtOAc/Hexanes).

^{1}H NMR (400 MHz, Chloroform-*d*) δ 7.43 – 7.40 (m, 3H), 7.34 – 7.28 (m, 4H), 7.25 – 7.20 (m, 2H), 7.19 – 7.15 (m, 2H), 7.07 (d, *J* = 1.3 Hz, 1H), 6.78 (d, *J* = 8.6 Hz, 1H), 4.59 (d, *J* = 6.4 Hz, 1H), 4.26 – 4.18 (m, 2H), 3.87 (td, *J* = 6.6, 3.5 Hz, 1H), 3.52 (dd, *J* = 11.9, 6.8 Hz, 1H), 3.41 (dd, *J* = 11.9, 3.5 Hz, 1H), 3.01 (s, 3H), 1.29 (t, *J* = 7.1 Hz, 3H).

^{13}C NMR (101 MHz, Chloroform-*d*) δ 194.58, 161.18, 145.35, 144.02, 140.93, 130.01, 129.07, 128.76, 128.55, 128.51, 126.84, 126.38, 126.20, 126.00, 123.28, 111.89, 62.39, 49.48, 49.39, 44.84, 39.45, 13.90.

HRMS (ESI): m/z Calcd. for $[C_{26}H_{26}NO_3, M+H]^+$: 400.1907; Found: 400.1908.

ethyl 2-(*trans*-6-methoxy-1-methyl-4-phenyl-1,2,3,4-tetrahydroquinolin-3-yl)-2-oxoacetate (3f):

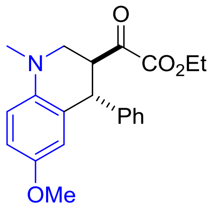

3f

Flash column chromatography (eluent: EtOAc/Hexanes = 1/8, v/v) to afford **3f** as a yellow oil, 36.1 mg, 51% yield, > 20:1 dr. Rf = 0.32 (1:5 EtOAc/Hexanes).

^{1}H NMR (400 MHz, Chloroform-*d*) δ 7.28 – 7.25 (m, 2H), 7.22 – 7.17 (m, 1H), 7.12 – 7.10 (m, 2H), 6.73 (dd, *J* = 8.6, 3.3 Hz, 1H),

6.65 (d, J = 8.9 Hz, 1H), 6.38 (d, J = 3.8 Hz, 1H), 4.50 (d, J = 6.6 Hz, 1H), 4.23 – 4.15 (m, 2H), 3.81 (td, J = 6.8, 3.4 Hz, 1H), 3.62 (s, 3H), 3.36 (dd, J = 11.7, 6.9 Hz, 1H), 3.30 (dd, J = 11.7, 3.4 Hz, 1H), 2.89 (s, 3H), 1.27 (t, J = 7.1 Hz, 3H).

^{13}C NMR (101 MHz, Chloroform-*d*) δ 194.52, 161.16, 151.95, 144.26, 140.71, 129.11, 128.48, 126.76, 124.96, 115.89, 113.33, 113.16, 62.36, 55.55, 50.17, 49.89, 44.72, 40.15, 13.91.

HRMS (ESI): m/z Calcd. for [$C_{21}H_{24}NO_4$, M+H]$^+$:354.1699; Found: 354.1698.

ethyl 2-(*trans*-6-chloro-1-methyl-4-phenyl-1,2,3,4-tetrahydroquinolin-3-yl)-2-oxo acetate (3g):

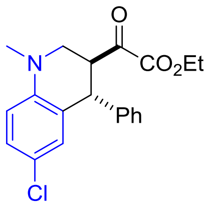

3g

Flash column chromatography (eluent: EtOAc/Hexanes = 1/8, v/v) to afford **3g** as a yellow oil, 40.3 mg, 56% yield, > 20:1 dr. Rf = 0.32 (1:5 EtOAc/Hexanes).

^{1}H NMR (400 MHz, Chloroform-*d*) δ 7.32 – 7.28 (m, 2H), 7.25 – 7.21 (m, 1H), 7.10 – 7.06 (m, 3H), 6.75 (d, J = 2.5 Hz, 1H), 6.60 (d, J = 8.8 Hz, 1H), 4.45 (d, J = 6.3 Hz, 1H), 4.25 – 4.17 (m, 2H), 3.79 (td, J = 6.4, 3.5 Hz, 1H), 3.45 (dd, J = 11.9, 6.6 Hz, 1H), 3.35 (dd, J = 12.0, 3.5 Hz, 1H), 2.93 (s, 3H), 1.28 (t, J = 7.2 Hz, 3H).

^{13}C NMR (101 MHz, Chloroform-*d*) δ 194.28, 161.07, 144.54, 143.53, 129.75, 129.04, 128.72, 127.66, 127.10, 124.64, 122.11, 112.76, 62.56, 49.37, 49.11, 44.52, 39.56, 13.97.

HRMS (ESI): m/z Calcd. for [$C_{20}H_{21}ClNO_3$, M+H]$^+$:358.1204; Found: 358.1205

ethyl 2-(*trans*-6-bromo-1-methyl-4-phenyl-1,2,3,4-tetrahydroquinolin-3-yl)-2-oxo acetate (3h):

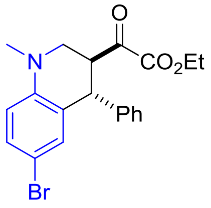

3h

Flash column chromatography (eluent: EtOAc/Hexanes = 1/8, v/v) to afford **3h** as a yellow solid, 28.1 mg, 35% yield, > 20:1 dr. m.p.: 91~92 ℃. Rf = 0.32 (1:5 EtOAc/Hexanes).

^{1}H NMR (400 MHz, Chloroform-*d*) δ 7.32 – 7.27 (m, 2H), 7.26 –

7.18 (m, 2H), 7.11 – 7.07 (m, 2H), 6.88 (dd, *J* = 2.4, 0.9 Hz, 1H), 6.55 (d, *J* = 8.8 Hz, 1H), 4.46 (d, *J* = 6.2 Hz, 1H), 4.25 – 4.17 (m, 2H), 3.78 (td, *J* = 6.4, 3.5 Hz, 1H), 3.45 (dd, *J* = 12.0, 6.5 Hz, 1H), 3.35 (dd, *J* = 12.0, 3.5 Hz, 1H), 2.93 (s, 3H), 1.28 (t, *J* = 7.1 Hz, 3H).

^{13}C NMR (101 MHz, Chloroform-*d*) δ 194.17, 161.01, 144.85, 143.44, 132.45, 130.49, 128.95, 128.65, 127.04, 124.94, 113.12, 109.20, 62.49, 49.16, 48.97, 44.38, 39.41, 13.90.

HRMS (ESI): m/z Calcd. for [$C_{20}H_{21}BrNO_3$, M+H]$^+$:402.0699; Found: 402.0699.

ethyl 2-oxo-2-(*trans*-1,6,7-trimethyl-4-phenyl-1,2,3,4-tetrahydroquinolin-3-yl)acetate (3i):

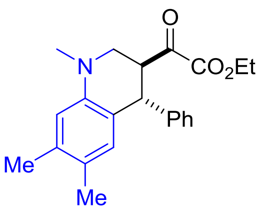

3i

Flash column chromatography (eluent: EtOAc/Hexanes = 1/10, v/v) to afford **3i** as a yellow oil, 40.0mg, 57% yield, > 20:1 dr. Rf = 0.48 (1:5 EtOAc/Hexanes).

^{1}H NMR (400 MHz, Chloroform-*d*) δ 7.29 – 7.25 (m, 2H), 7.23 – 7.18 (m, 1H), 7.14– 7.10 (m, 2H), 6.52 (d, *J* = 6.7 Hz, 2H), 4.46 (d, *J* = 6.5 Hz, 1H), 4.23 – 4.14 (m, 2H), 3.78 (td, *J* = 6.7, 3.3 Hz, 1H), 3.39 (dd, *J* = 12.1, 7.3 Hz, 1H), 3.30 (dd, *J*= 11.7, 3.3 Hz, 1H), 2.91 (s, 3H), 2.21 (s, 3H), 2.03 (s, 3H), 1.26 (t, *J* = 7.1 Hz, 3H).

^{13}C NMR (101 MHz, Chloroform-*d*) δ 194.75, 161.28, 144.65, 144.16, 135.72, 131.23, 129.09, 128.41, 126.63, 125.56, 120.81, 113.41, 62.28, 49.87, 49.84, 44.30, 39.75, 19.94, 18.57, 13.90.

HRMS (ESI): m/z Calcd. for [$C_{22}H_{26}NO_3$, M+H]$^+$: 352.1907; Found: 352.1907.

ethyl 2-(*trans*-6,7-dimethoxy-1-methyl-4-phenyl-1,2,3,4-tetrahydroquinolin-3-yl)-2-oxoacetate (3j):

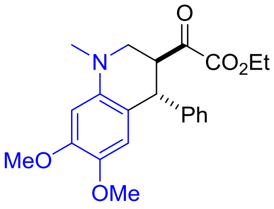

3j

Flash column chromatography (eluent: EtOAc/Hexanes = 1/5, v/v) to afford **3j** as a yellow oil, 56.8mg, 74% yield, > 20:1 dr. Rf = 0.16 (1:5 EtOAc/Hexanes).

^{1}H NMR (400 MHz, Chloroform-*d*) δ 7.30 – 7.26 (m, 2H), 7.23 – 7.19 (m, 1H), 7.13– 7.10 (m, 2H), 6.34 (s, 1H), 6.32 (s, 1H), 4.48 (d, *J* = 5.9 Hz, 1H), 4.26 – 4.18 (m, 2H), 3.88 (s, 3H), 3.73 (td, *J* = 6.2, 3.1 Hz,1H), 3.63 (s, 3H), 3.39 (dd, *J* = 11.8, 6.5 Hz, 1H), 3.29 (dd, *J* = 11.8, 3.1 Hz, 1H), 2.93 (s, 3H), 1.29 (t, *J* = 7.2 Hz, 3H).

^{13}C NMR (101 MHz, Chloroform-*d*) δ 194.48, 161.24, 148.61, 144.75, 141.50, 140.77, 128.97, 128.41, 126.68, 114.61, 114.33, 97.53, 62.32, 56.42, 55.79, 49.93, 49.68, 43.92, 40.26, 13.90.

HRMS (ESI): m/z Calcd. for [$C_{22}H_{26}NO_5$, M+H]$^+$: 384.1805; Found: 384.1805.

ethyl 2-(1,6-dimethyl-2,4-diphenyl-1,2,3,4-tetrahydroquinolin-3-yl)-2-oxoacetate (3k):

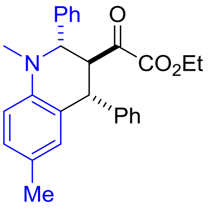

3k

Flash column chromatography (eluent: EtOAc/Hexanes = 1/10, v/v) to afford **3k** as a yellow solid, 45.2 mg, 55% yield. m.p.: 145~146 ℃. Rf = 0.53 (1:5 EtOAc/Hexanes).

^{1}H NMR (400 MHz, Chloroform-*d*) δ 7.32 – 7.21 (m, 10H), 7.01 (dd, *J* = 8.2, 2.1 Hz, 1H), 6.74 (d, *J* = 8.3 Hz, 1H), 6.39 (s, 1H), 4.55 – 4.45 (m, 2H), 4.40 (dd, *J* = 10.5, 1.3 Hz, 1H), 3.79 – 3.72 (m, 2H), 2.72 (s, 3H), 2.13 (s, 3H), 0.97 (t, *J* = 7.1 Hz, 3H).

^{13}C NMR (101 MHz, Chloroform-*d*) δ 196.09, 159.66, 144.43, 140.19, 139.78, 129.56, 128.84, 128.71, 128.59, 128.31, 128.04, 127.95, 127.17, 126.25, 126.09, 112.47, 66.56, 61.97, 56.05, 47.65, 37.00, 20.30, 13.61.

HRMS (ESI): m/z Calcd. for [$C_{27}H_{28}NO_3$, M+H]$^+$:414.2063; Found: 414.2064.

ethyl 2-(1,6-dimethyl-2,4-diphenyl-1,2,3,4-tetrahydroquinolin-3-yl)-2-oxoacetate (3k'):

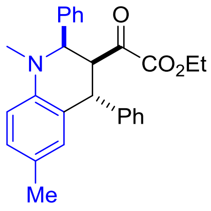

3k'

Flash column chromatography (eluent: EtOAc/Hexanes = 1/10, v/v) to afford **3k'** as a yellow solid, 10.0 mg, 12% yield. m.p.: 140~141 ℃. Rf = 0.44 (1:5 EtOAc/Hexanes).

^{1}H NMR (400 MHz, Chloroform-*d*) δ 7.27 – 7.22 (m, 5H), 7.20 – 7.17 (m, 3H), 7.02 – 6.95 (m, 1H), 6.94 – 6.91 (m, 2H), 6.64 (d, *J* = 8.3 Hz, 1H), 6.41 (s, 1H), 4.94 (d, *J* = 4.5 Hz, 1H), 4.57 (dd, *J* = 11.7, 4.5 Hz, 1H), 4.31 – 4.20 (m, 3H), 2.94 (s, 3H), 2.11 (s, 3H), 1.31 (t, *J* = 7.1 Hz, 3H).

^{13}C NMR (101 MHz, Chloroform-*d*) δ 192.15, 160.75, 143.21, 143.00, 138.39, 130.58, 129.71, 128.42, 128.37, 127.96, 127.23, 126.59, 125.65, 124.22, 110.43, 63.79, 62.50, 54.04, 40.33, 38.56, 20.32, 13.89.

HRMS (ESI): m/z Calcd. for $[C_{27}H_{28}NO_3, M+H]^+$:414.2063; Found: 414.2063.

ethyl 2-(1,6-dimethyl-4-phenyl-2-vinyl-1,2,3,4-tetrahydroquinolin-3-yl)-2-oxoacetate (3l):

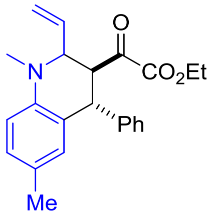

3l

Flash column chromatography (eluent: EtOAc/Hexanes = 1/10, v/v) to afford **3l** as a mixture of diastereomers, yellow oil, 51.1 mg, 70% yield, 1.1:1 dr. Rf = 0.53 (1:5 EtOAc/Hexanes).

Major diastereomer **^{1}H NMR** (400 MHz, Chloroform-*d*) δ 7.32 – 7.17 (m, 5H), 6.97 – 6.95 (m, 1H), 6.68 (d, *J* = 8.3 Hz, 1H), 6.38 (s, 1H), 5.74 – 5.64 (m,1H), 5.21 – 5.15 (m, 2H), 4.30 (d, *J* = 10.9 Hz, 1H), 4.26 – 4.17 (m, 1H), 4.10 – 4.02 (m, 2H), 3.94 (t, *J* = 9.1 Hz, 1H), 2.90 (s, 3H), 2.10 (s, 3H), 1.16 (t, *J* = 7.1 Hz, 3H).

Major diastereomer **^{13}C NMR** (101 MHz, Chloroform-*d*) δ 195.72, 160.67, 144.03, 142.39, 137.90, 130.77, 129.64, 128.69, 128.38, 127.21, 126.42, 125.72, 119.32, 112.45, 63.30, 62.35, 53.53, 40.90, 36.25, 20.35, 13.94.

Minor diastereomer **^{1}H NMR** (400 MHz, Chloroform-*d*) δ 7.32 – 7.17 (m, 5H), 6.92 (dd, *J* = 8.6, 1.8 Hz, 1H), 6.59 (d, *J* = 8.3 Hz, 1H), 6.44 (s, 1H), 5.74 – 5.64 (m, 1H), 5.15 – 5.13 (m, 1H), 5.01 (dt, *J* = 17.0, 1.2 Hz, 1H), 4.48 (d, *J* = 11.9 Hz, 1H), 4.36 (dd, *J* = 11.9, 3.9 Hz, 1H), 4.26 – 4.17 (m, 3H), 2.94 (s, 3H), 2.08 (s, 3H), 1.29 (t, *J* = 7.1 Hz, 3H).

Minor diastereomer **^{13}C NMR** (101 MHz, Chloroform-*d*) δ 192.67, 160.44, 143.65, 140.71, 132.24, 129.56, 128.97, 128.48, 128.22, 126.66, 126.18, 124.76, 119.01, 111.70, 63.30, 62.35, 53.53, 40.90, 36.25, 20.35, 13.85.

HRMS (ESI): m/z Calcd. for [$C_{23}H_{26}NO_3$, M+H]$^+$: 364.1907; Found: 364.1910.

ethyl 2-(2-ethynyl-1,6-dimethyl-4-phenyl-1,2,3,4-tetrahydroquinolin-3-yl)-2-oxoacetate (3m):

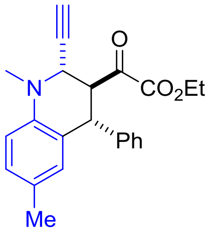

3m

Flash column chromatography (eluent: EtOAc/Hexanes = 1/10, v/v) to afford **3m** as a yellow oil, 41.2 mg, 57% yield, 6:1 dr. Rf = 0.41 (1:5 EtOAc/Hexanes).

^{1}H NMR (400 MHz, Chloroform-*d*) δ 7.32 – 7.19 (m, 5H), 6.98 (dd, *J* = 8.3, 1.5 Hz, 1H), 6.72 (d, *J* = 8.3 Hz, 1H), 6.44 (s, 1H), 4.37 – 4.24 (m, 3H), 4.12 – 4.05 (m, 2H), 3.04 (s, 3H), 2.31 (d, *J* = 2.0 Hz, 1H), 2.11 (s, 3H), 1.18 (t, *J* = 7.1 Hz, 3H).

^{13}C NMR (101 MHz, Chloroform-*d*) δ 194.65, 160.37, 142.99, 140.38, 129.59, 128.98, 128.58, 128.36, 127.60, 127.31, 126.03, 113.46, 80.56, 74.41, 62.44, 54.35, 52.50, 46.50, 36.97, 20.36, 13.79.

HRMS (ESI): m/z Calcd. for [$C_{23}H_{24}NO_3$, M+H]$^+$:362.1750; Found: 362.1755.

ethyl 2-(7-methyl-5-phenyl-1,2,3,3a,4,5-hexahydropyrrolo[1,2-a]quinolin-4-yl)-2-oxoacetate (3n):

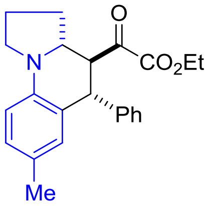

3n

Flash column chromatography (eluent: EtOAc/Hexanes = 1/10, v/v) to afford **3n** as a yellow solid, 37.9 mg, 52% yield, > 20:1 dr. m.p.: 108~110 ℃. Rf = 0.54 (1:5 EtOAc/Hexanes).

^{1}H NMR (400 MHz, Chloroform-*d*) δ 7.31 – 7.21 (m, 3H), 7.19 – 7.16 (m, 2H), 6.96 – 6.90 (m, 1H), 6.46 (d, *J* = 8.1 Hz, 1H), 6.35 (s, 1H), 4.22 (d, *J* = 11.3 Hz, 1H), 4.07 – 4.00 (m, 2H), 3.74 (dd, *J* = 11.3, 9.9 Hz, 1H), 3.66 (td, *J* = 9.8, 4.8 Hz, 1H), 3.45 (td, *J* = 9.0, 2.3 Hz, 1H), 3.32 (td, *J* = 8.9, 7.3 Hz, 1H), 2.14 – 1.93 (m, 6H), 1.69 – 1.61 (m, 1H), 1.15 (t, *J* = 7.1 Hz, 3H).

^{13}**C NMR** (101 MHz, Chloroform-*d*) δ 197.96, 160.74, 141.58, 141.07, 129.38, 129.24, 128.58, 128.24, 127.12, 124.40, 124.18, 110.63, 62.30, 60.06, 52.80, 48.99, 47.29, 30.93, 23.64, 20.34, 13.76.

HRMS (ESI): m/z Calcd. for $[C_{23}H_{26}NO_3, M+H]^+$: 364.1907; Found: 364.1906.

ethyl 2-(8-methyl-6-phenyl-2,3,4,4a,5,6-hexahydro-1H-pyrido[1,2-a]quinolin-5-yl)-2-oxoacetate (3o):

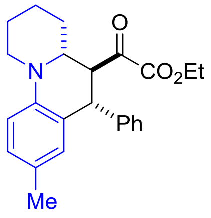

3o

Flash column chromatography (eluent: EtOAc/Hexanes = 1/10, v/v) to afford **3o** as a yellow solid, 32.8 mg, 44% yield, > 20:1 dr. m.p.: 126~128 ℃. Rf = 0.54 (1:5 EtOAc/Hexanes).

^{1}**H NMR** (400 MHz, Chloroform-*d*) δ 7.31 – 7.21 (m, 3H), 7.18 – 7.16 (m, 2H), 6.95 (dd, *J* = 8.6, 2.2 Hz, 1H), 6.84 (d, *J* = 8.5 Hz, 1H), 6.41 (s, 1H), 4.27 (d, *J* = 11.7 Hz, 1H), 4.12 – 4.02 (m, 3H), 3.97 (dd, *J* = 12.1, 3.2 Hz, 1H), 3.30 (td, *J* = 10.1, 2.8 Hz, 1H), 2.74 (td, *J* = 12.2, 2.9 Hz, 1H), 2.09 (s, 3H), 1.84 – 1.62 (m, 4H), 1.45 – 1.36 (m, 2H), 1.15 (t, *J* = 7.1 Hz, 3H).

^{13}**C NMR** (101 MHz, Chloroform-*d*) δ 197.76, 160.67, 144.06, 141.41, 129.51, 129.33, 128.55, 128.16, 127.03, 126.97, 126.64, 113.42, 62.25, 58.84, 55.08, 48.53, 48.32, 30.77, 25.33, 23.51, 20.18, 13.73.

HRMS (ESI): m/z Calcd. for $[C_{24}H_{27}NO_3Na, M+Na]^+$: 400.1883; Found: 400.1883.

ethyl 2-(3-methyl-5-phenyl-5,6,6a,7,8,9,10,11-octahydroazepino[1,2-a]quinolin-6-yl)-2-oxoacetate (3p):

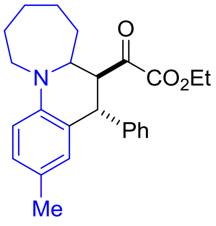

3p

Flash column chromatography (eluent: EtOAc/Hexanes = 1/10, v/v) to afford **3p** as a mixture of diastereomers, yellow solid, 52.5 mg, 67% yield, 3:1 dr. m.p.: 113~114 ℃. Rf = 0.53 (1:5 EtOAc/Hexanes).

Major diastereomer ^{1}**H NMR** (400 MHz, Chloroform-*d*) δ 7.30 – 7.15 (m, 5H), 6.88 (dd, *J* = 8.3, 2.2 Hz, 1H), 6.56 (d, *J* = 8.4 Hz, 1H), 6.42 (s, 1H), 4.52 (d, *J* = 12.1 Hz, 1H), 4.26 – 4.18 (m, 3H), 4.03 – 3.97 (m,1H), 3.84 (dt, *J* = 11.2,

3.9 Hz, 1H), 3.31 – 3.23 (m, 1H), 2.06 (s, 3H), 1.79 – 1.31 (m, 8H), 1.28 (t, *J* = 7.2 Hz, 3H).

Major diastereomer **^{13}C NMR** (101 MHz, Chloroform-*d*) δ 193.90, 160.80, 144.50, 141.08, 131.01, 129.64, 128.31, 128.12, 126.46, 124.76, 122.95, 110.54, 62.41, 58.77, 53.93, 50.30, 40.59, 29.97, 27.05, 25.95, 25.83, 20.25, 13.86.

Minor diastereomer **^{1}H NMR** (400 MHz, Chloroform-*d*) δ 7.30 – 7.15 (m, 5H), 6.92 (dd, *J* = 7.7, 1.9 Hz, 1H), 6.66 (d, *J* = 8.3 Hz, 1H), 6.39 (s, 1H), 4.26 – 4.18 (m, 1H), 4.10 – 4.14 (m, 2H), 3.95 – 3.93 (m, 1H), 3.80 – 3.76 m, 1H), 3.59 (ddd, *J* = 14.9, 6.3, 2.4 Hz, 1H), 3.35 (ddd, *J* = 15.2, 9.5, 2.5 Hz, 1H), 2.09 (s, 3H), 1.79 – 1.31 (m, 8H), 1.17 (t, *J* = 7.2 Hz, 3H).

Minor diastereomer **^{13}C NMR** (101 MHz, Chloroform-*d*) δ 197.54, 161.07, 144.10, 141.50, 129.39, 129.20, 128.57, 128.23, 126.98, 125.30, 112.68, 62.28, 60.07, 53.11, 49.27, 47.93, 33.13, 28.91, 28.86, 24.22, 13.80.

HRMS (ESI): m/z Calcd. for [$C_{25}H_{30}NO_3$, M+H]$^+$:392.2220; Found: 392.2220.

ethyl 2-(*trans*-1,6-dimethyl-4-(o-tolyl)-1,2,3,4-tetrahydroquinolin-3-yl)-2-oxo-acetate (3q):

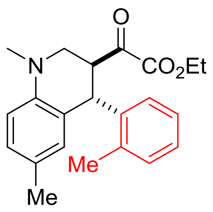

3q

Flash column chromatography (eluent: EtOAc/Hexanes = 1/10, v/v) to afford **3q** as a yellow oil, 43.4 mg, 62% yield, > 20:1 dr. Rf = 0.55 (1:5 EtOAc/Hexanes).

^{1}H NMR (400 MHz, Chloroform-*d*) δ 7.19 – 7.07 (m, 3H), 6.94 (dd, *J* = 8.4, 2.1 Hz, 1H), 6.89 (dd, *J* = 7.4, 1.7 Hz, 1H), 6.62 (d, *J* = 8.3 Hz, 1H), 6.52 (s, 1H), 4.70 (d, *J* = 6.4 Hz, 1H), 4.23 – 4.15 (m, 2H), 3.77 (td, *J* = 6.6, 3.4 Hz, 1H), 3.45 (dd, *J* = 11.8, 6.9 Hz, 1H), 3.36 (dd, *J* = 11.7, 3.4 Hz, 1H), 2.93 (s, 3H), 2.38 (s, 3H), 2.12 (s, 3H), 1.27 (t, *J* = 7.1 Hz, 3H).

^{13}C NMR (101 MHz, Chloroform-*d*) δ 194.96, 161.35, 143.99, 142.33, 136.04, 130.48, 130.27, 129.93, 128.11, 126.81, 126.59, 126.05, 123.74, 111.80, 62.28, 49.90, 47.88, 40.95, 39.66, 20.25, 19.51, 13.88.

HRMS (ESI): m/z Calcd. for [$C_{22}H_{26}NO_3$, M+H]$^+$:352.1907; Found: 352.1906.

ethyl 2-(*trans*-4-(2-methoxyphenyl)-1,6-dimethyl-1,2,3,4-tetrahydroquinolin-3-yl)-2-oxoacetate (3r):

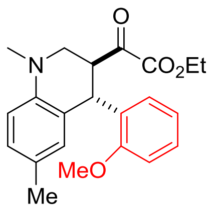

3r

Flash column chromatography (eluent: EtOAc/Hexanes = 1/10, v/v) to afford **3r** as a yellow solid, 42.8 mg, 58% yield, > 20:1 dr. m.p.: 103~104 ℃. Rf = 0.40 (1:5 EtOAc/Hexanes).

^{1}H NMR (400 MHz, Chloroform-*d*) δ 7.23 – 7.19 (m, 1H), 6.94 (d, *J* = 8.4 Hz, 1H), 6.89 – 6.82 (m, 2H), 6.75 (d, *J* = 7.5 Hz, 1H), 6.68 (s, 1H), 6.62 (d, *J* = 8.4 Hz, 1H), 4.90 (d, *J* = 4.1 Hz, 1H), 4.29 – 4.21 (m, 2H), 3.84 (s, 3H), 3.67 (q, *J* = 4.3, 3.7 Hz, 1H), 3.47 (dd, *J* = 12.0, 5.3 Hz, 1H), 3.27 (dd, *J* = 11.9, 3.0 Hz, 1H), 2.88 (s, 3H), 2.16 (s, 3H), 1.31 (t, *J* = 7.2 Hz, 3H).

^{13}C NMR (101 MHz, Chloroform-*d*) δ 194.62, 161.76, 156.50, 144.39, 133.34, 131.12, 130.76, 128.01, 127.62, 126.82, 122.72, 120.24, 111.80, 110.08, 62.05, 55.28, 49.10, 47.56, 39.61, 37.57, 20.25, 13.94.

HRMS (ESI): m/z Calcd. for $[C_{22}H_{26}NO_4, M+H]^+$:368.1856; Found: 368.1859.

ethyl 2-(*trans*-4-(2-chlorophenyl)-1,6-dimethyl-1,2,3,4-tetrahydroquinolin-3-yl)-2-oxoacetate (3s):

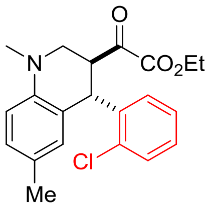

3s

Flash column chromatography (eluent: EtOAc/Hexanes = 1/10, v/v) to afford **3s** as a yellow oil, 53.4 mg, 72% yield, > 20:1 dr. Rf = 0.52 (1:5 EtOAc/Hexanes).

^{1}H NMR (400 MHz, Chloroform-*d*) δ 7.40 – 7.38 (m, 1H), 7.19 – 7.11 (m, 2H), 6.96 (dd, *J* = 8.4, 2.3 Hz, 1H), 6.85 (dd, *J* = 7.3, 2.2 Hz, 1H), 6.65 – 6.62 (m, 2H), 5.00 (d, *J* = 3.7 Hz, 1H), 4.32 – 4.24 (m, 2H), 3.68 (q, *J* = 3.5 Hz, 1H), 3.51 (ddd, *J* = 12.1, 4.9, 1.3 Hz, 1H), 3.28 (dd, *J* = 12.1, 3.1 Hz, 1H), 2.89 (s, 3H), 2.16 (s, 3H), 1.33 (t, *J* = 7.1 Hz, 3H).

^{13}C NMR (101 MHz, Chloroform-*d*) δ 193.64, 161.45, 144.17, 142.56, 133.42, 131.96, 130.85, 129.52, 128.49, 127.87, 127.19, 126.65, 121.65, 112.04, 62.27, 48.57, 47.52, 40.31, 39.59, 20.23, 13.95.

HRMS (ESI): m/z Calcd. for $[C_{21}H_{22}ClNO_3Na, M+Na]^+$:394.1180; Found: 394.1180.

ethyl 2-(*trans*-1,6-dimethyl-4-(m-tolyl)-1,2,3,4-tetrahydroquinolin-3-yl)-2-oxoacetate (3t):

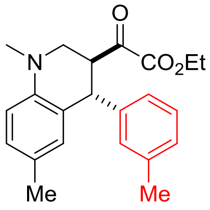

3t

Flash column chromatography (eluent: EtOAc/Hexanes = 1/10, v/v) to afford **3t** as a yellow oil, 46.6 mg, 66% yield, > 20:1 dr. Rf = 0.55 (1:5 EtOAc/Hexanes).

^{1}H NMR (400 MHz, Chloroform-*d*) δ 7.17 (t, *J* = 7.6 Hz, 1H), 7.04 (d, *J* = 7.6 Hz, 1H), 6.96 – 6.93 (m, 2H), 6.90 (d, *J* = 7.7 Hz, 1H), 6.63 (d, *J* = 8.4 Hz, 2H), 4.45 (d, *J* = 6.5 Hz, 1H), 4.25 – 4.17 (m, 2H), 3.80 (td, *J* = 6.6, 3.4 Hz, 1H), 3.41 (dd, *J* = 11.7, 6.8 Hz, 1H), 3.33 (dd, *J* = 11.7, 3.4 Hz, 1H), 2.92 (s, 3H), 2.31 (s, 3H), 2.14 (s, 3H), 1.28 (t, *J* = 7.1 Hz, 3H).

^{13}C NMR (101 MHz, Chloroform-*d*) δ 194.74, 161.27, 144.48, 143.92, 138.02, 130.74, 129.73, 128.30, 128.21, 127.45, 126.67, 126.27, 123.43, 111.85, 62.28, 49.83, 49.78, 44.54, 39.70, 21.39, 20.26, 13.89.

HRMS (ESI): m/z Calcd. for $[C_{22}H_{26}NO_3, M+H]^+$:352.1907; Found: 352.1907.

ethyl 2-(*trans*-4-(3-bromophenyl)-1,6-dimethyl-1,2,3,4-tetrahydroquinolin-3-yl)-2-oxoacetate (3u):

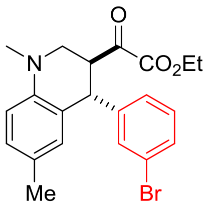

3u

Flash column chromatography (eluent: EtOAc/Hexanes = 1/10, v/v) to afford **3u** as a yellow oil, 57.2 mg, 69% yield, > 20:1 dr. Rf = 0.51 (1:5 EtOAc/Hexanes).

^{1}H NMR (400 MHz, Chloroform-*d*) δ 7.36 (ddd, *J* = 7.9, 2.0, 1.1 Hz, 1H), 7.29 (t, *J* = 1.9 Hz, 1H), 7.15 (t, *J* = 7.8 Hz, 1H), 7.04 (dt, *J* = 7.8, 1.5 Hz, 1H), 6.95 (dd, *J* = 8.4, 2.2 Hz, 1H), 6.62 (d, *J* = 8.4 Hz, 1H), 6.57 (s, 1H), 4.48 (d, *J* = 6.4 Hz, 1H), 4.28 – 4.20 (m, 2H), 3.76 (td, *J* = 6.6, 3.4 Hz, 1H), 3.39 (dd, *J* = 11.8, 6.7 Hz, 1H), 3.32 (dd, *J* = 11.8, 3.5 Hz, 1H), 2.91 (s, 3H), 2.14 (s, 3H), 1.30 (t, *J* = 7.2 Hz, 3H).

^{13}C NMR (101 MHz, Chloroform-*d*) δ 194.09, 161.11, 147.10, 143.87, 131.95, 130.69, 130.03, 129.90, 128.57, 127.89, 126.94, 122.59, 122.45, 112.10, 62.48, 49.75, 49.71, 44.08, 39.68, 20.26, 13.91.

HRMS (ESI): m/z Calcd. for $[C_{21}H_{23}BrNO_3, M+H]^+$:416.0855; Found: 416.0855.

ethyl 2-(*trans*-1,6-dimethyl-4-(p-tolyl)-1,2,3,4-tetrahydroquinolin-3-yl)-2-oxo-acetate (3v):

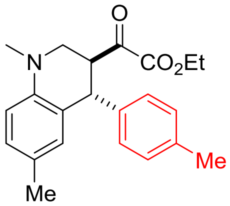

3v

Flash column chromatography (eluent: EtOAc/Hexanes = 1/10, v/v) to afford **3v** as a yellow oil, 42.9 mg, 61% yield, > 20:1 dr. Rf = 0.54 (1:5 EtOAc/Hexanes).

^{1}H NMR (400 MHz, Chloroform-*d*) δ7.09 (d, *J* = 7.9 Hz, 2H), 7.00 (d, *J* = 8.1 Hz, 2H), 6.94 (dd, *J* = 8.3, 2.1 Hz, 1H), 6.62 – 6.60 (m, 2H), 4.45 (d, *J* = 6.4 Hz, 1H), 4.24 – 4.16 (m, 2H), 3.77 (td, *J* = 6.6, 3.3 Hz, 1H), 3.41 (dd, *J* = 11.7, 6.8 Hz, 1H), 3.32 (dd, *J* = 11.7, 3.4 Hz, 1H), 2.91 (s, 3H), 2.32 (s, 3H), 2.12 (s, 3H), 1.27 (t, *J* = 7.1 Hz, 3H).

^{13}C NMR (101 MHz, Chloroform-*d*) δ 194.76, 161.28, 143.91, 141.50, 136.23, 130.67, 129.12, 128.98, 128.19, 126.64, 123.48, 111.84, 62.26, 49.81, 49.75, 44.18, 39.68, 20.96, 20.24, 13.87.

HRMS (ESI): m/z Calcd. for $[C_{22}H_{26}NO_3, M+H]^+$:352.1907; Found: 352.1907.

ethyl 2-(*trans*-4-(4-(tert-butyl)phenyl)-1,6-dimethyl-1,2,3,4-tetrahydroquinolin-3-yl)-2-oxoacetate (3w):

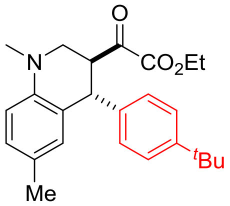

3w

Flash column chromatography (eluent: EtOAc/Hexanes = 1/10, v/v) to afford **3w** as a yellow oil, 47.3 mg, 60% yield, > 20:1 dr. Rf = 0.59 (1:5 EtOAc/Hexanes).

^{1}H NMR (400 MHz, $CDCl_3$) δ 7.31 – 7.27 (m, 2H), 7.04 – 7.02 (m, 2H), 6.94 (dd, *J* = 8.4, 2.1 Hz, 1H), 6.65 (s, 1H), 6.62 (d, *J* = 8.3 Hz, 1H), 4.46 (d, *J* = 5.9 Hz, 1H), 4.23 – 4.15 (m, 2H), 3.75 (td, *J* = 6.2, 3.3 Hz, 1H), 3.43 (dd, *J* = 11.3, 6.0 Hz, 1H), 3.32 (dd, *J* = 11.8, 3.3 Hz, 1H), 2.91 (s, 3H), 2.14 (s, 3H), 1.30 (s, 9H), 1.28 (t, *J* = 7.1 Hz, 3H).

^{13}C NMR (101 MHz, Chloroform-*d*) δ 194.84, 161.33, 149.38, 143.90, 141.46, 130.75, 128.66, 128.21, 126.62, 125.29, 123.25, 111.81, 62.23, 49.70, 49.51, 44.01, 39.67, 34.37, 31.32, 20.27, 13.93.

HRMS (ESI): m/z Calcd. for $[C_{25}H_{32}NO_3, M+H]^+$:394.2376; Found: 394.2376.

ethyl 2-(*trans*-4-([1,1'-biphenyl]-4-yl)-1,6-dimethyl-1,2,3,4-tetrahydroquinolin-3-yl)-2-oxoacetate (3x):

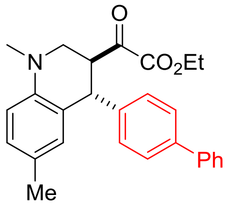

3x

Flash column chromatography (eluent: EtOAc/Hexanes = 1/10, v/v) to afford **3x** as a yellow oil, 50.5 mg, 61% yield, > 20:1 dr. Rf = 0.49 (1:5 EtOAc/Hexanes).

^{1}**H NMR** (400 MHz, Chloroform-*d*) δ 7.61 – 7.58 (m, 2H), 7.56 – 7.52 (m, 2H), 7.46 – 7.43 m, 2H), 7.38 – 7.33 (m, 1H), 7.22 (d, *J* = 8.2 Hz, 2H), 6.98 (dd, *J* = 8.4, 2.2 Hz, 1H), 6.70 – 6.64 (m, 2H), 4.57 (d, *J* = 6.4 Hz, 1H), 4.27 – 4.19 (m, 2H), 3.86 (td, *J* = 6.6, 3.4 Hz, 1H), 3.46 (dd, *J* = 11.7, 6.7 Hz, 1H), 3.39 (dd, *J* = 11.7, 3.4 Hz, 1H), 2.95 (s, 3H), 2.17 (s, 3H), 1.29 (t, *J* = 7.2 Hz, 3H).

^{13}**C NMR** (101 MHz, Chloroform-*d*) δ 194.61, 161.26, 143.93, 143.64, 140.66, 139.53, 130.73, 129.52, 128.73, 128.34, 127.20, 127.12, 126.93, 126.75, 123.18, 111.95, 62.33, 49.76, 44.22, 39.68, 20.26, 13.89.

HRMS (ESI): m/z Calcd. for $[C_{27}H_{28}NO_3, M+H]^+$:414.2063; Found: 414.2062.

ethyl 2-(*trans*-4-(4-methoxyphenyl)-1,6-dimethyl-1,2,3,4-tetrahydroquinolin-3-yl) -2-oxoacetate (3y):

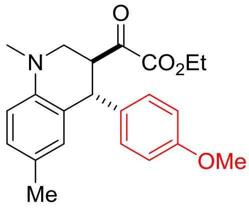

3y

Flash column chromatography (eluent: EtOAc/Hexanes = 1/10, v/v) to afford **3y** as a yellow oil, 41.0 mg, 56% yield, > 20:1 dr. Rf = 0.46 (1:5 EtOAc/Hexanes).

^{1}**H NMR** (400 MHz, Chloroform-d) δ 7.06 – 7.02 (m, 2H), 6.93 (dd, J = 8.4, 2.1 Hz, 1H), 6.84 – 6.80 (m, 2H), 6.63 – 6.58 (m, 2H), 4.42 (d, J = 6.9 Hz, 1H), 4.23 – 4.15 (m, 2H), 3.81 – 3.77 (m, 4H), 3.40 (dd, J = 11.7, 7.1 Hz, 1H), 3.32 (dd, J = 11.7, 3.4 Hz, 1H), 2.92 (s, 3H), 2.13 (s, 3H), 1.27 (t, J = 7.2 Hz, 3H).

^{13}**C NMR** (101 MHz, Chloroform-d) δ 194.92, 161.22, 158.32, 143.86, 136.33, 130.59, 130.09, 128.19, 126.61, 123.78, 113.80, 111.85, 62.31, 55.19, 49.99, 49.78, 44.01, 39.69, 20.27, 13.90.

HRMS (ESI): m/z Calcd. for $[C_{22}H_{26}NO_4, M+H]^+$:368.1856; Found: 368.1857

ethyl 2-(*trans*-4-(4-fluorophenyl)-1,6-dimethyl-1,2,3,4-tetrahydroquinolin-3-yl)-2-oxoacetate (3z):

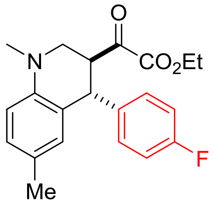

3z

Flash column chromatography (eluent: EtOAc/Hexanes = 1/10, v/v) to afford **3z** as a yellow oil, 46.2 mg, 65% yield, > 20:1 dr. Rf = 0.50 (1:5 EtOAc/Hexanes).

^{1}H NMR (400 MHz, Chloroform-*d*) δ 7.11 – 7.07 (m, 2H), 7.00 – 6.93 (m, 3H), 6.62 (d, *J* = 8.3 Hz, 1H), 6.55 (s, 1H), 4.47 (d, *J* = 6.9 Hz, 1H), 4.26 – 4.18 (m, 2H), 3.79 (td, *J* = 7.0, 3.4 Hz, 1H), 3.39 (dd, *J* = 11.7, 7.0 Hz, 1H), 3.32 (dd, *J* = 11.7, 3.5 Hz, 1H), 2.92 (s, 3H), 2.13 (s, 3H), 1.29 (t, *J* = 7.2 Hz, 3H).

^{13}C NMR (101 MHz, Chloroform-*d*) δ 194.49, 161.64 (d, *J* = 245.9 Hz),161.14, 143.85, 140.13 (d, *J* = 3.0 Hz), 130.59, 130.58 (d, *J* = 7.9 Hz), 128.41, 126.77, 123.25, 115.3 (d, *J* = 21.6 Hz), 112.00, 62.43, 49.94, 49.80, 43.93, 39.70, 20.25, 13.90.

^{19}F NMR (377 MHz, Chloroform-*d*) δ -116.11.

HRMS (ESI): m/z Calcd. for [$C_{21}H_{23}FNO_3$, M+H]$^+$:356.1656; Found: 356.1652.

ethyl 2-(*trans*-4-(4-chlorophenyl)-1,6-dimethyl-1,2,3,4-tetrahydroquinolin-3-yl)-2-oxoacetate (3aa):

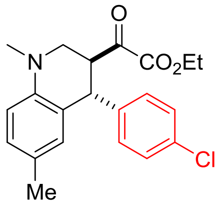

3aa

Flash column chromatography (eluent: EtOAc/Hexanes = 1/10, v/v) to afford **3aa** as a yellow oil, 54.3 mg, 73% yield, > 20:1 dr. Rf = 0.47 (1:5 EtOAc/Hexanes).

^{1}H NMR (400 MHz, Chloroform-*d*) δ 7.27 – 7.24 (m, 2H), 7.08 – 7.05 (m, 2H), 6.95 (dd, *J* = 8.4, 2.1 Hz, 1H), 6.63 (d, *J* = 8.3 Hz, 1H), 6.56 (s, 1H), 4.48 (d, *J* = 6.8 Hz, 1H), 4.27 – 4.19 (m, 2H), 3.78 (td, *J* = 6.9, 3.5 Hz, 1H), 3.42 – 3.36 (m, 1H), 3.32 (dd, *J* = 11.7, 3.5 Hz, 1H), 2.92 (s, 3H), 2.13 (s, 3H), 1.30 (t, *J* = 7.2 Hz, 3H).

^{13}C NMR (101 MHz, Chloroform-*d*) δ 194.25, 161.07, 143.85, 143.03, 132.49, 130.58, 130.45, 128.60, 128.46, 126.81, 122.86, 112.02, 62.46, 49.85, 49.70, 43.91, 39.67, 20.23, 13.88.

HRMS (ESI): m/z Calcd. for [$C_{21}H_{23}ClNO_3$, M+H]$^+$:372.1361; Found: 372.1361.

ethyl 2-(*trans*-4-(4-bromophenyl)-1,6-dimethyl-1,2,3,4-tetrahydroquinolin-3-yl)-2-oxoacetate (3ab):

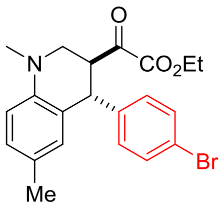
3ab

Flash column chromatography (eluent: EtOAc/Hexanes = 1/10, v/v) to afford **3ab** as a yellow oil, 57.1 mg, 68% yield, > 20:1 dr. Rf = 0.47 (1:5 EtOAc/Hexanes).

^{1}H NMR (400 MHz, Chloroform-*d*) δ 7.42 – 7.39 (m, 2H), 7.02 – 6.99 (m, 2H), 6.95 (dd, *J* = 8.3, 2.1 Hz, 1H), 6.62 (d, *J* = 8.4 Hz, 1H), 6.55 (s, 1H), 4.46 (d, *J* = 6.8 Hz, 1H), 4.27 – 4.19 (m, 2H), 3.77 (td, *J* = 6.8, 3.4 Hz, 1H), 3.38 (dd, *J* = 11.7, 7.3 Hz, 1H),3.31 (dd, *J* = 11.7, 3.4 Hz, 1H), 2.91 (s, 3H), 2.13 (s, 3H), 1.29 (t, *J* = 7.2 Hz, 3H).

^{13}C NMR (101 MHz, Chloroform-*d*) δ 194.22, 161.07, 143.86, 143.59, 131.56, 130.85, 130.60, 128.48, 126.85, 122.78, 120.63, 112.04, 62.48, 49.86, 49.67, 43.97, 39.69, 20.25, 13.90.

HRMS (ESI): m/z Calcd. for [$C_{21}H_{23}BrNO_3$, M+H]$^{+}$:416.0855; Found: 416.0856.

ethyl 2-(*trans*-1,6-dimethyl-4-(naphthalen-2-yl)-1,2,3,4-tetrahydroquinolin-3-yl)-2-oxoacetate (3ac):

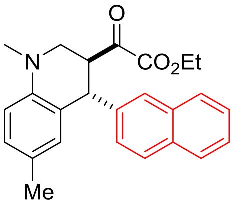
3ac

Flash column chromatography (eluent: EtOAc/Hexanes = 1/10, v/v) to afford **3ac** as a yellow oil, 54.1 mg, 70% yield, > 20:1 dr. Rf = 0.43 (1:5 EtOAc/Hexanes).

^{1}H NMR (400 MHz, Chloroform-*d*) δ 7.84 (dt, *J* = 13.8, 5.1 Hz, 3H), 7.59 (s, 1H), 7.50-7.45 (m, 2H), 7.31 (dd, *J* = 8.5, 1.7 Hz, 1H), 7.00 (ddd, *J* = 8.4, 2.1, 0.9 Hz, 1H), 6.69 (d, *J* = 8.3 Hz, 1H), 6.64 (s, 1H), 4.67 (d, *J* = 7.3 Hz, 1H), 4.14– 3.98 (m, 3H), 3.49 – 3.38 (m, 2H), 2.98 (s, 3H), 2.12 (s, 3H), 1.16 (t, *J* = 7.1 Hz, 3H).

^{13}C NMR (101 MHz, Chloroform-*d*) δ 194.76, 161.10, 144.02, 141.53, 133.20, 132.37, 130.76, 128.36, 128.34, 128.30, 127.75, 127.53, 126.79, 126.70, 126.07, 125.74, 123.46, 111.96, 62.28, 50.24, 49.48, 45.15, 39.73, 20.22, 13.68.

HRMS (ESI): m/z Calcd. for [$C_{25}H_{26}NO_3$, M+H]$^{+}$:388.1907; Found: 388.1907.

ethyl 2-(*trans*-1,6-dimethyl-4-(thiophen-2-yl)-1,2,3,4-tetrahydroquinolin-3-yl)-2-oxoacetate (3ad):

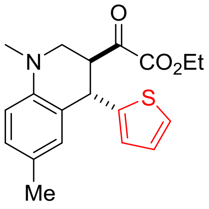

3ad

Flash column chromatography (eluent: EtOAc/Hexanes = 1/10, v/v) to afford **3ad** as a yellow oil, 40.4 mg, 59% yield, > 20:1 dr. Rf = 0.53 (1:5 EtOAc/Hexanes).

^{1}H NMR (400 MHz, Chloroform-*d*) δ 7.19 (dd, *J* = 5.2, 1.3 Hz, 1H), 6.96 (d, *J* = 8.3 Hz, 1H), 6.92 (ddd, *J* = 5.1, 3.4, 1.6 Hz, 1H), 6.83 (s, 1H), 6.76 (d, *J* = 2.4 Hz, 1H), 6.60 (d, *J* = 8.4 Hz, 1H), 4.79 (d, *J* = 5.1 Hz, 1H), 4.32 – 4.24 (m, 2H), 3.80 – 3.76 m, 1H), 3.49 (dd, *J* = 11.9, 5.8 Hz, 1H), 3.45 – 3.38 (m, 1H), 2.89 (s, 3H), 2.18 (s, 3H), 1.33 (t, *J* = 7.1, 3H).

^{13}C NMR (101 MHz, Chloroform-*d*) δ 193.79, 161.23, 148.70, 143.22, 130.77, 128.63, 126.79, 126.56, 126.23, 124.43, 122.68, 112.06, 62.37, 50.37, 49.52, 39.58, 39.08, 20.28, 13.95.

HRMS (ESI): m/z Calcd. for [$C_{19}H_{22}NO_3S$, M+H]$^+$:344.1314; Found: 344.1314.

methyl 2-(*trans*-1,6-dimethyl-4-phenyl-1,2,3,4-tetrahydroquinolin-3-yl)-2-oxoace -tate (3ae):

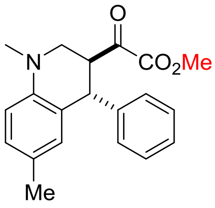

3ae

Flash column chromatography (eluent: EtOAc/Hexanes = 1/10, v/v) to afford **3ae** as a yellow oil, 45.3 mg, 70% yield, > 20:1 dr. Rf = 0.42 (1:5 EtOAc/Hexanes).

^{1}H NMR (400 MHz, Chloroform-*d*) δ 7.32 – 7.27 (m, 2H), 7.25 – 7.20 (m, 1H), 7.15 – 7.10 (m, 2H), 6.95 (dd, *J* = 8.4, 2.1 Hz, 1H), 6.66 – 6.59 (m, 2H), 4.50 (d, *J* = 6.4 Hz, 1H), 3.81 (td, *J* = 6.6, 3.4 Hz, 1H), 3.76 (s, 3H), 3.41 (dd, *J* = 11.7, 6.7 Hz, 1H), 3.34 (dd, *J* = 11.7, 3.4 Hz, 1H), 2.93 (s, 3H), 2.14 (s, 3H).

^{13}C NMR (101 MHz, Chloroform-*d*) δ 194.16, 161.55, 144.55, 143.93, 130.73, 129.11, 128.44, 128.29, 126.77, 126.69, 123.25, 111.95, 52.80, 49.87, 49.73, 44.45, 39.72, 20.25.

HRMS (ESI): m/z Calcd. for [$C_{20}H_{22}NO_3$, M+H]$^+$:324.1594; Found: 324.1594.

Synthesis of **4a**

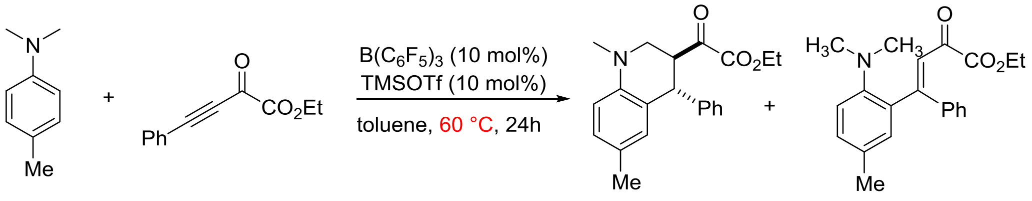

To a Schlenk tube equipped with a dried stir bar was added $B(C_6F_5)_3$ (0.02 mmol), tertiary aniline **1a** (0.24 mmol), alkynone **2a** (0.20 mmol), TMSOTf (0.02 mmol) and toluene (1.0 mL) in the glovebox. The Schlenk tube was sealed with a Teflon screw cap. The reaction mixture was taken outside the glovebox and allowed to stir at 60 ℃ for 24 hours. The crude reaction mixture was concentrated under reduced pressure and directly purified by silica gel chromatography (ethyl acetate:hexanes = 1:10) to afford **4a** in 28% yield.

ethyl 4-(2-(dimethylamino)-5-methylphenyl)-2-oxo-4-phenylbut-3-enoate (4a):

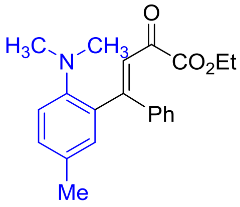

4a

Flash column chromatography (eluent: EtOAc/Hexanes = 1/10, v/v) to afford **4a** as a red solid. m.p.: 132~133 ℃. Rf = 0.52 (1:5 EtOAc/Hexanes).

^{1}H NMR (400 MHz, Chloroform-*d*) δ 7.42 – 7.32 (m, 5H), 7.11 (dd, *J* = 7.5, 2.2 Hz, 1H), 6.88 (d, *J* = 8.2 Hz, 1H), 6.83 – 6.77 (m, 2H), 4.01 (q, *J* = 7.2 Hz, 2H), 2.53 (s, 6H), 2.24 (s, 3H), 1.23 (t, *J* = 7.2 Hz, 3H).

^{13}C NMR (101 MHz, $CDCl_3$) δ 183.57, 162.74, 156.06, 149.12, 140.35, 132.65, 130.69, 130.08, 129.72, 128.34, 128.16, 121.94, 118.28, 61.81, 42.85, 20.44, 13.88.

HRMS (ESI): m/z Calcd. for $[C_{21}H_{24}NO_3, M+H]^+$: 338.1750; Found: 338.1750.

1-(*trans*-1,6-dimethyl-4-phenyl-1,2,3,4-tetrahydroquinolin-3-yl)-2,2,2-trifluoroethan-1-one (6a):

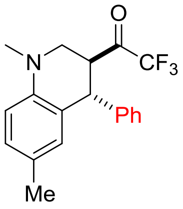

6a

Flash column chromatography (eluent: EtOAc/Hexanes = 1/10, v/v) to afford **6a** as a yellow oil, 53.5 mg, 80% yield, > 20:1 dr. Rf = 0.47 (1:1:20 triethylamine/acetone/Hexanes).

^{1}H NMR (400 MHz, Chloroform-*d*) δ 7.34 – 7.23 (m, 3H), 7.13 – 7.10 (m, 2H), 6.98 (dd, *J* = 8.6, 2.1 Hz, 1H), 6.66 (d, *J* = 8.3 Hz, 1H), 6.61 (s, 1H), 4.53 (d, *J* = 7.2 Hz, 1H), 3.60 (td, *J* = 7.1, 3.7 Hz, 1H), 3.43 – 3.36 (m, 2H), 2.96 (s, 3H), 2.15 (s, 3H).

^{13}C NMR (101 MHz, Chloroform-*d*) δ 191.38 (q, *J* = 34.9 Hz), 143.80, 143.66, 130.72, 128.96, 128.62, 128.43, 127.03, 126.98, 123.11, 115.49 (q, *J* = 293.8 Hz), 112.00, 50.38, 49.18, 44.62, 39.67, 20.26.

^{19}F NMR (377 MHz, Chloroform-*d*) δ -77.90.

HRMS (ESI): m/z Calcd. for $[C_{19}H_{19}F_3NO, M+H]^+$:334.1413; Found: 334.1413.

1-(*trans*-1,6-dimethyl-4-(m-tolyl)-1,2,3,4-tetrahydroquinolin-3-yl)-2,2,2-trifluoroethan-1-one (6b):

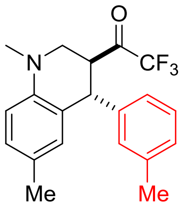

6b

Flash column chromatography (eluent: EtOAc/Hexanes = 1/10, v/v) to afford **6b** as a yellow oil, 43.8mg, 63% yield, > 20:1 dr. Rf = 0.47 (1:1:20 triethylamine/acetone/Hexanes).

^{1}H NMR (400 MHz, Chloroform-*d*) δ 7.19 (t, *J* = 7.6 Hz, 1H), 7.07 (d, *J* = 7.4 Hz, 1H), 6.98 (dd, *J* = 8.4, 2.1 Hz, 1H), 6.94 (s, 1H), 6.89 (d, *J* = 7.7 Hz, 1H), 6.66 (d, *J* = 8.4 Hz, 1H), 6.62 (s, 1H), 4.49 (d, *J* = 7.1 Hz, 1H), 3.59 (td, *J* = 7.0, 3.6 Hz, 1H), 3.43 – 3.34 (m, 2H), 2.96 (s, 3H), 2.33 (s, 3H), 2.15 (s, 3H).

^{13}C NMR (101 MHz, Chloroform-*d*) δ 191.43 (q, *J* = 34.8 Hz), 143.78, 143.63, 138.25, 130.75, 129.54, 128.46, 128.36, 126.97, 127.78, 126.10, 123.21, 115.48 (q, *J* = 294.0 Hz), 111.94, 50.35, 49.15, 44.49, 39.68, 21.38, 20.28.

^{19}F NMR (377 MHz, Chloroform-*d*) δ -77.81.

HRMS (ESI): m/z Calcd. for $[C_{20}H_{21}F_3NO, M+H]^+$:348.1570; Found: 348.1570.

1-(*trans*-4-(4-(tert-butyl)phenyl)-1,6-dimethyl-1,2,3,4-tetrahydroquinolin-3-yl)-2,2,2-trifluoroethan-1-one (6c):

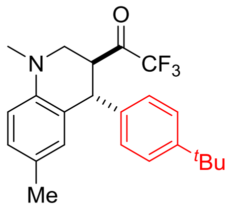

6c

Flash column chromatography (eluent: EtOAc/Hexanes = 1/10, v/v) to afford **6c** as a yellow oil, 60.1 mg, 77% yield, > 20:1 dr. Rf = 0.45 (1:1:20 triethylamine/acetone/Hexanes).

^{1}H NMR (400 MHz, Chloroform-*d*) δ 7.31 (dd, *J* = 8.4, 1.8 Hz, 2H), 7.01 (dd, *J* = 8.4, 1.9 Hz, 2H), 6.97 (d, *J* = 8.5 Hz, 1H), 6.68 – 6.64 (m, 2H), 4.51 (d, *J* = 6.1 Hz, 1H), 3.55 – 3.51 (m, 1H), 3.41 – 3.37 (m, 2H), 2.95 (d, *J* = 1.4 Hz, 3H), 2.16 (s, 3H), 1.32 (d, *J* = 1.8 Hz, 9H).

^{13}C NMR (101 MHz, Chloroform-*d*) δ 191.36 (q, *J* = 34.7 Hz), 149.79, 143.66, 140.97, 130.82, 128.50, 128.38, 126.92, 125.45, 122.89, 115.57 (q, *J* = 294.0 Hz), 111.89, 49.76, 49.15, 43.77, 39.60, 34.41, 31.30, 20.28.

^{19}F NMR (377 MHz, Chloroform-*d*) δ -77.41.

HRMS (ESI): m/z Calcd. for $[C_{23}H_{27}F_3NO, M+H]^+$:390.2039; Found: 390.2039.

1-(*trans*-4-([1,1'-biphenyl]-4-yl)-1,6-dimethyl-1,2,3,4-tetrahydroquinolin-3-yl)-2,2,2-trifluoroethan-1-one (6d):

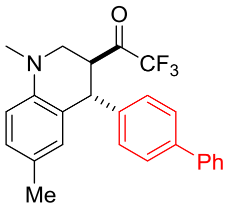

6d

Flash column chromatography (eluent: EtOAc/Hexanes = 1/10, v/v) to afford **6d** as a yellow oil, 64.8 mg, 79% yield, > 20:1 dr. Rf = 0.37 (1:1:20 triethylamine/acetone/Hexanes).

^{1}H NMR (400 MHz, Chloroform-*d*) δ 7.63 – 7.61 (m, 2H), 7.56 (d, *J* = 8.3 Hz, 2H), 7.46 (t, *J* = 7.6 Hz, 2H), 7.39 – 7.35 (m, 1H), 7.20 (d, *J* = 6.3 Hz, 2H), 7.01 (dd, *J* = 8.4, 2.1 Hz, 1H), 6.71 – 6.68 (m, 2H), 4.61 (d, *J* = 7.0 Hz, 1H), 3.64 (td, *J* = 7.0, 3.5 Hz, 1H), 3.45 – 3.40 (m, 2H), 2.99 (s, 3H), 2.18 (s, 3H).

^{13}C NMR (101 MHz, Chloroform-*d*) δ 191.31 (q, *J* = 34.8 Hz), 143.67, 142.96, 140.56, 139.85, 130.76, 129.35, 128.75, 128.50, 127.29, 127.04, 126.98, 122.93, 120.27 – 115.53 (q, *J* = 294.0 Hz), 112.03, 50.22, 49.15, 44.14, 39.65, 20.27.

^{19}F NMR (377 MHz, Chloroform-*d*) δ -77.61.

HRMS (ESI): m/z Calcd. for $[C_{25}H_{23}F_3NO, M+H]^+$:410.1726; Found: 410.1726.

2,2,2-trifluoro-1-(*trans*-4-(4-methoxyphenyl)-1,6-dimethyl-1,2,3,4-tetrahydroquinolin-3-yl)ethan-1-one (6e):

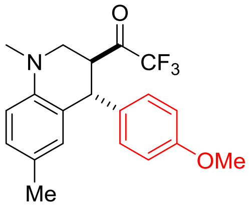

6e

Flash column chromatography (eluent: EtOAc/Hexanes = 1/10, v/v) to afford **6e** as a yellow oil, 48mg, 66% yield, > 20:1 dr. Rf = 0.35 (1:1:20 triethylamine/acetone/Hexanes).

^{1}H NMR (400 MHz, Chloroform-*d*) δ 7.04 (d, *J* = 8.7 Hz, 2H), 6.97 (d, *J* = 8.3 Hz, 1H), 6.85 (d, *J* = 8.8 Hz, 2H), 6.66 (d, *J* = 8.4 Hz, 1H), 6.62 (s, 1H), 4.48 (d, *J* = 7.5 Hz, 1H), 3.81 (s, 3H), 3.59 (td, *J* = 7.4, 3.7 Hz, 1H), 3.43 – 3.34(m, 2H), 2.96 (s, 3H), 2.15 (s, 3H).

^{13}C NMR (101 MHz, Chloroform-*d*) δ 191.55 (q, *J* = 34.8 Hz), 158.54, 143.58, 135.63, 130.62, 129.92, 128.33, 126.90, 123.57, 115.47 (q, *J* = 294.0 Hz), 113.96, 111.96, 55.15, 50.61, 49.21, 43.99, 39.66, 20.25.

^{19}F NMR (377 MHz, Chloroform-*d*) δ -77.99.

HRMS (ESI): m/z Calcd. for $[C_{20}H_{21}F_3NO_2, M+H]^+$:364.1518; Found: 364.1519.

1-(*trans*-1,6-dimethyl-4-(thiophen-2-yl)-1,2,3,4-tetrahydroquinolin-3-yl)-2,2,2-trifluoroethan-1-one (6f):

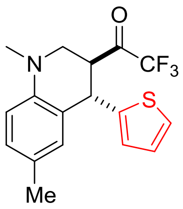

6f

Flash column chromatography (eluent: EtOAc/Hexanes = 1/10, v/v) to afford **6f** as a yellow oil, 33.7mg, 50% yield, > 20:1 dr. Rf = 0.40 (1:1:20 triethylamine/acetone/Hexanes).

^{1}H NMR (400 MHz, Chloroform-*d*) δ 7.23 (d, *J* = 5.2 Hz, 1H), 7.00 (d, *J* = 8.3 Hz, 1H), 6.96 – 6.94 (m, 1H), 6.86 (s, 1H), 6.81 (d, *J* = 3.8 Hz, 1H), 6.65 (d, *J* = 8.4 Hz, 1H), 4.83 (d, *J* = 5.5 Hz, 1H), 3.64 – 3.60 (m, 1H), 3.52 –3.42 (m, 2H), 2.95 (s, 3H), 2.21 (s, 3H).

^{13}C NMR (101 MHz, Chloroform-*d*) δ 190.74 (q, *J* = 34.7 Hz), 147.70, 143.03, 130.70, 128.81, 127.01, 126.64, 126.51, 124.75, 122.43, 121.07 – 115.57 (q, *J* = 294.0 Hz), 112.09, 49.79, 49.75, 39.49, 39.19, 20.28.

^{19}F NMR (377 MHz, Chloroform-*d*) δ -77.08.

HRMS (ESI): m/z Calcd. for $[C_{17}H_{17}F_3NOS, M+H]^+$:340.0977; Found: 340.0977.

4. Mechanistic studies

4.1 Deuterium-labelling experiment

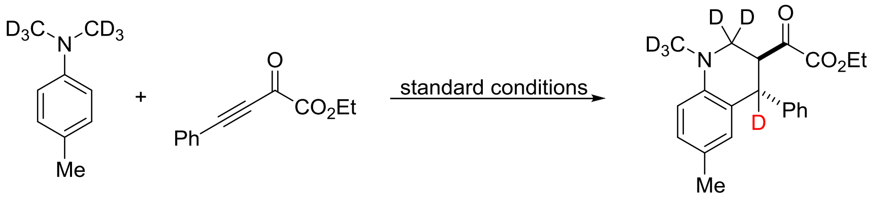

1a-[D6] **2a** **3a-[D6]**, 56% **yield**

To a Schlenk tube equipped with a dried stir bar was added $B(C_6F_5)_3$ (0.02 mmol), **1a-[D6]** (0.24 mmol), alkynone **2a** (0.20 mmol), TMSOTf (0.02 mmol) and toluene (1.0 mL) in the glovebox. The Schlenk tube was sealed with a Teflon screw cap. The reaction mixture was taken outside the glovebox and allowed to stir at 80 ℃ for 24 hours. The crude reaction mixture was concentrated under reduced pressure and directly purified by silica gel chromatography (ethyl acetate:hexanes = 1:10) to afford **3a-[D6]** in 56% yield.

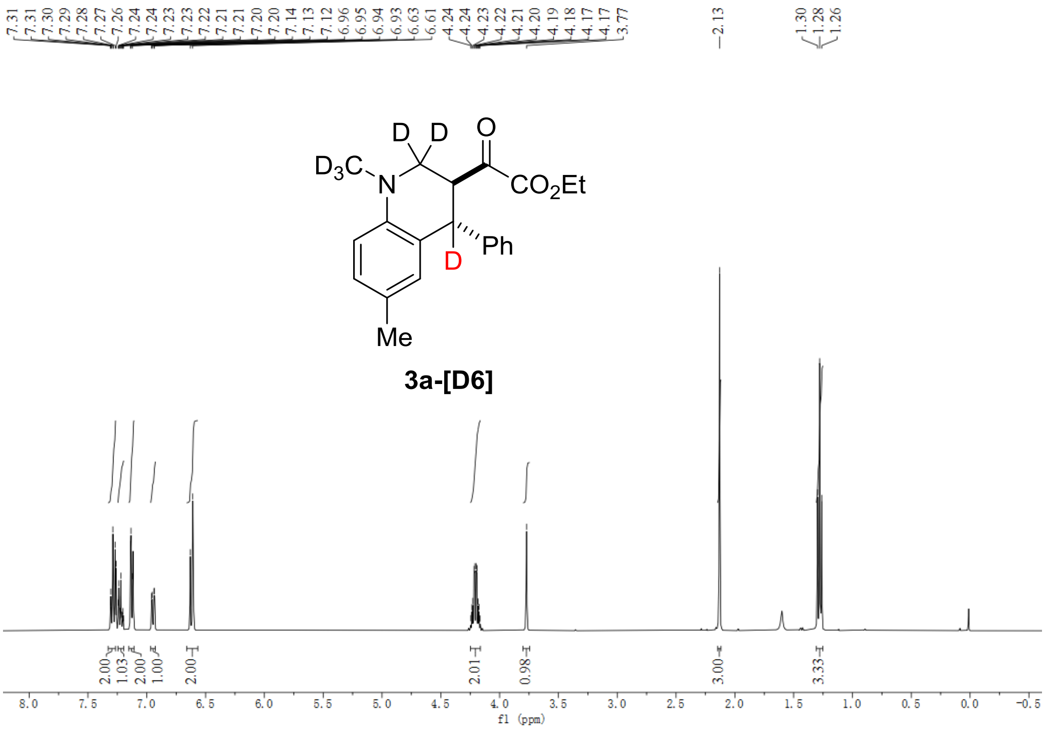

^{1}H NMR (400 MHz, Chloroform-*d*) δ 7.31 – 7.27 (m, 2H), 7.24 – 7.20 (m, 1H), 7.14 – 7.12 (m, 2H), 6.95 (dd, *J* = 8.2, 2.0 Hz, 1H), 6.62 (d, *J* = 8.4 Hz, 2H), 4.24 – 4.17 (m, 2H), 3.77 (s, 1H), 2.13 (s, 3H), 1.28 (t, *J* = 7.1 Hz, 3H).

4.2 Crossover experiment

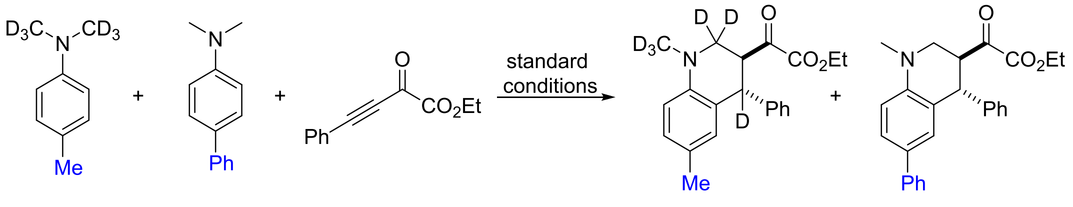

1a-[D6] **1e** **2a** **3a-[D6]**, 63% yield **3e**, 62% yield

To a Schlenk tube equipped with a dried stir bar was added $B(C_6F_5)_3$ (0.02 mmol), **1a-[D6]** (0.12 mmol), **1e** (0.12 mmol), alkynone **2a** (0.20 mmol), TMSOTf (0.02 mmol) and toluene (1.0 mL) in the glovebox. The Schlenk tube was sealed with a Teflon screw cap. The reaction mixture was taken outside the glovebox and allowed to stir at 80 ℃ for 24 hours. The crude reaction mixture was concentrated under reduced pressure and directly purified by silica gel chromatography (ethyl acetate:hexanes = 1:10) to afford **3a-[D6]** and **3e** in 63 and 62% yields, respectively.

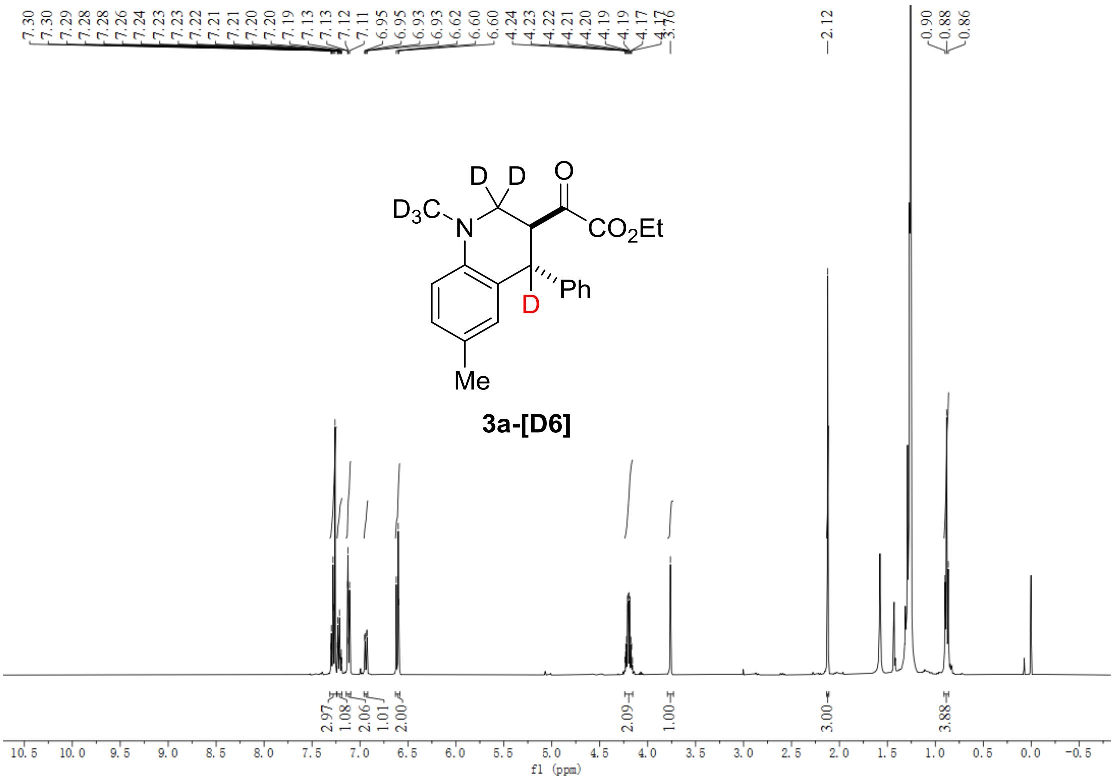

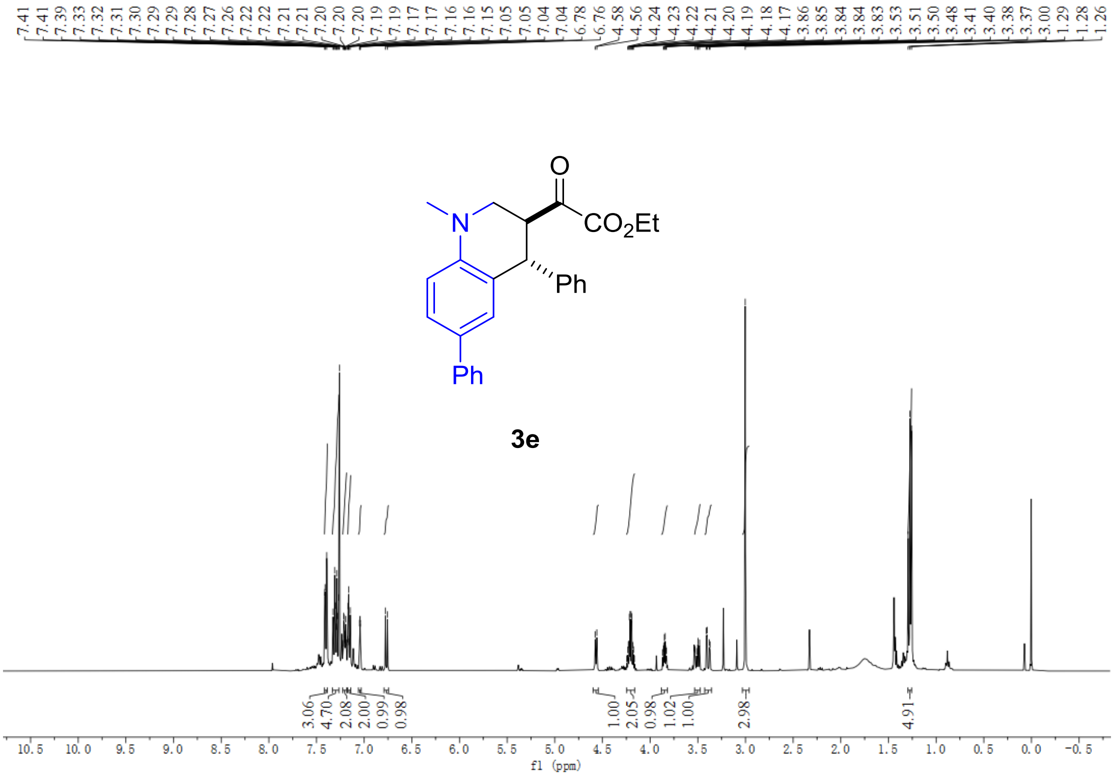

4.3 Kinetic isotope effect

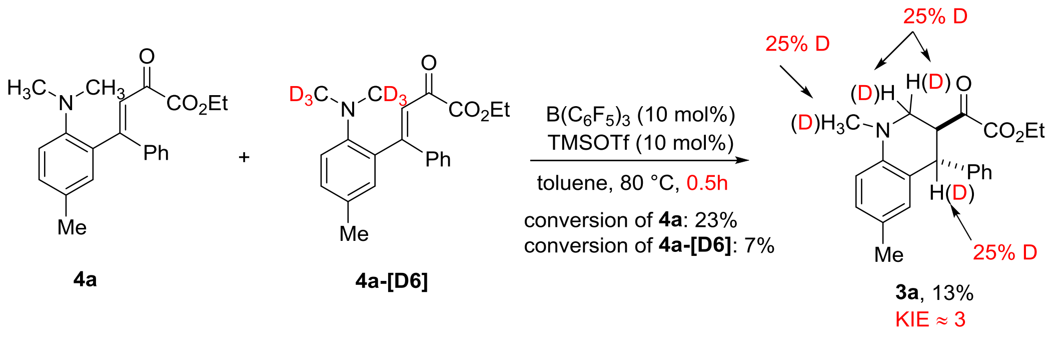

To a Schlenk tube equipped with a dried stir bar was added $B(C_6F_5)_3$ (0.02 mmol), **4a** (0.10 mmol), **4a-[D6]** (0.10 mmol), TMSOTf (0.02 mmol) and toluene (1.0 mL) in the glovebox. The Schlenk tube was sealed with a Teflon screw cap. The reaction mixture was taken outside the glovebox and allowed to stir at 80 ℃ for 0.5 hours. The crude reaction mixture was concentrated under reduced pressure. The conversion of **4a** and **4a-[D6]** were determined by ^{1}H NMR using 1,3,5-trimethoxybenzene as the internal standard. Then the mixture was purified by silica gel chromatography (ethyl acetate:hexanes = 1:10) to afford **3a** in 13% yield. For the hydrogen on the α carbon

of amino moiety and the hydrogen on the benzyl carbon, the ratio of H to D were 75:25.

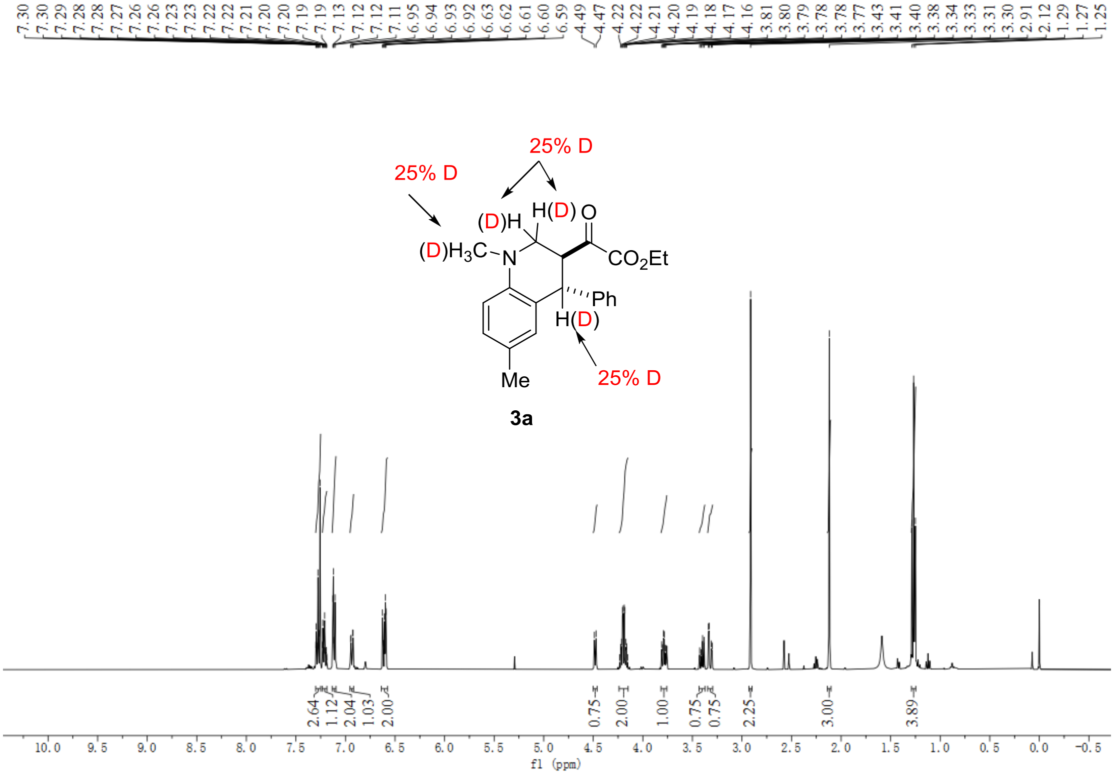

4.4 Role of TMSOTf

Additive TMSOTf was found to be able to improve the reaction efficiency. We have tried to use other acid as catalyst or additive, such as Lambert salt or Brookhart's acid. However, they produced no desired product when used alone. When they were used in combination with $B(C_6F_5)_3$, diminished yields of **3a** and **4a** were observed.

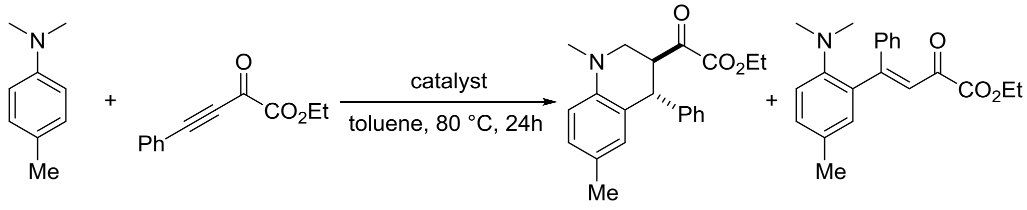

1a **2a** **3a** **4a**

Catalyst	yield of **3a** (%)	yield of **4a** (%)
10 mol% **$[Et_3Si(toluene)B(C_6F_5)_4]$**	ND	ND
10 mol% **$B(C_6F_5)_3$** + 10 mol% **$[Et_3Si(toluene)B(C_6F_5)_4]$**	45	22
10 mol% **$[H(OEt_2)_2]^+[(3,5\text{-}(CF_3)_2C_6H_3)B]^-$**	ND	3
10 mol% **$B(C_6F_5)_3$** + 10 mol% **$[H(OEt_2)_2]^+[(3,5\text{-}(CF_3)_2C_6H_3)B]^-$**	6	26

We surmised that TMSOTf could coordinate with $B(C_6F_5)_3$ to increase the Lewis acidity (**Int S1**). However, **^{19}F NMR** measurement of 1:1 ratio of TMSOTf and $B(C_6F_5)_3$ showed no obvious change in chemical shift and implied this hypothesis is likely not operative. Alternatively, we hypothesized that TMSOTf may coordinate with substrate **2a** (e.g., through the ester moiety) to further increase its electrophilicity (**Int S2**).

a) **^{19}F NMR** of TMSOTf, $B(C_6F_5)_3$ and both

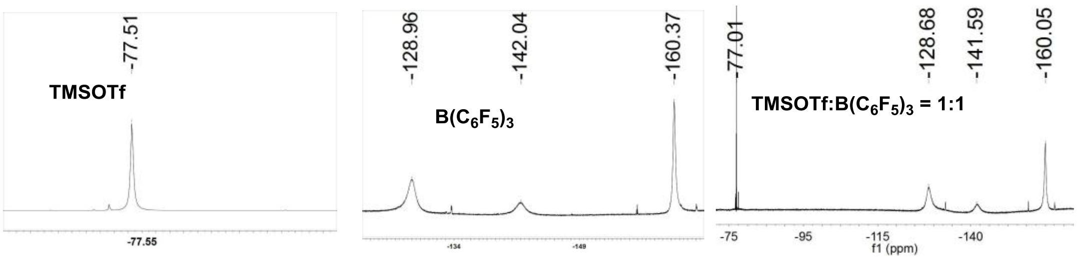

b) Two hypothesis of the effect of TMSOTf (Lewis acid activation of Lewis acid or substrate)

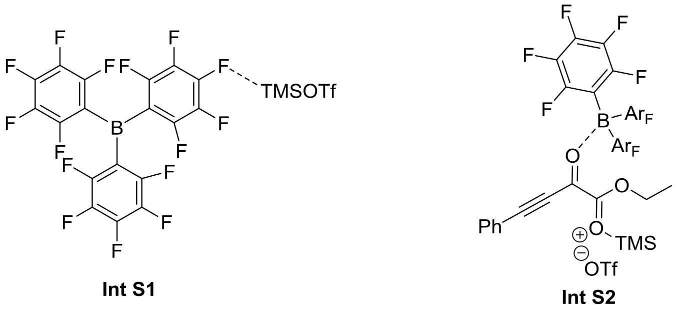

Int S1 **Int S2**

5. X-Ray crystallography data

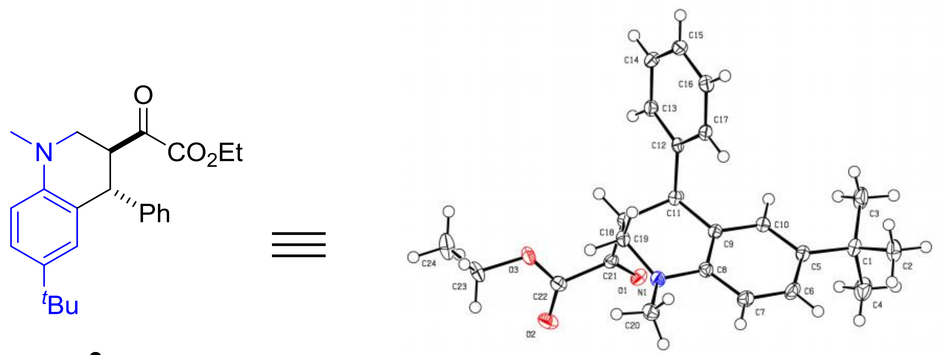

3c

Thermal ellipsoids are drawn on 50% probability level

The single-crystal diffraction data were collected on a Rigaku XtaLAB synergy four-circle diffractometer with Cu-Kα radiation (λ=1.54184Å), with the CrysAlisPro software (version 1.171.39.34b) for data reduction and analysis. The crystal was kept at 100 K during data collection. Using Olex2, the structure was solved with the ShelXT structure solution program using Intrinsic Phasing and refined with the ShelXL refinement package using Least Squares minimisation. All non-hydrogen atoms were refined with anisotropic displacement parameters. All hydrogen atoms were generated geometrically.

Table S1. Crystal data and structure refinement for **3c** (CCDC 2114097)

Empirical formula	$C_{24}H_{29}NO_3$
Formula weight	379.48
Temperature / K	100.01(10)
Crystal system	Monoclinic
Space group	C2/c
a / Å	32.9756(4)
b / Å	9.21260(10)
c / Å	13.7861(2)
α / °	90
β / °	101.2320(10)
γ / °	90
V / Å^3	4107.88(9)
Z	8
F(000)	1632.0
D_c./ g cm^{-3}	1.227
μ / mm^{-1}	0.635

Reflns coll.	11622
Independent reflections	4002
R_{int}	0.0196
$^{a}R_1$ [I≥2σ(I)]	0.0335
$^{b}wR_2$ (all data)	0.0884
GOF	1.035

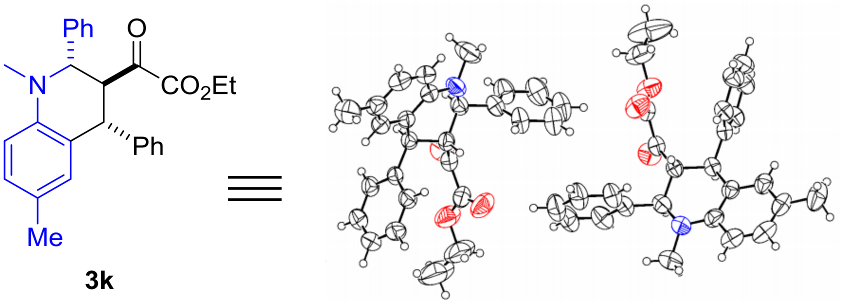

Thermal ellipsoids are drawn on 50% probability level

The single-crystal diffraction data were collected on a Rigaku XtaLAB synergy four-circle diffractometer with Cu-Kα radiation (λ=1.54184Å), with the CrysAlisPro software (version 1.171.39.34b) for data reduction and analysis. The crystal was kept at 100 K during data collection. Using Olex2, the structure was solved with the ShelXT structure solution program using Intrinsic Phasing and refined with the ShelXL refinement package using Least Squares minimisation. All non-hydrogen atoms were refined with anisotropic displacement parameters. All hydrogen atoms were generated geometrically.

Table S2. Crystal data and structure refinement for **3k** (CCDC 2114110)

Empirical formula	$C_{27}H_{27}NO_3$
Formula weight	413.49
Temperature / K	293(2)
Crystal system	Monoclinic
Space group	Cc
a / Å	5.76260(10)
b / Å	21.7654(3)
c / Å	36.0320(5)
α / °	90

β / °	91.8450(10)
γ / °	90
V / Å^3	4516.98(12)
Z	8
F(000)	1760.0
D_c./ g cm^{-3}	1.216
μ / mm^{-1}	0.625
Reflns coll.	52460
Independent reflections	8780
R_{int}	0.0630
$^aR_1$ [I≥2σ(I)]	0.0460
$^bwR_2$ (all data)	0.1271
GOF	1.034

6. References

(1) (a) Hemming, D. S.; Talbot, E. P.; Steel, P. G. A mild copper catalyzed method for the selective deprotection of aryl allyl ethers. *Tetrahedron Lett.* **2017**, *58*, 17-20. (b) Mandal, T.; Das, S.; De Sarkar, S. Nickel(II) Tetraphenylporphyrin as an Efficient Photocatalyst Featuring Visible Light Promoted Dual Redox Activities. *Adv. Syn. & Catal.* **2019**, *361*, 3200-3209. (c) Shi, R.; Lu, L.; Zhang, H.; Chen, B.; Sha,Y.; Liu, C.; Lei, A. Palladium/copper-catalyzed oxidative C-H alkenylation/*N*-dealkylative carbonylation of tertiary anilines. *Angew. Chem. Int. Ed.* **2013**, *52*, 10582-10585. (d) Xu, G. Q.; Xu, J. T.; Feng, Z. T.; Liang, H.; Wang, Z. Y.; Qin, Y.; Xu, P. F. Dual C(sp(3))-H Bond Functionalization of *N*-Heterocycles through Sequential Visible-Light Photocatalyzed Dehydrogenation/[2+2] Cycloaddition Reactions. *Angew. Chem. Int. Ed.* **2018**, *57*, 5110-5114.

(2) Trost, B. M.; Hung, C. J.; Scharf, M. J., Direct Catalytic Asymmetric Vinylogous Additions of α,β- and β,γ-Butenolides to Polyfluorinated Alkynyl Ketimines. *Angew. Chem. Int. Ed.* **2018**, *57*, 11408-11412.

(3) Yang,J.; Wang, Z.;He, Z.; Li, G.; Hong, L.; Sun,W.; Wang, R.,Organocatalytic Enantioselective Synthesis of Tetrasubstituted α-Amino Allenoates by Dearomative γ-Addition of 2,3-Disubstituted Indoles to β,γ-Alkynyl-α-imino Esters. *Angew. Chem. Int. Ed.* **2020**, *59*, 642-647.

7. NMR spectra of products

^{1}H-NMR of **3a** (400 MHz, $CDCl_3$)

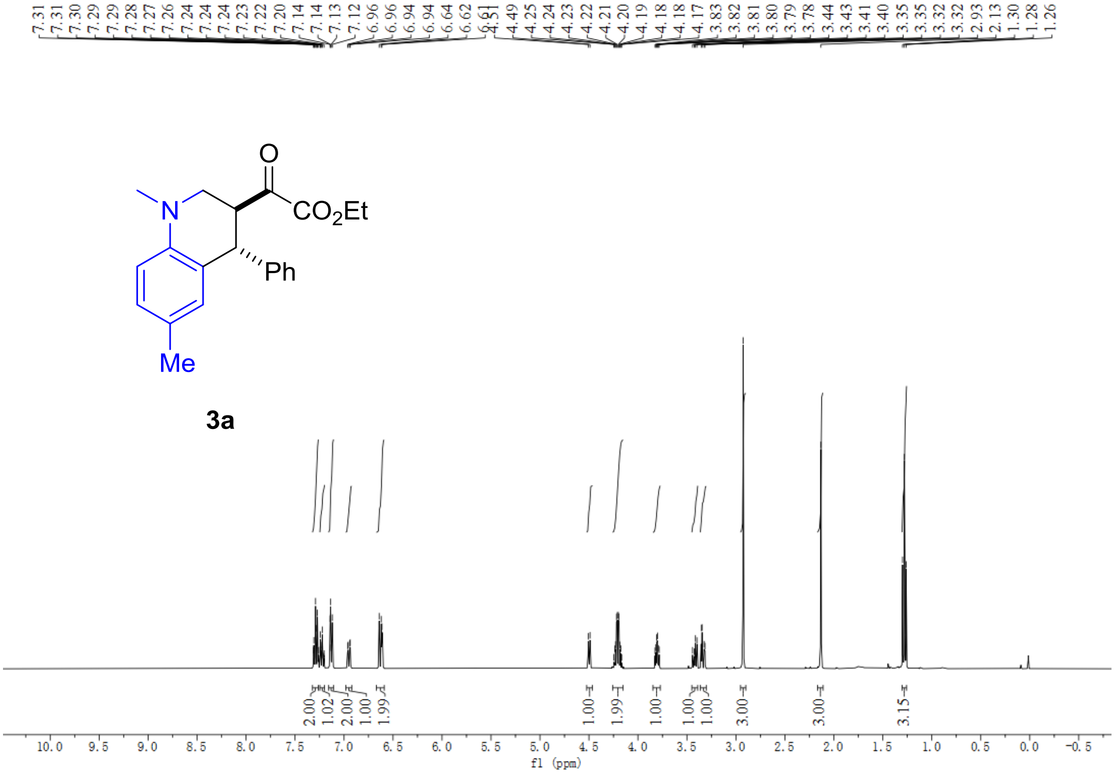

^{13}C-NMR of **3a** (101 MHz, $CDCl_3$)

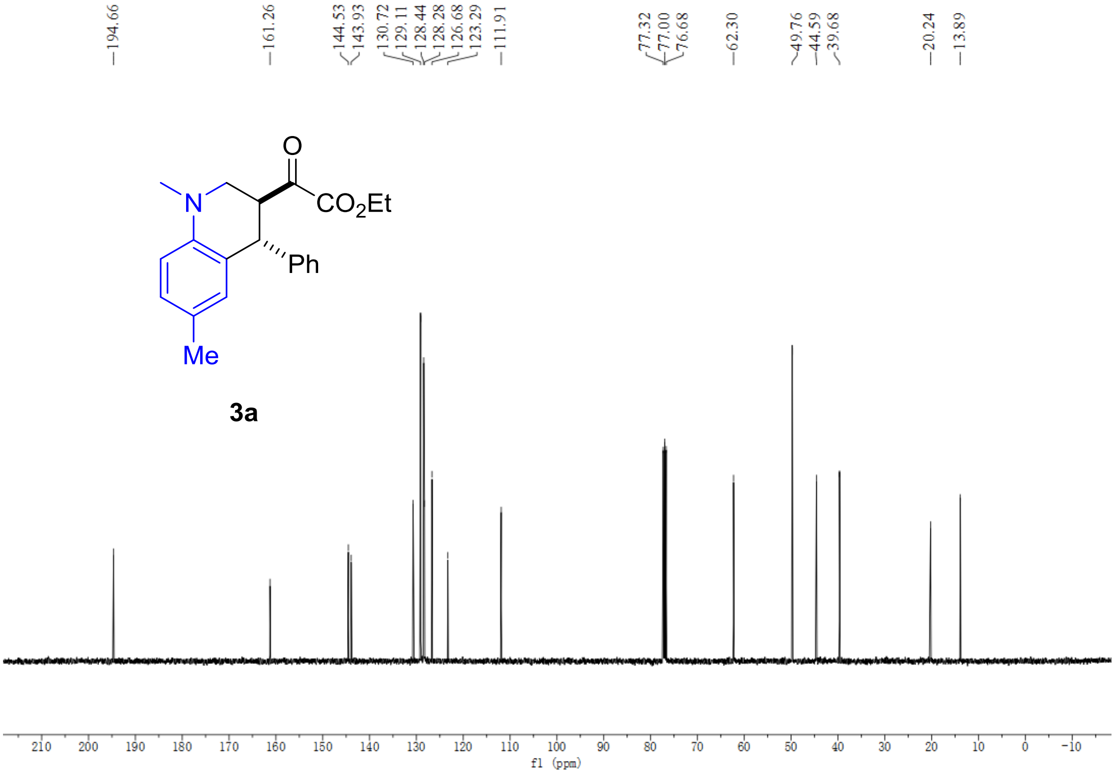

^{1}H-NMR of **3b** (400 MHz, $CDCl_3$)

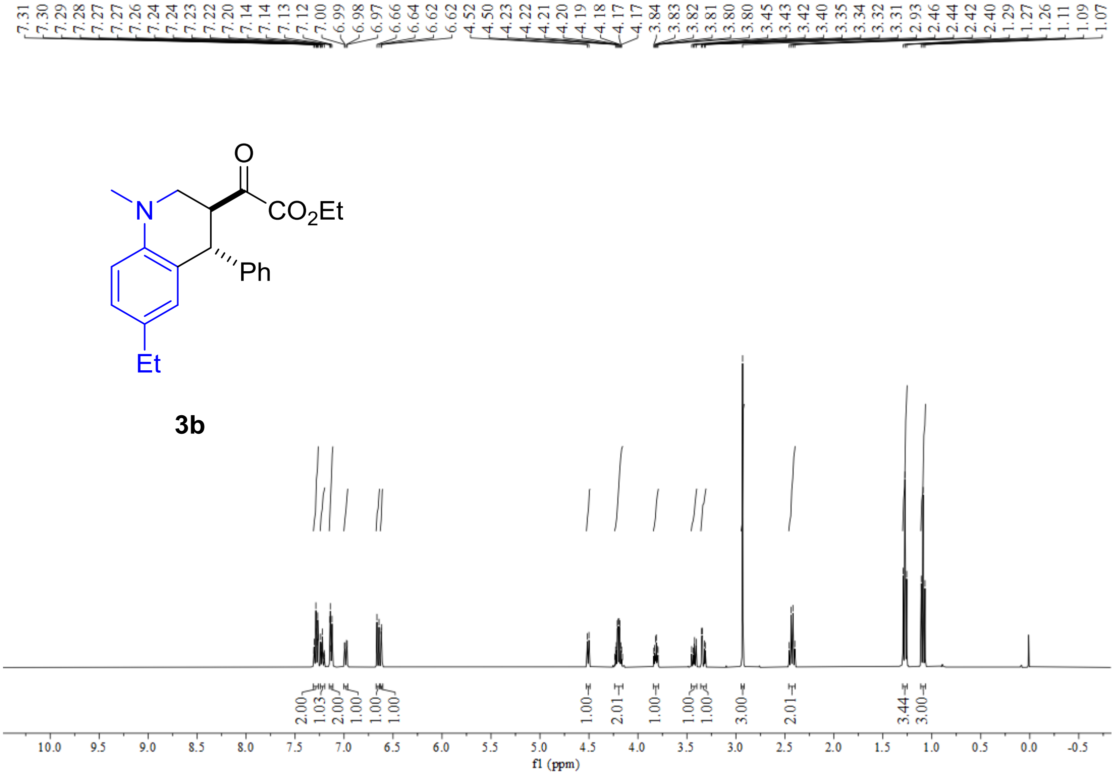

^{13}C-NMR of **3b** (101 MHz, $CDCl_3$)

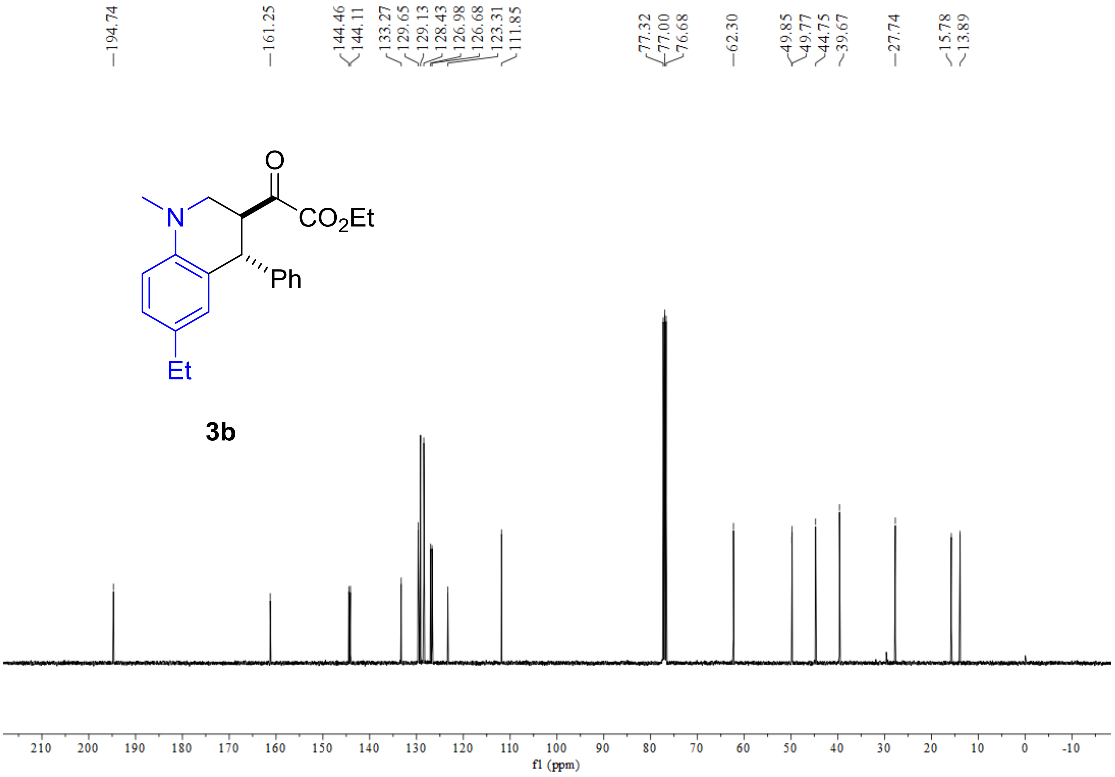

^{1}H-NMR of **3c** (400 MHz, $CDCl_3$)

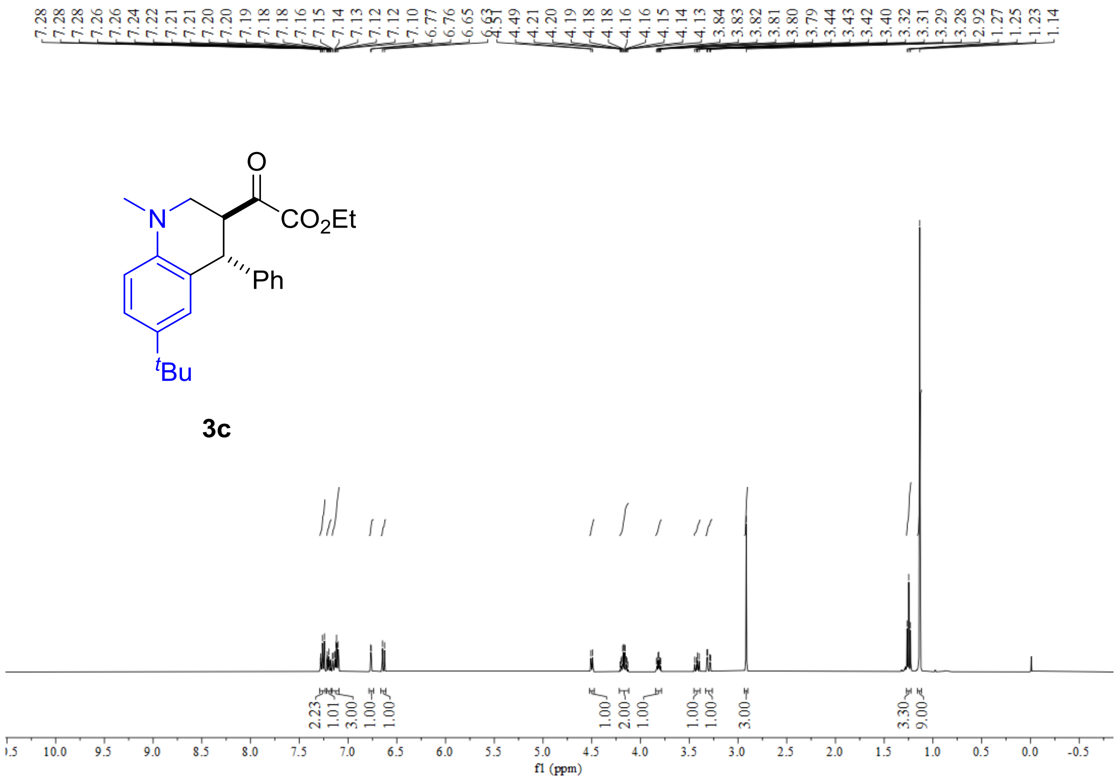

^{13}C-NMR of **3c** (101 MHz, $CDCl_3$)

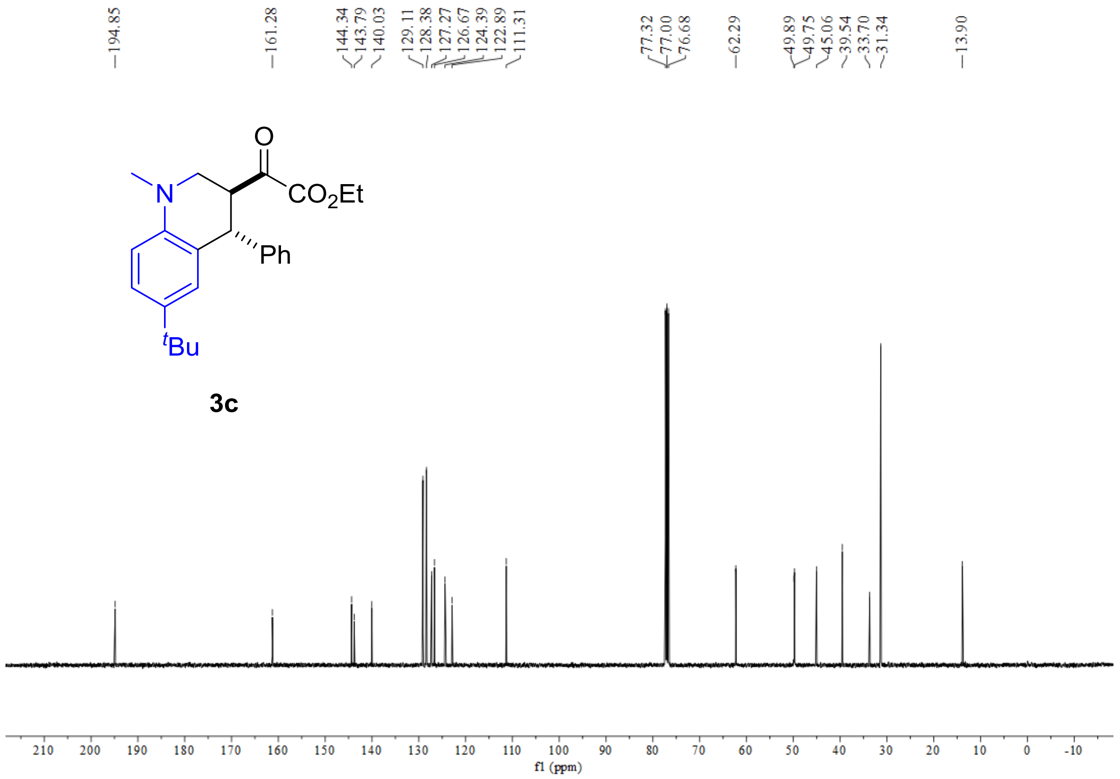

^{1}H-NMR of **3d** (400 MHz, $CDCl_3$)

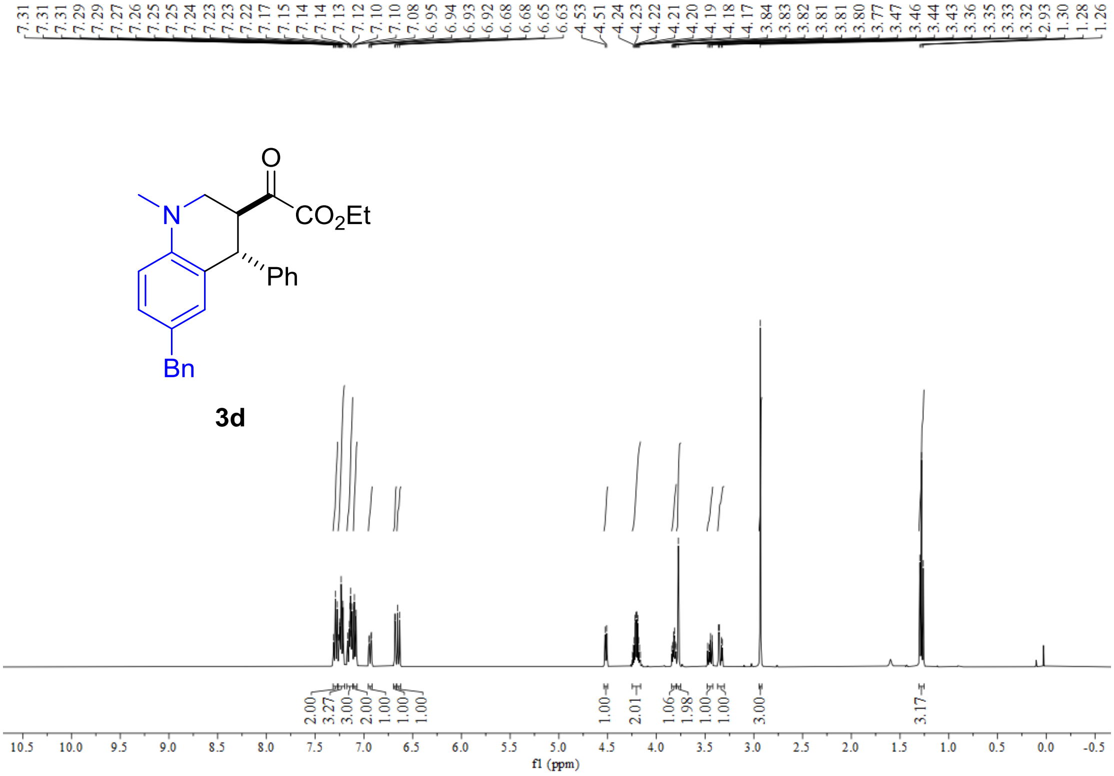

^{13}C-NMR of **3d** (101 MHz, $CDCl_3$)

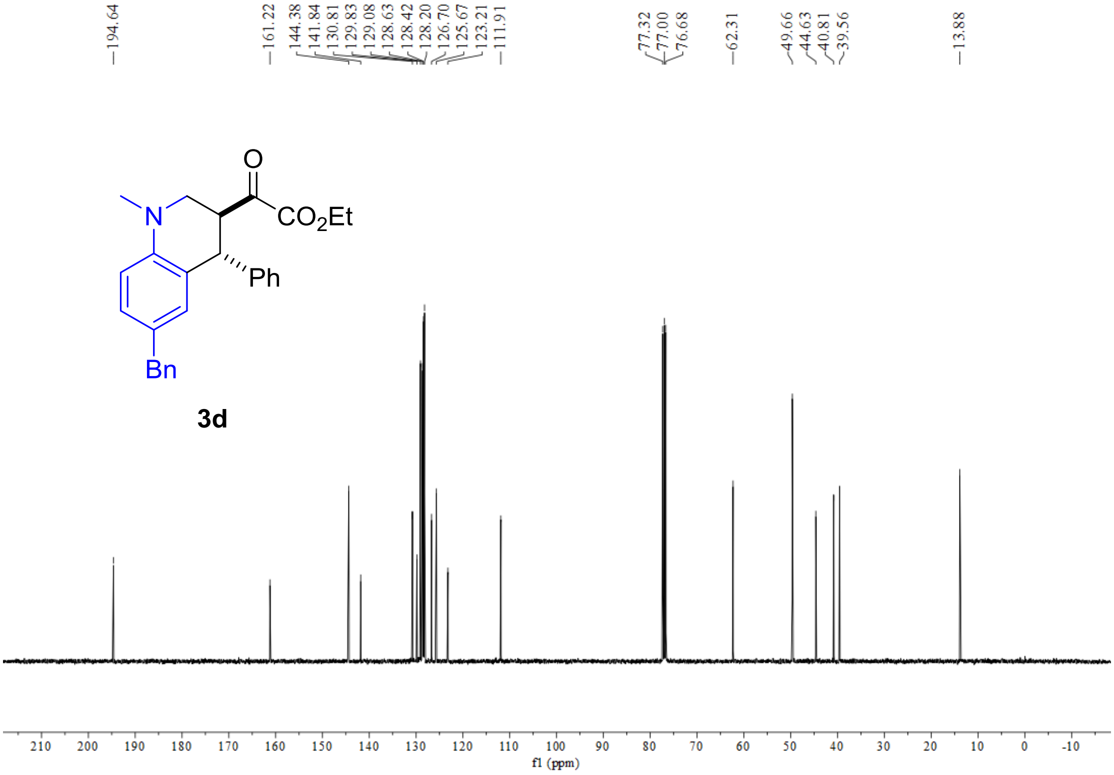

^{1}H-NMR of **3e** (400 MHz, $CDCl_3$)

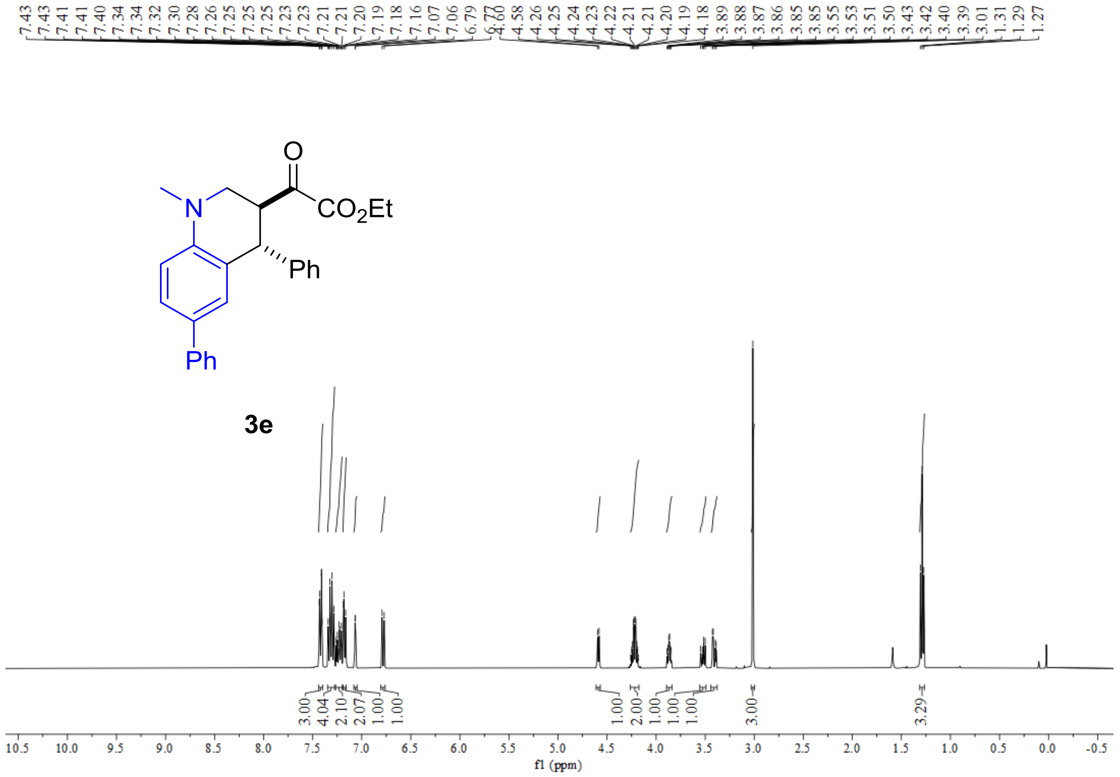

^{13}C-NMR of **3e** (101 MHz, $CDCl_3$)

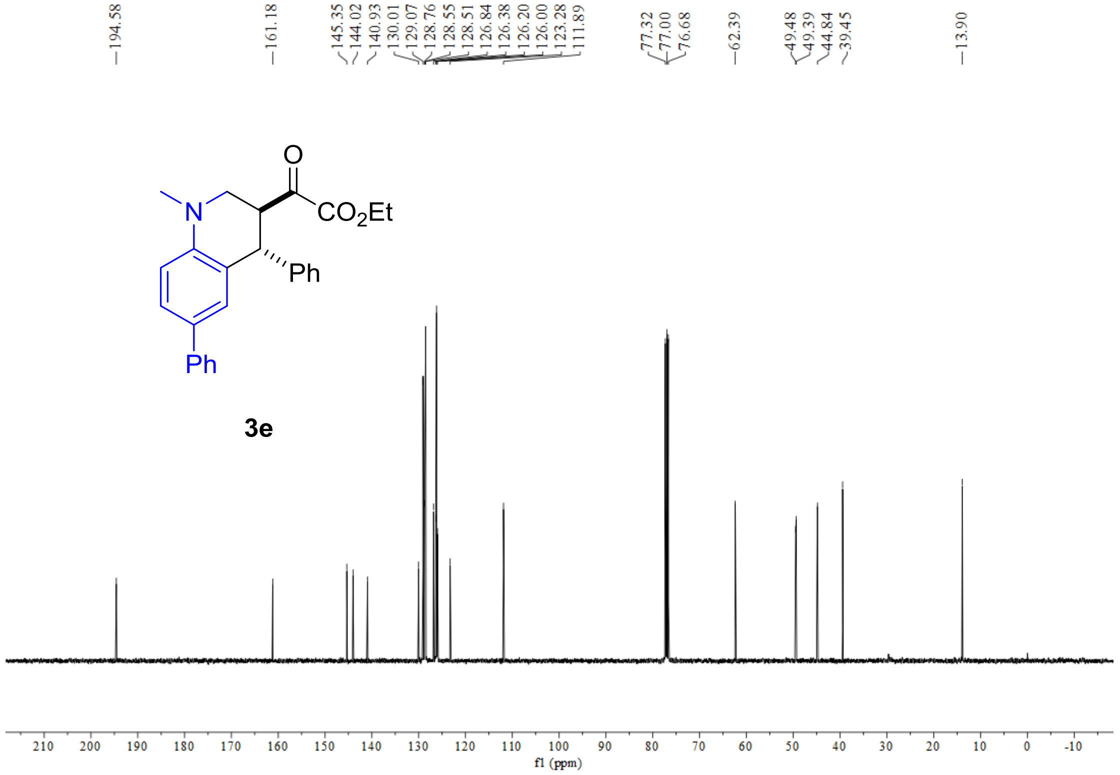

^{1}H-NMR of 3f (400 MHz, $CDCl_3$)

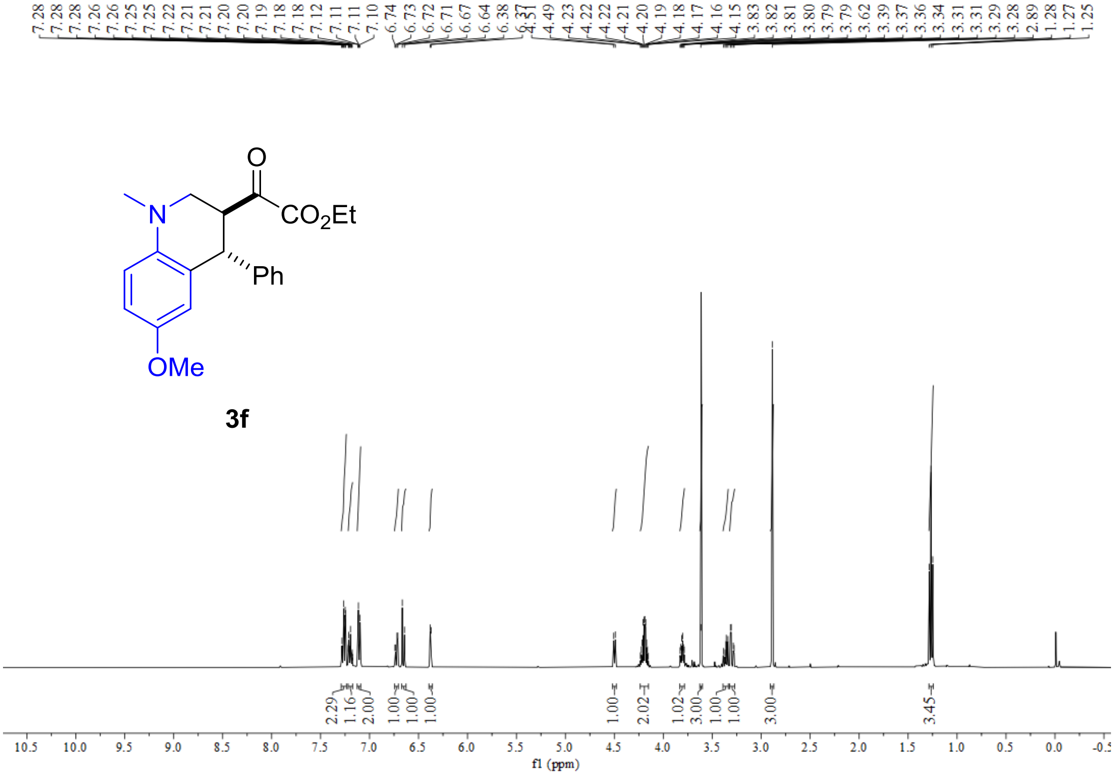

^{13}C-NMR of 3f (101 MHz, $CDCl_3$)

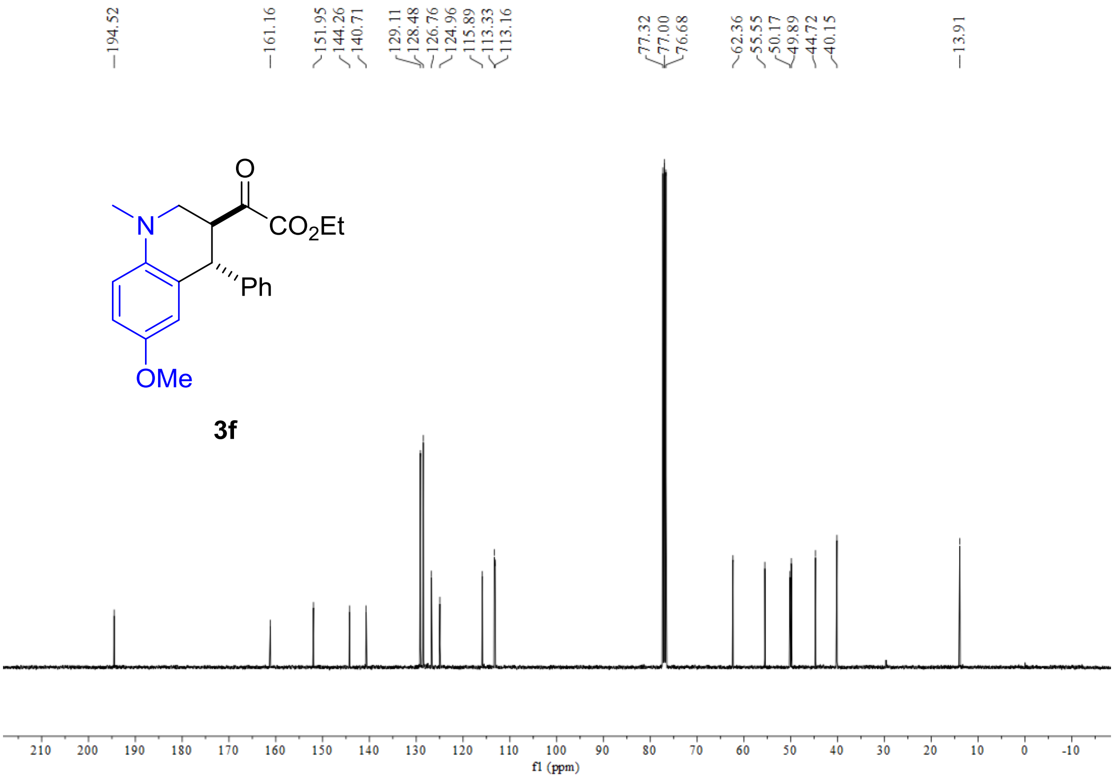

^{1}H-NMR of **3g** (400 MHz, $CDCl_3$)

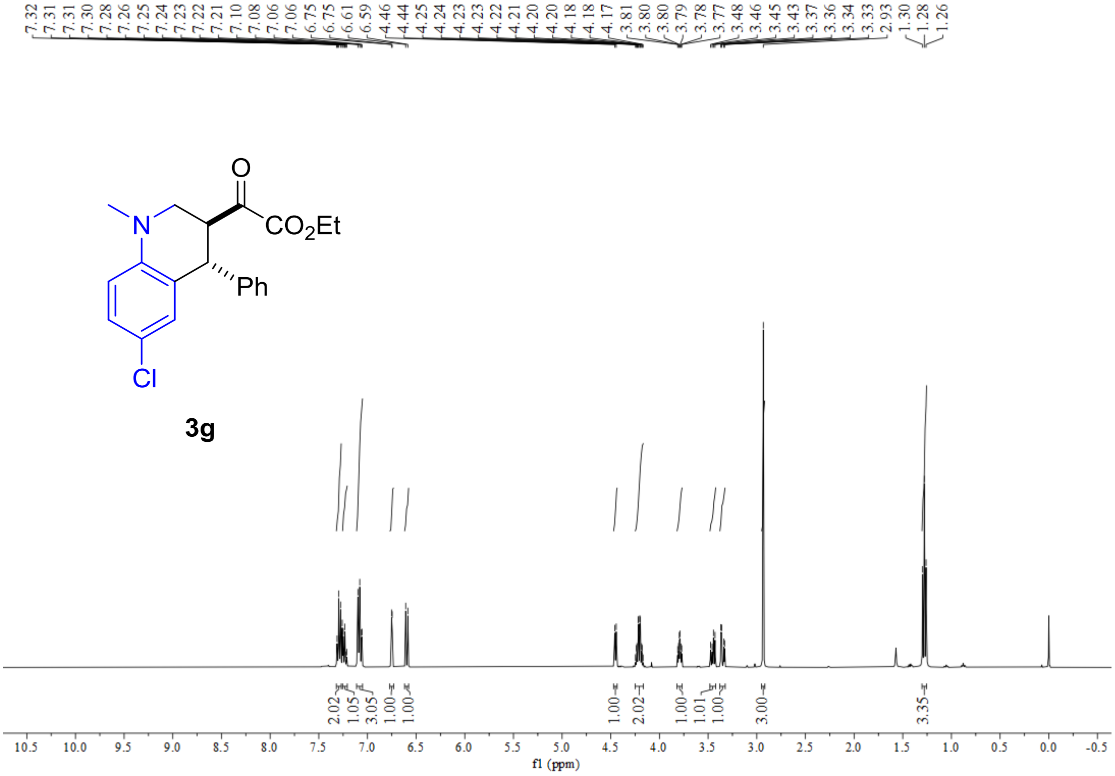

^{13}C-NMR of **3g** (101 MHz, $CDCl_3$)

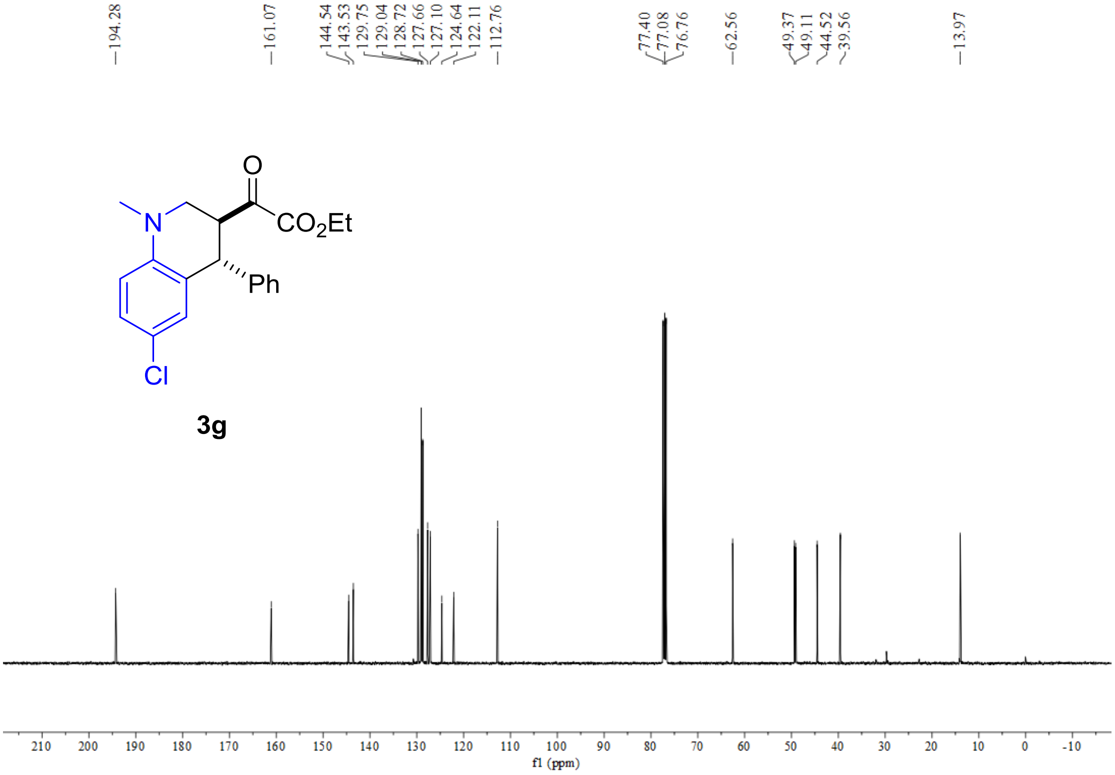

^{1}H-NMR of **3h** (400 MHz, $CDCl_3$)

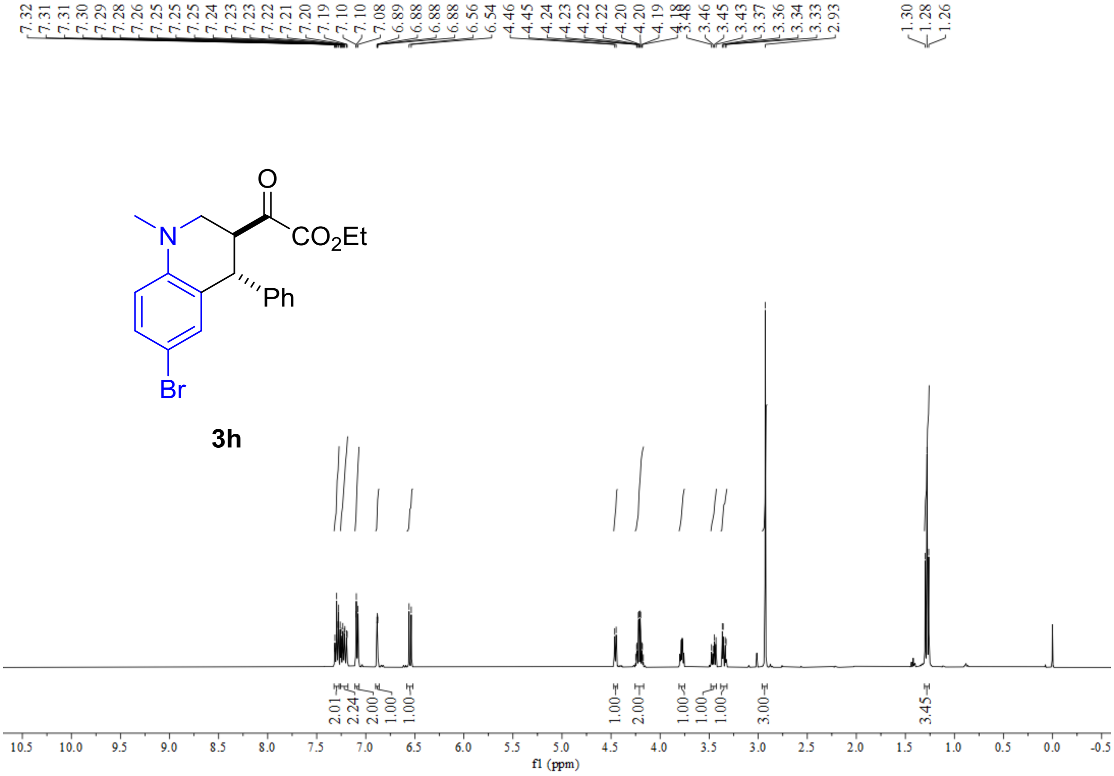

^{13}C-NMR of **3h** (101 MHz, $CDCl_3$)

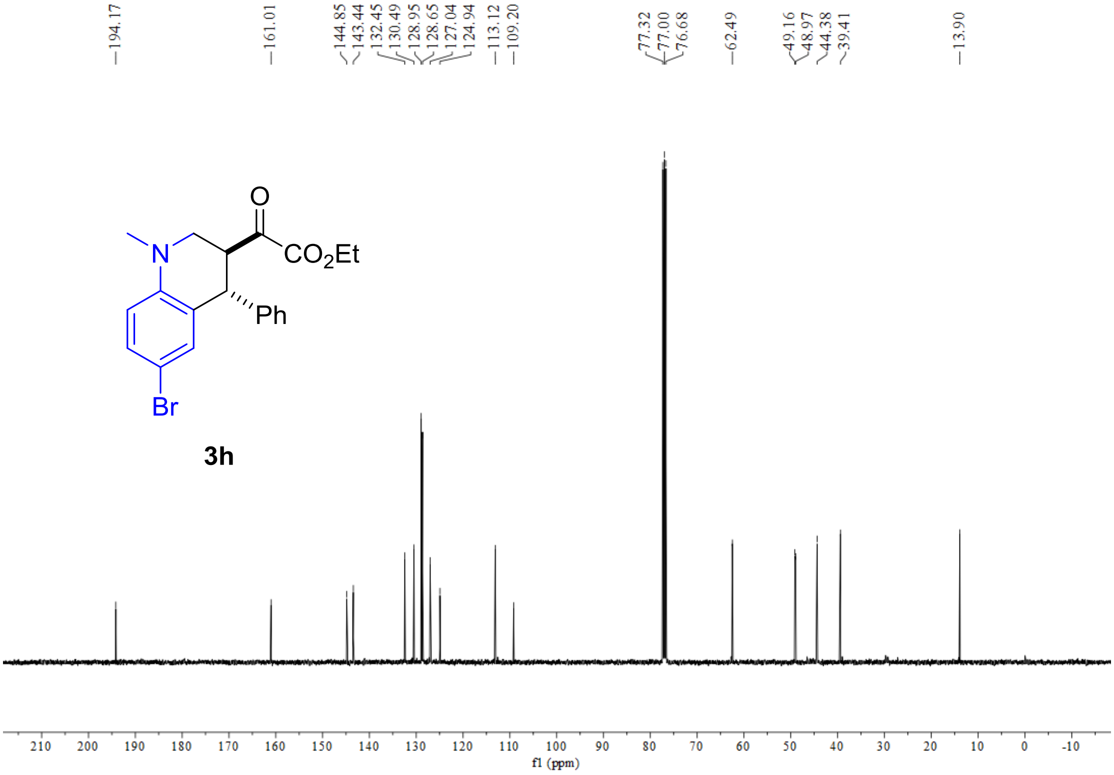

^{1}H-NMR of **3i** (400 MHz, $CDCl_3$)

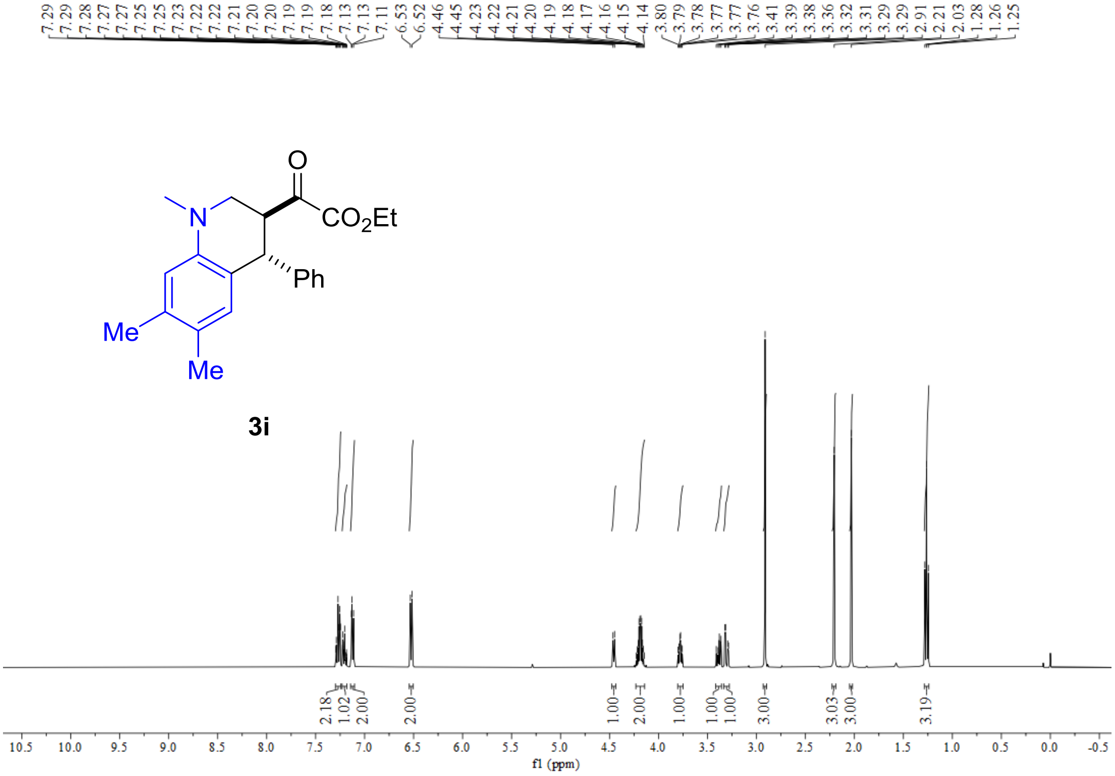

^{13}C-NMR of **3i** (101 MHz, $CDCl_3$)

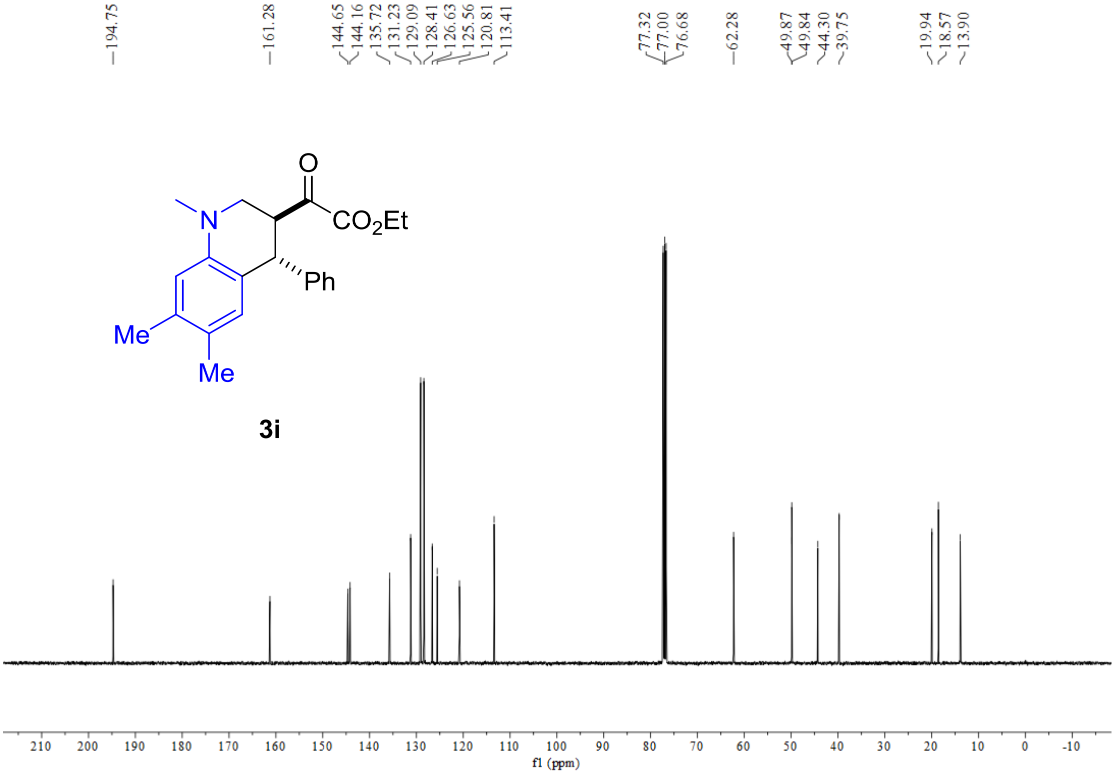

^{1}H-NMR of **3j** (400 MHz, $CDCl_3$)

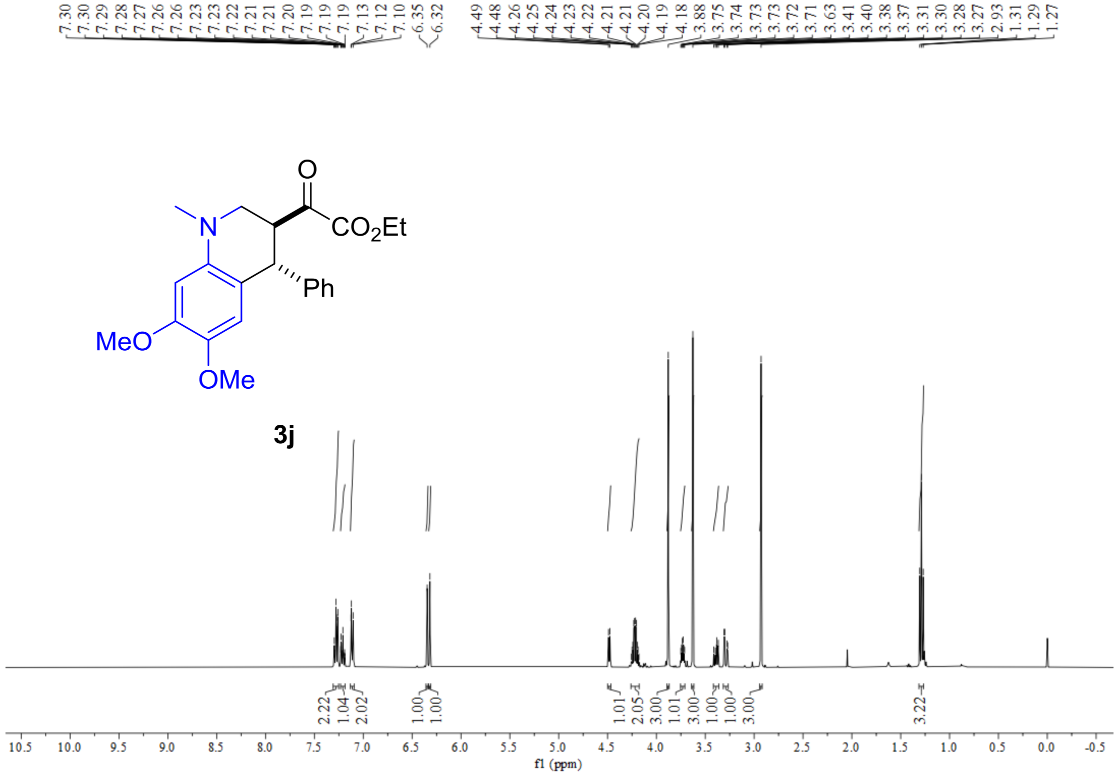

^{13}C-NMR of **3j** (101 MHz, $CDCl_3$)

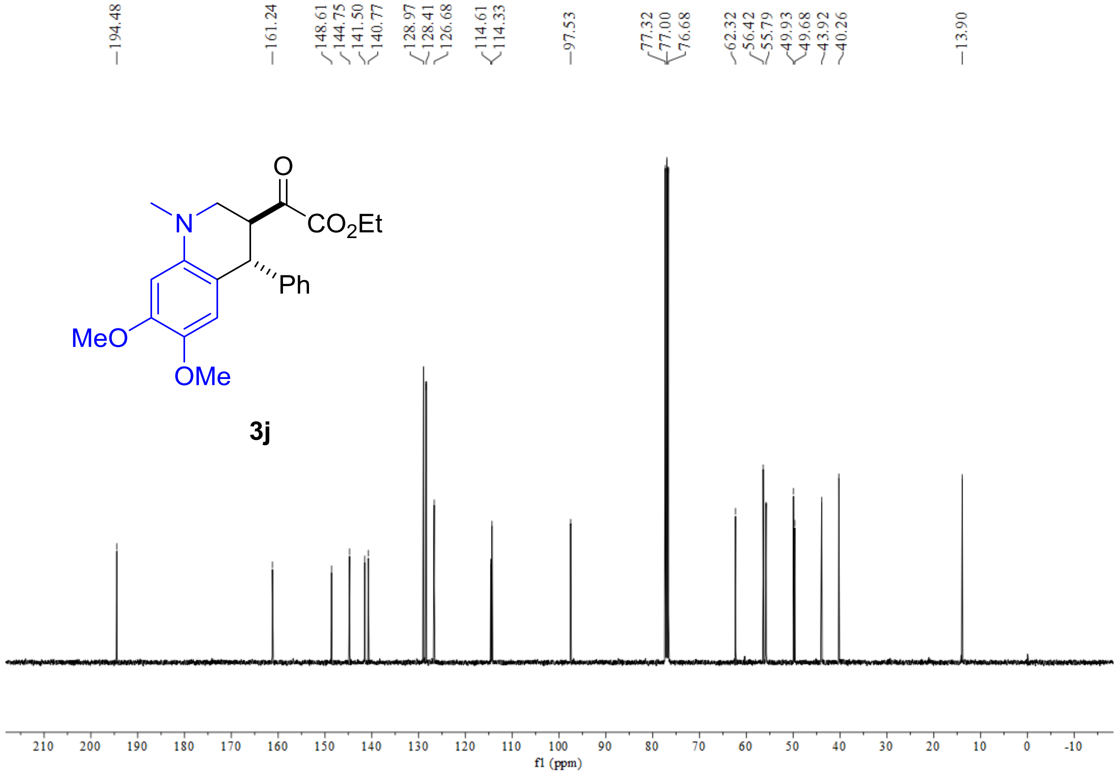

^{1}H-NMR of **3k** (400 MHz, $CDCl_3$)

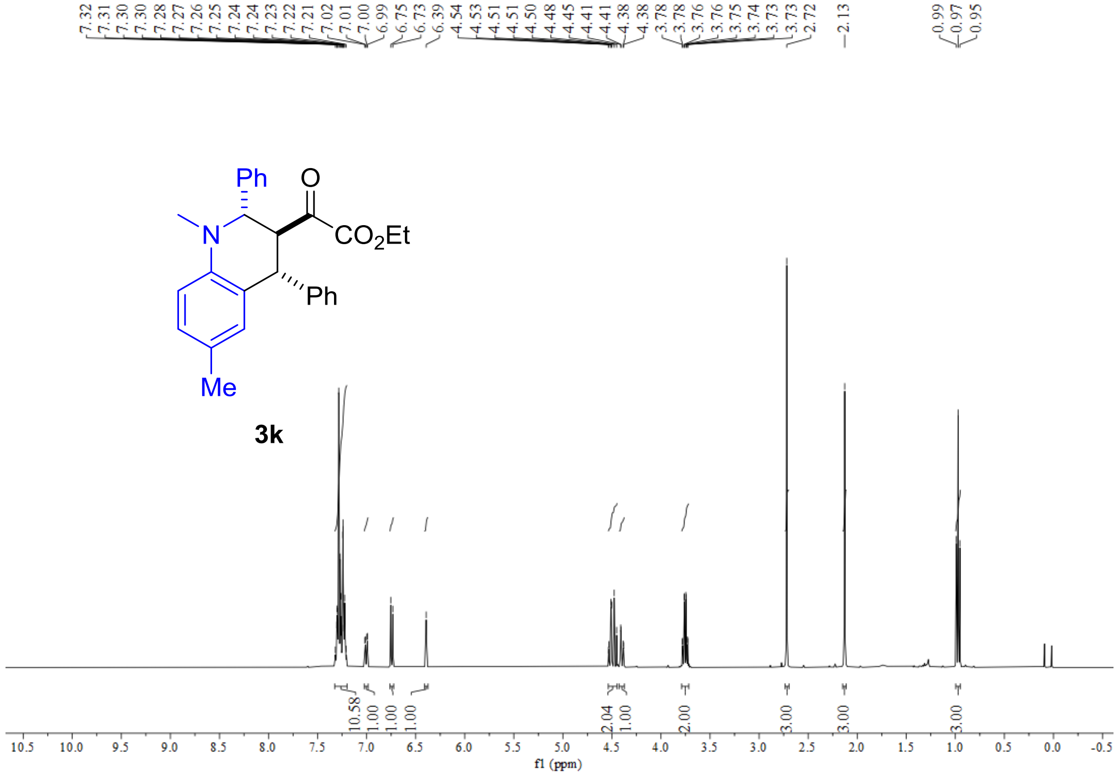

^{13}C-NMR of **3k** (101 MHz, $CDCl_3$)

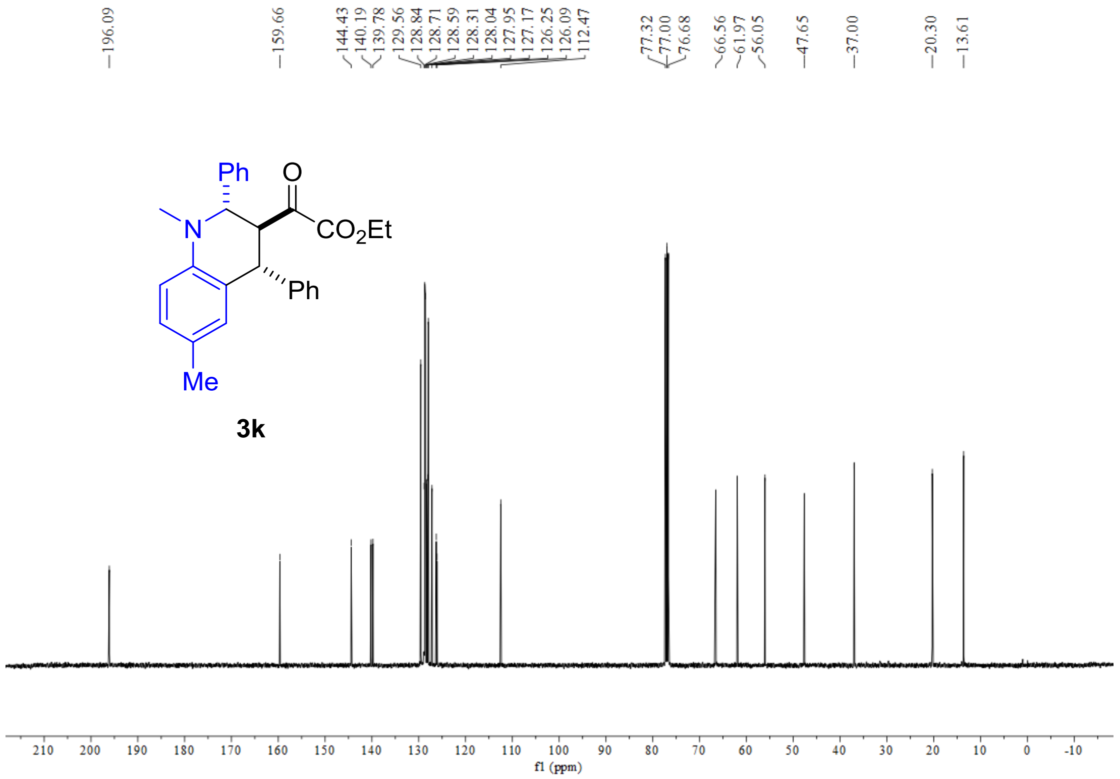

^{1}H-NMR of 3k’ (400 MHz, $CDCl_3$)

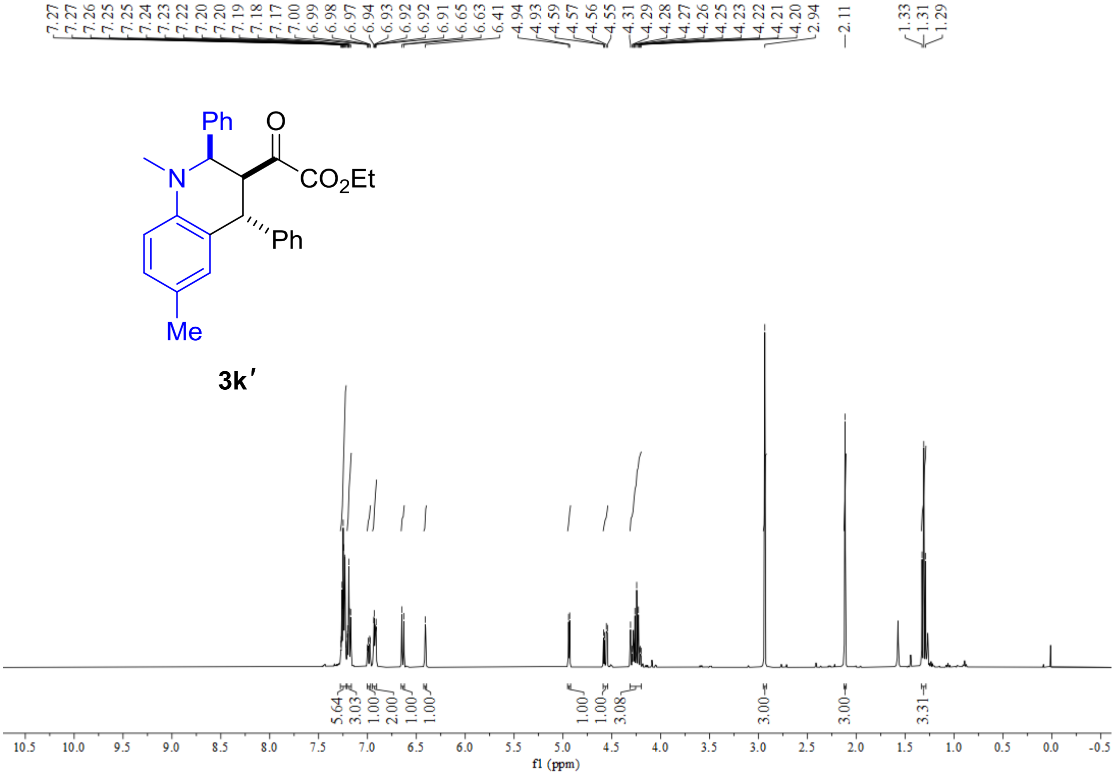

^{13}C-NMR of 3k’ (101 MHz, $CDCl_3$)

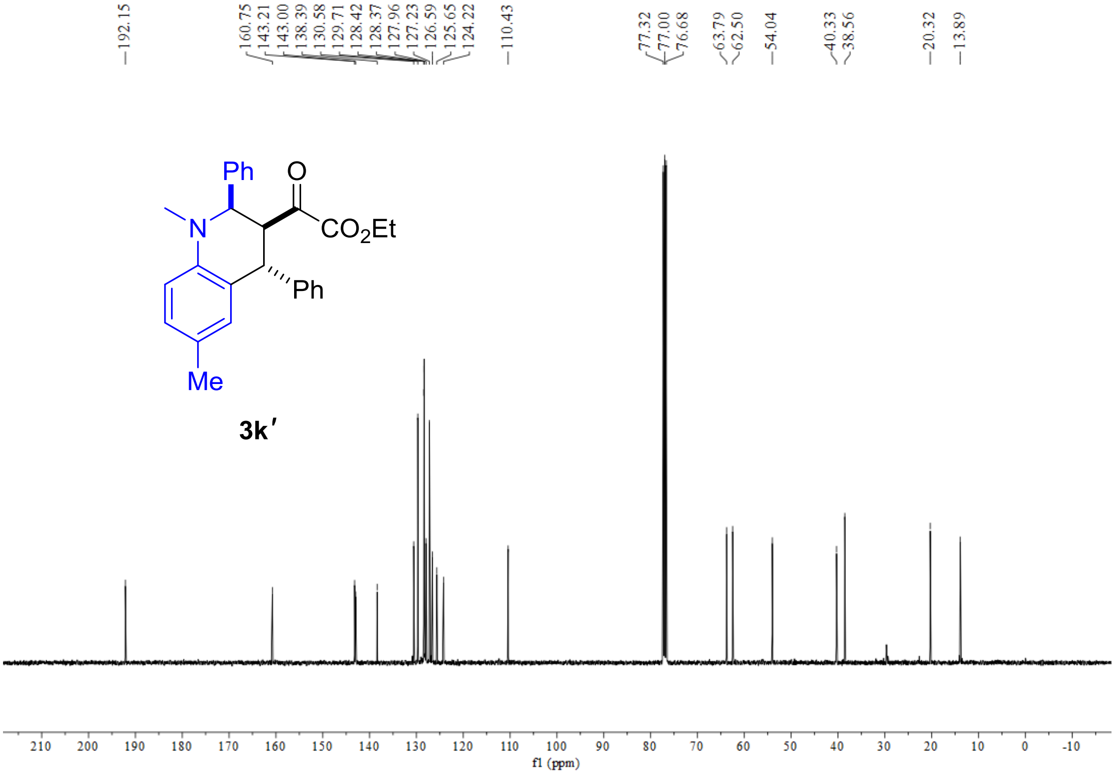

^{1}H-NMR of **3l** (400 MHz, $CDCl_3$)

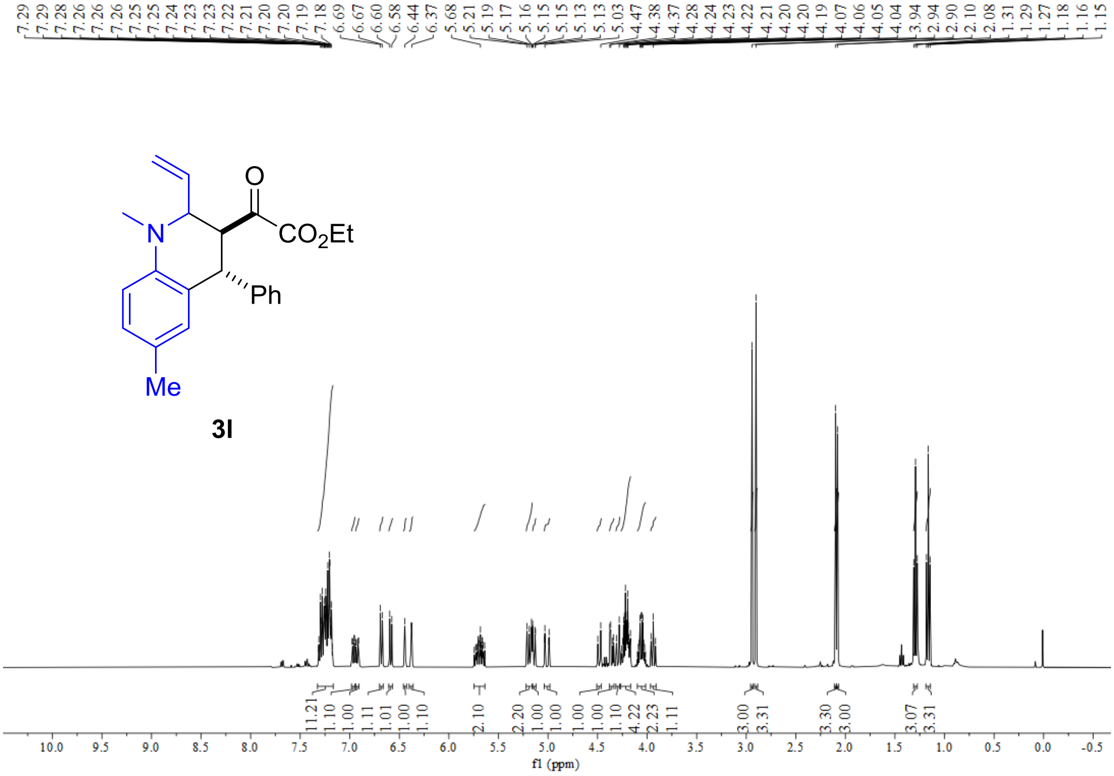

^{13}C-NMR of **3l** (101 MHz, $CDCl_3$)

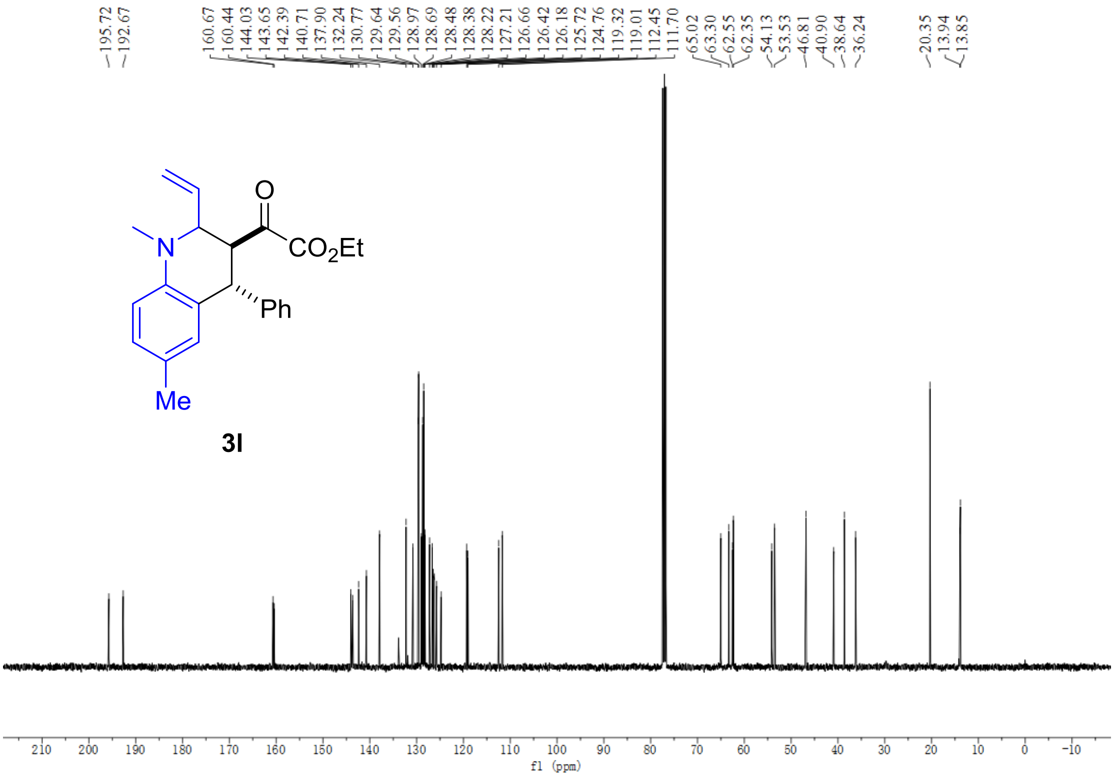

^{1}H-NMR of **3m** (400 MHz, $CDCl_3$)

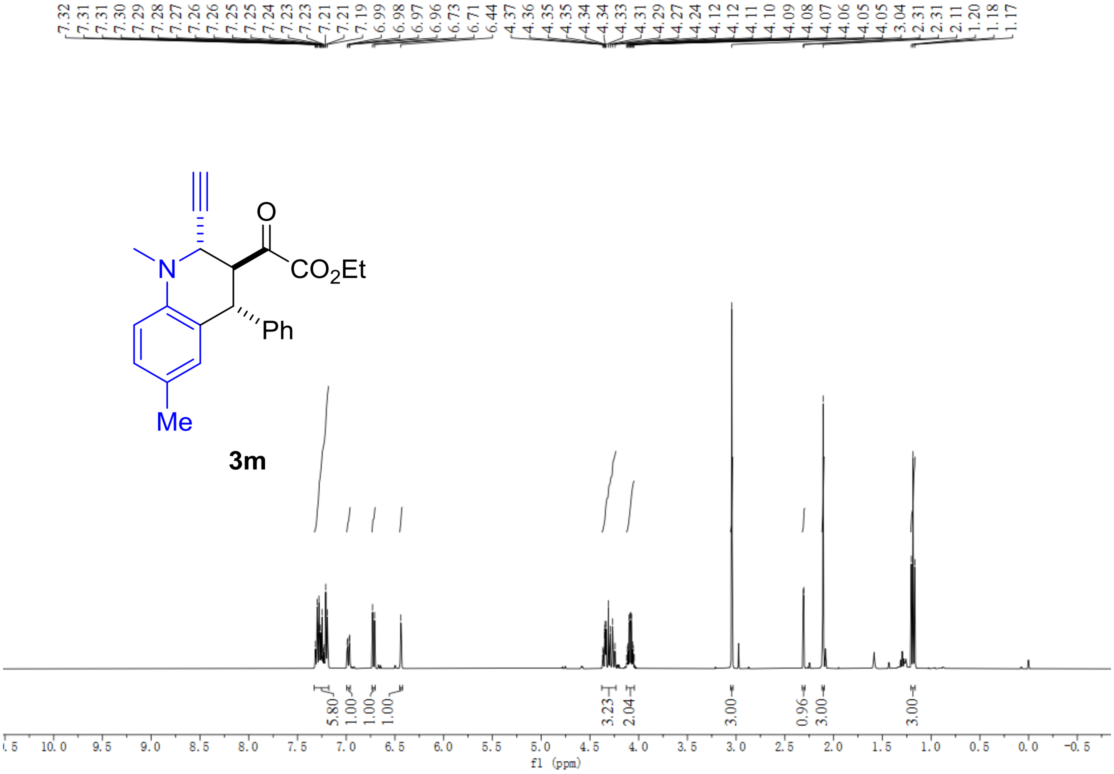

^{13}C-NMR of **3m** (101 MHz, $CDCl_3$)

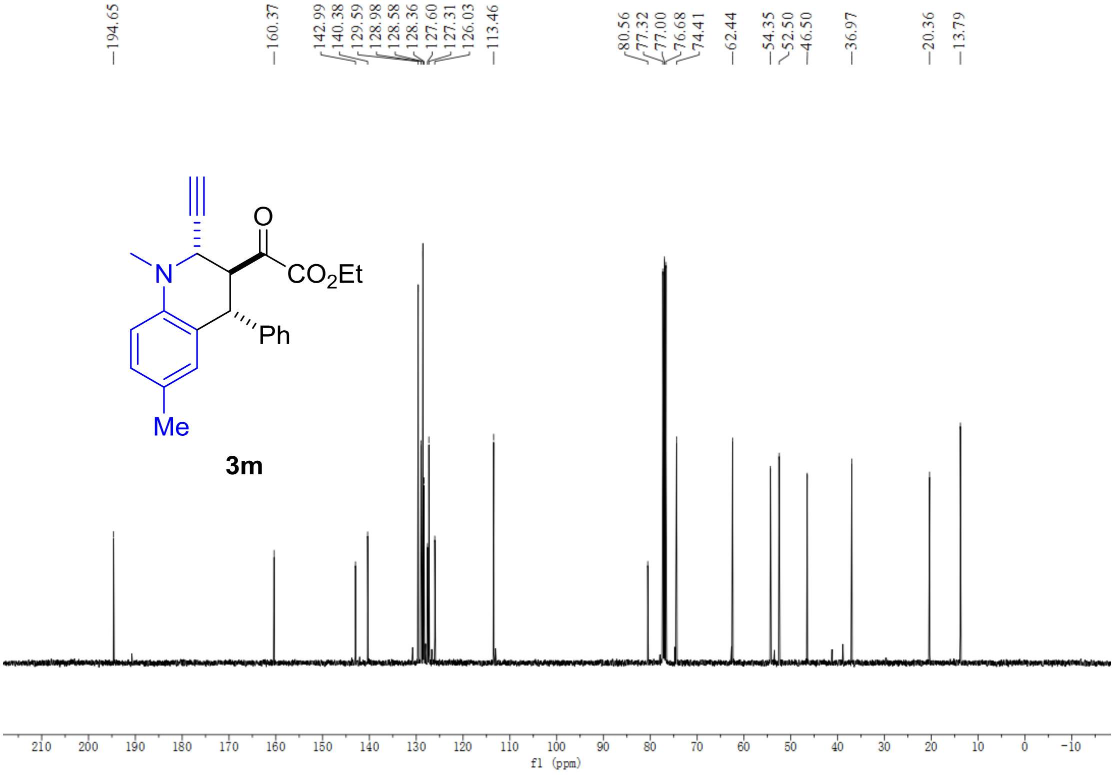

^{1}H-NMR of **3n** (400 MHz, $CDCl_3$)

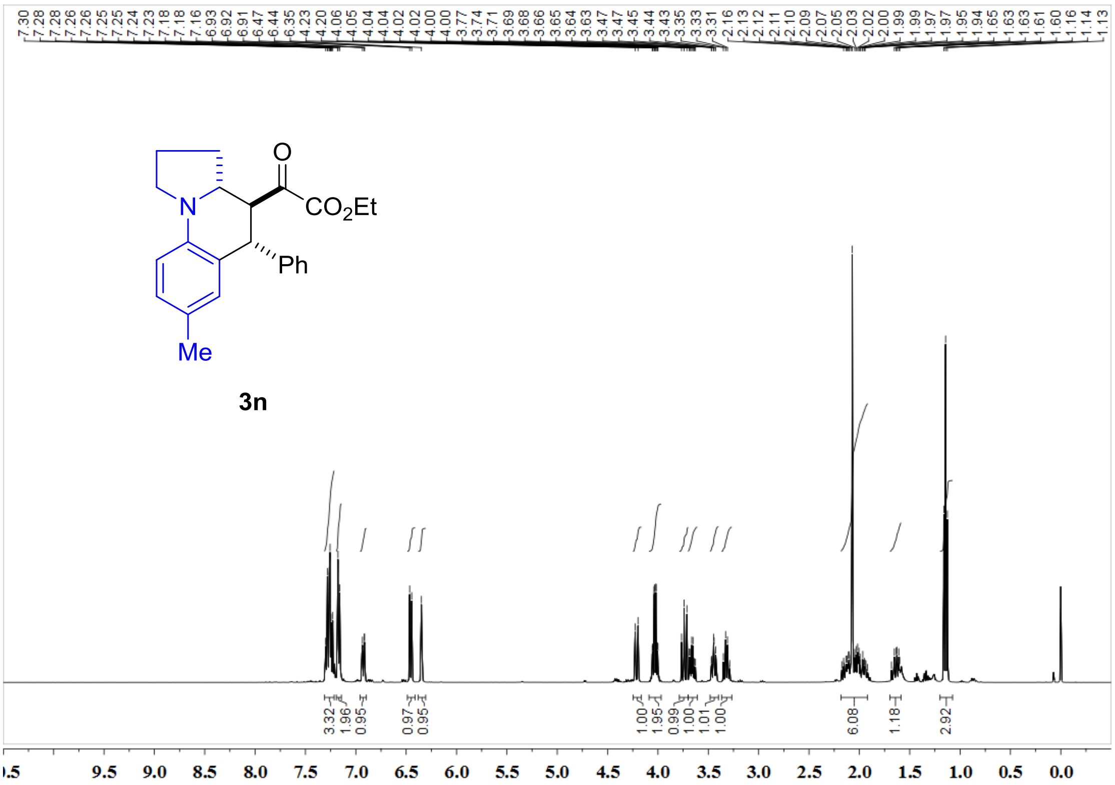

^{13}C-NMR of **3n** (101 MHz, $CDCl_3$)

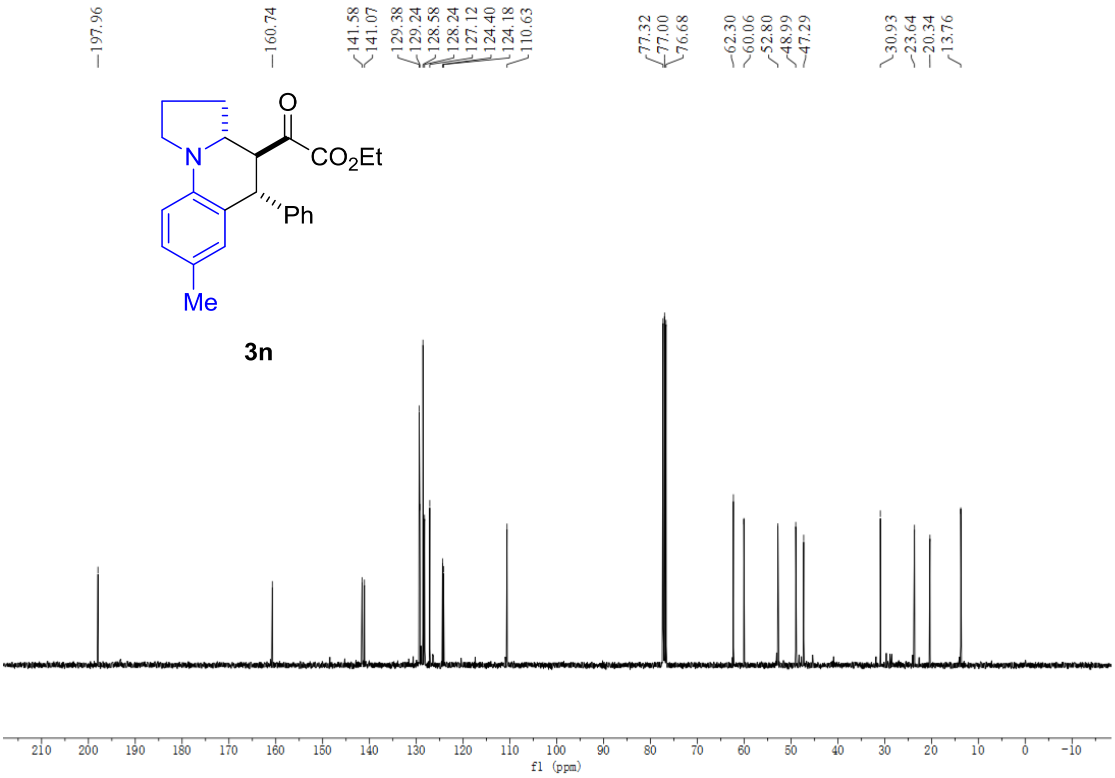

^{1}H,^{1}H-NOESY of **3n** (400 MHz, $CDCl_3$)

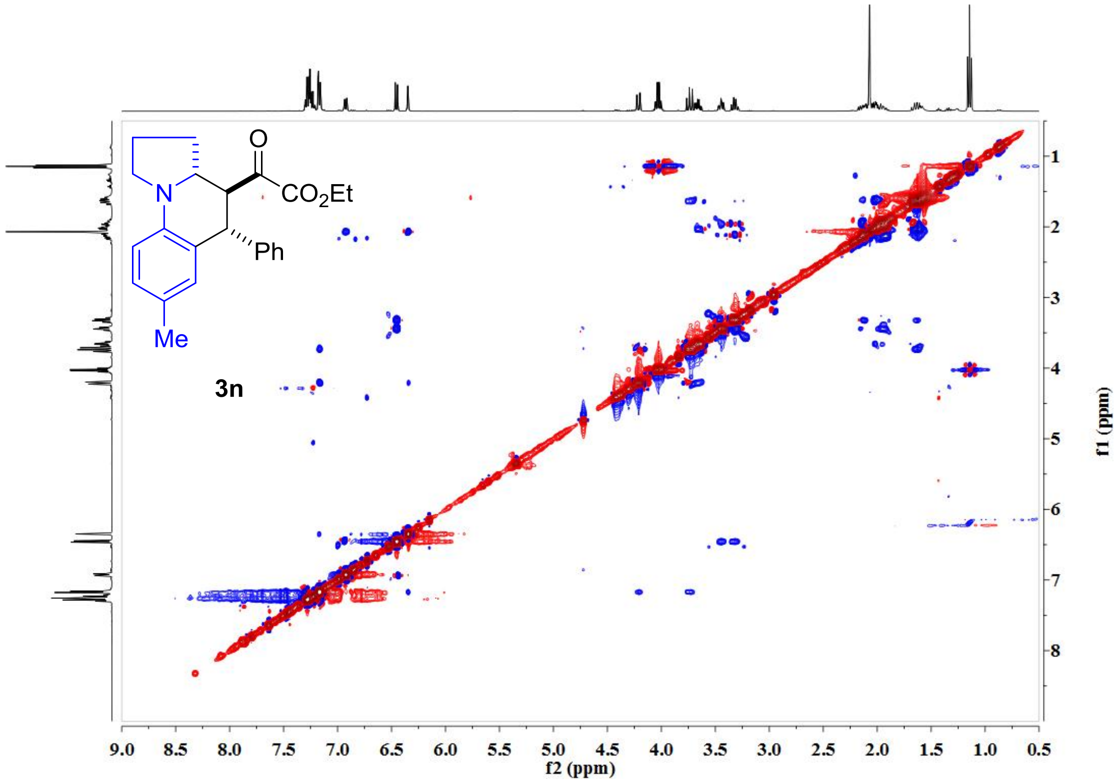

^{1}H-NMR of **3o** (400 MHz, $CDCl_3$)

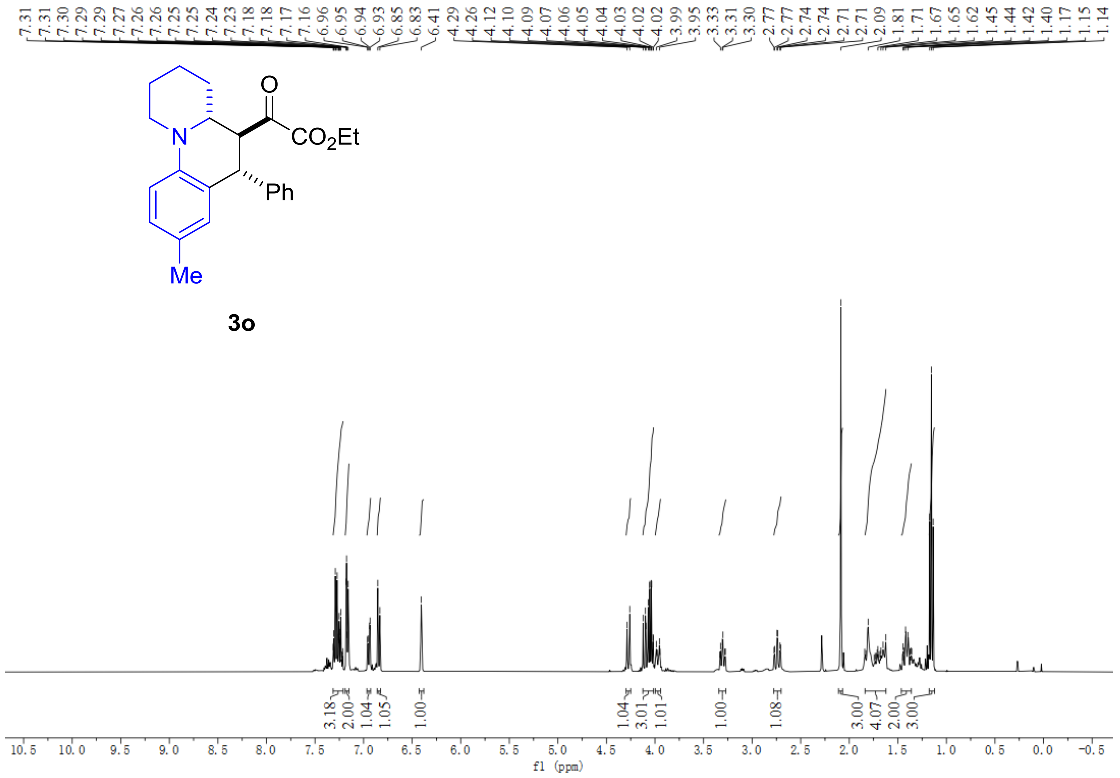

^{13}C-NMR of **3o** (101 MHz, $CDCl_3$)

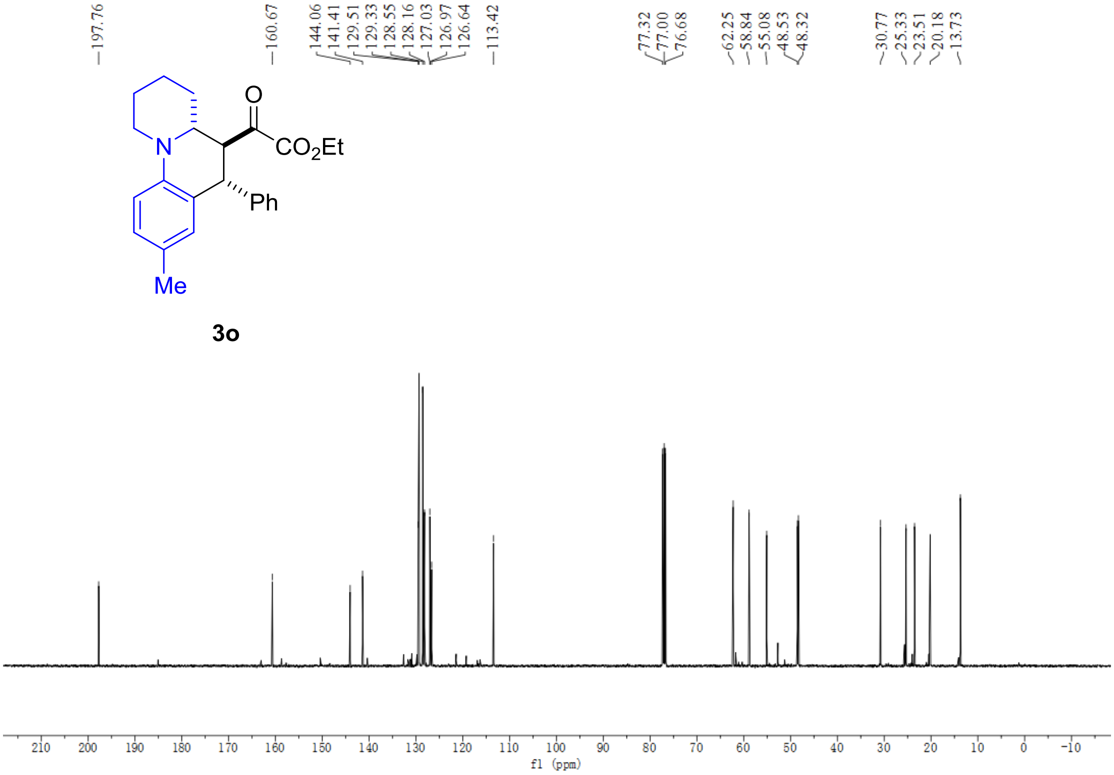

^{1}H-NMR of **3p** (400 MHz, $CDCl_3$)

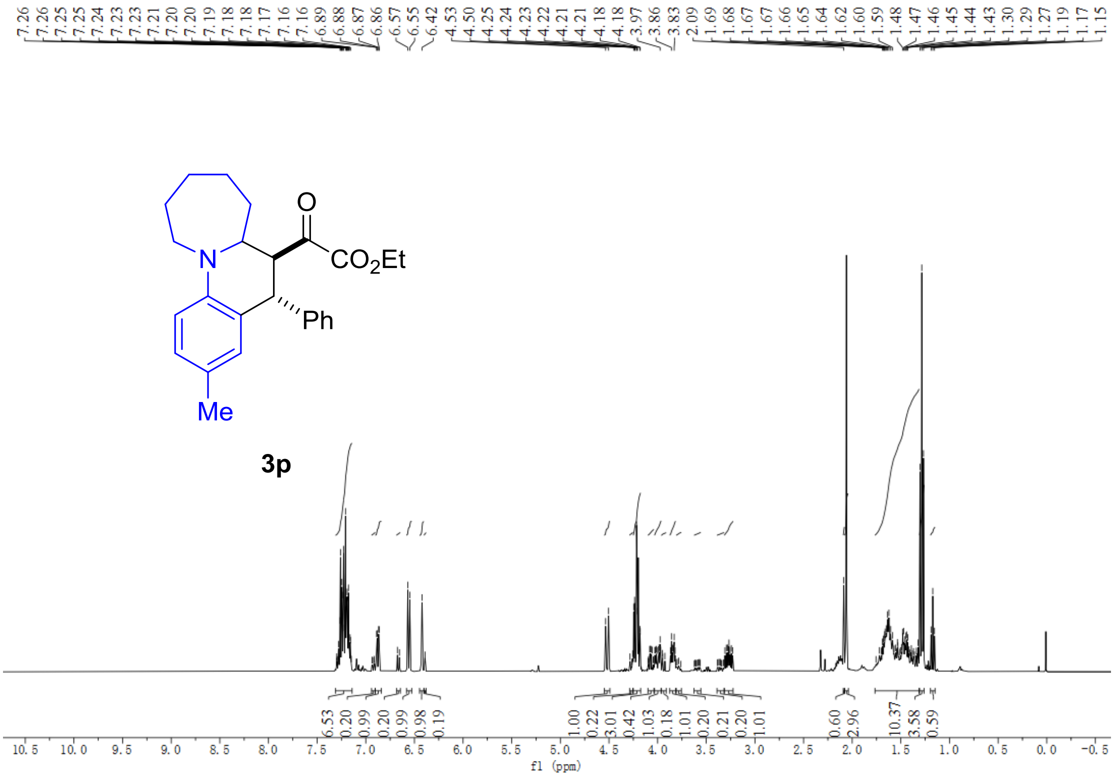

^{13}C-NMR of 3p (101 MHz, $CDCl_3$)

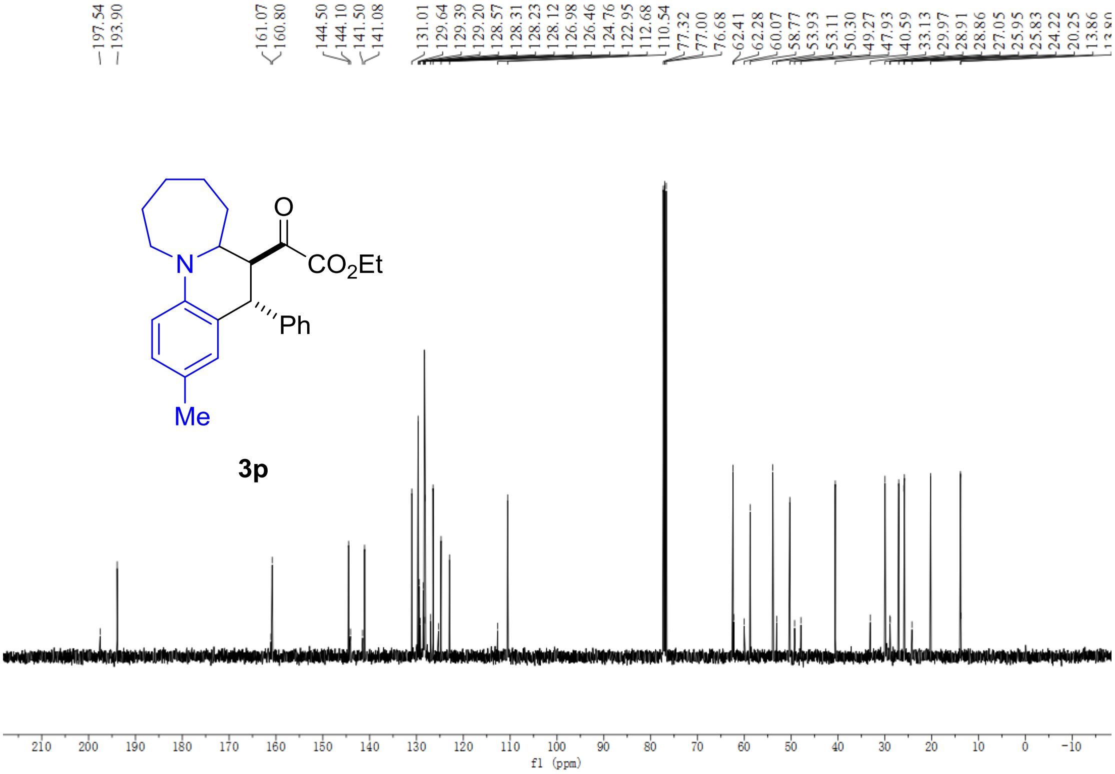

^{1}H-NMR of 3q (400 MHz, $CDCl_3$)

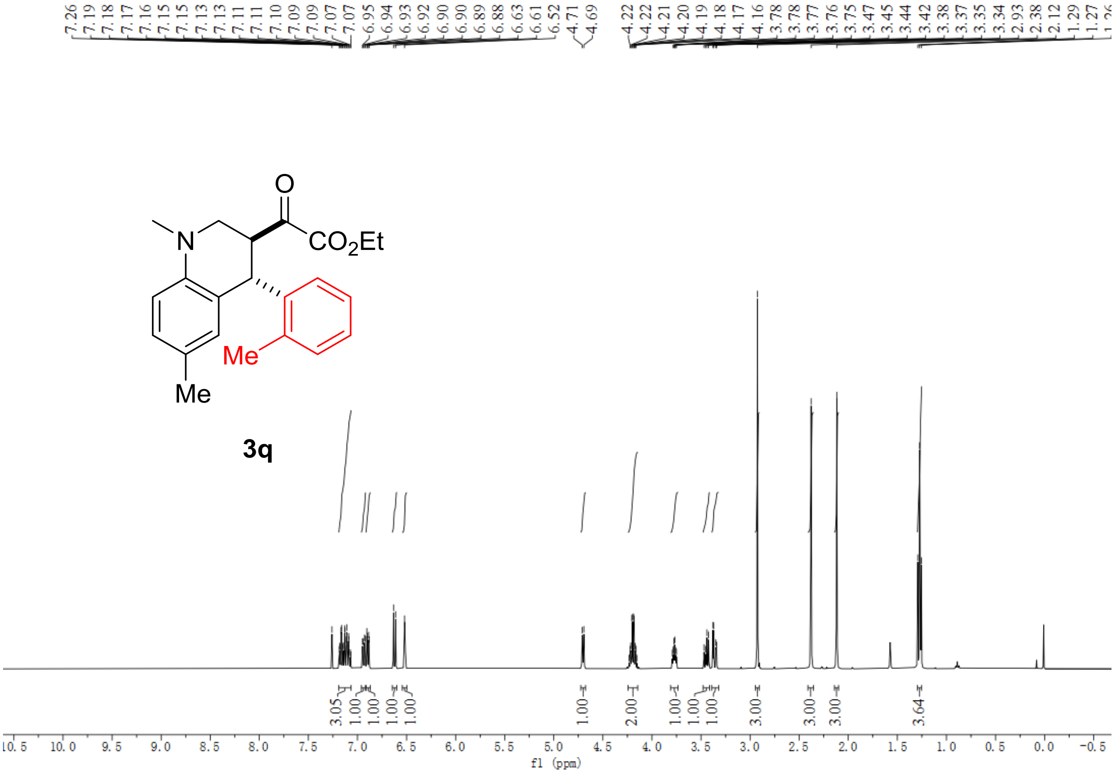

^{13}C-NMR of 3q (101 MHz, $CDCl_3$)

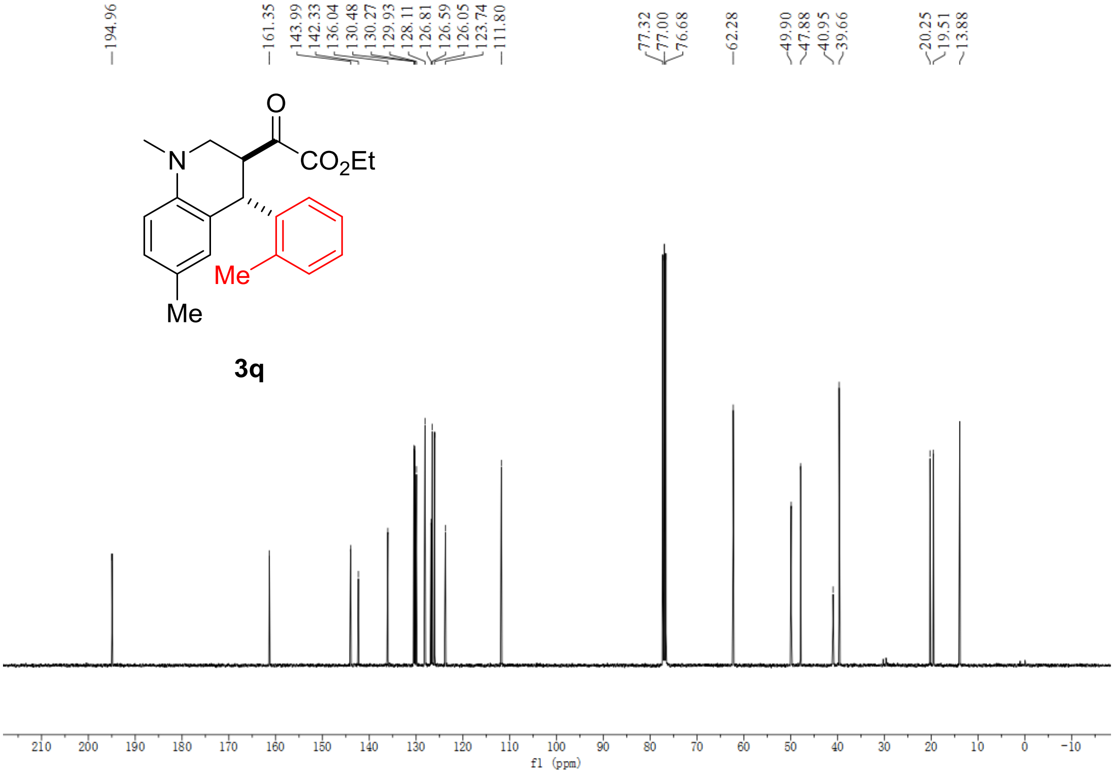

^{1}H-NMR of 3r (400 MHz, $CDCl_3$)

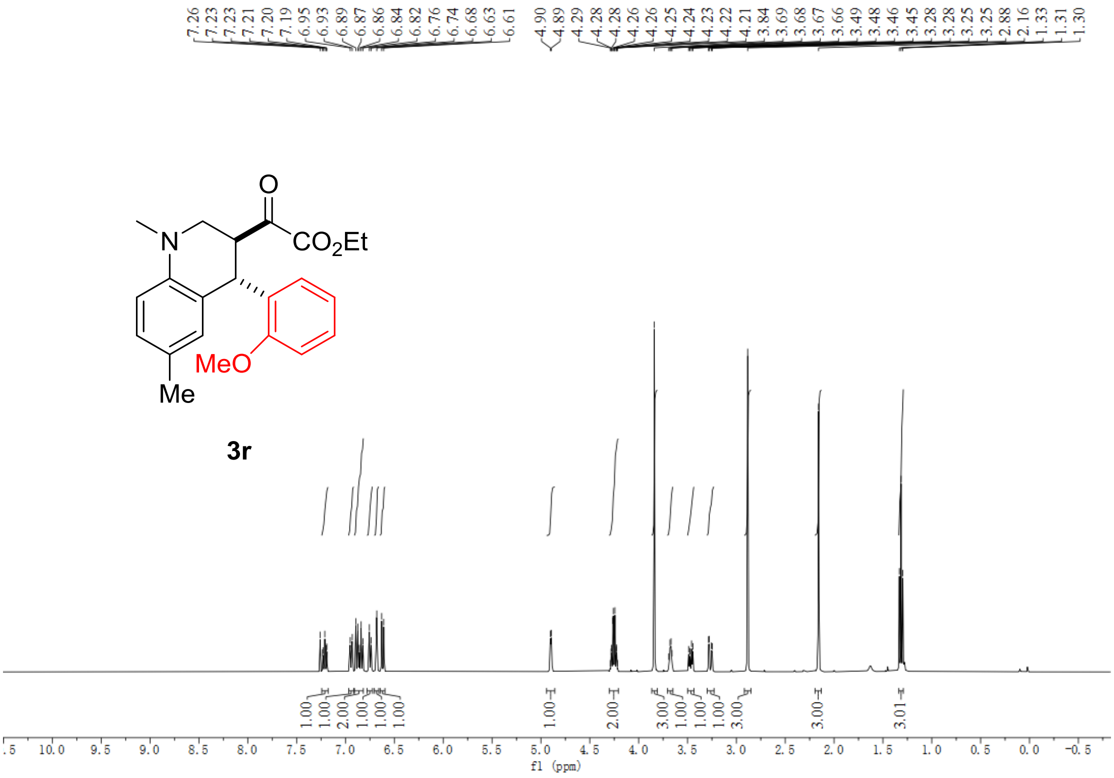

^{13}C-NMR of **3r** (101 MHz, $CDCl_3$)

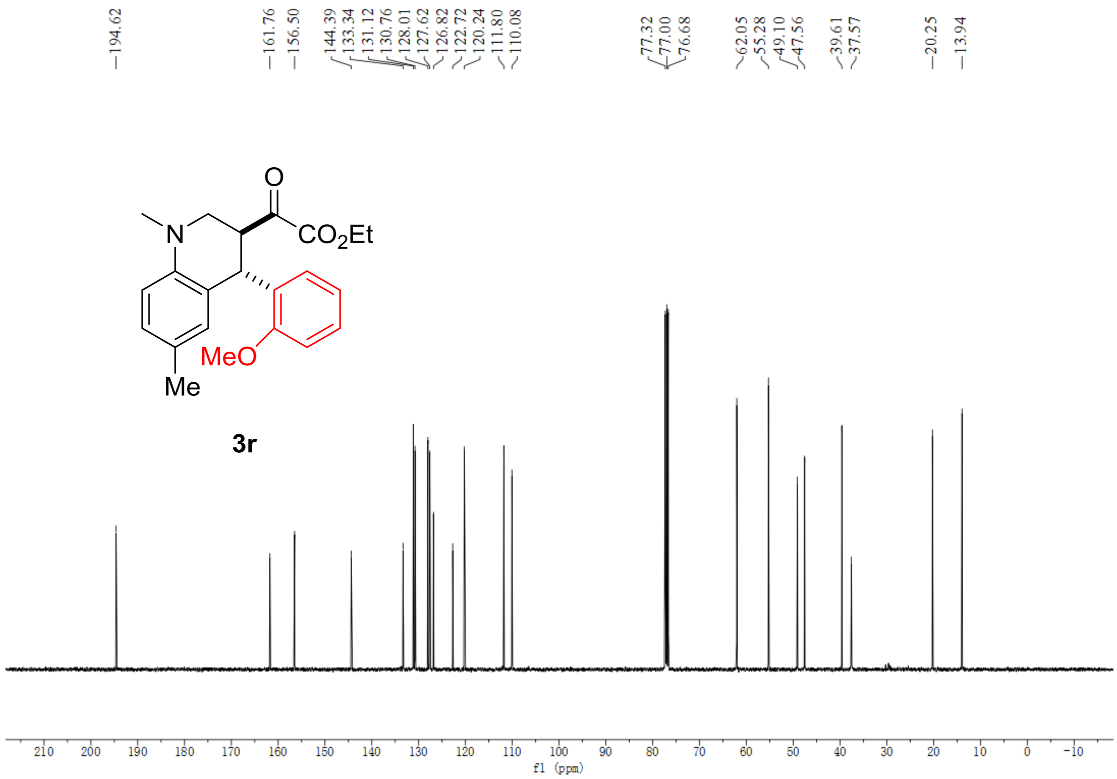

^{1}H-NMR of **3s** (400 MHz, $CDCl_3$)

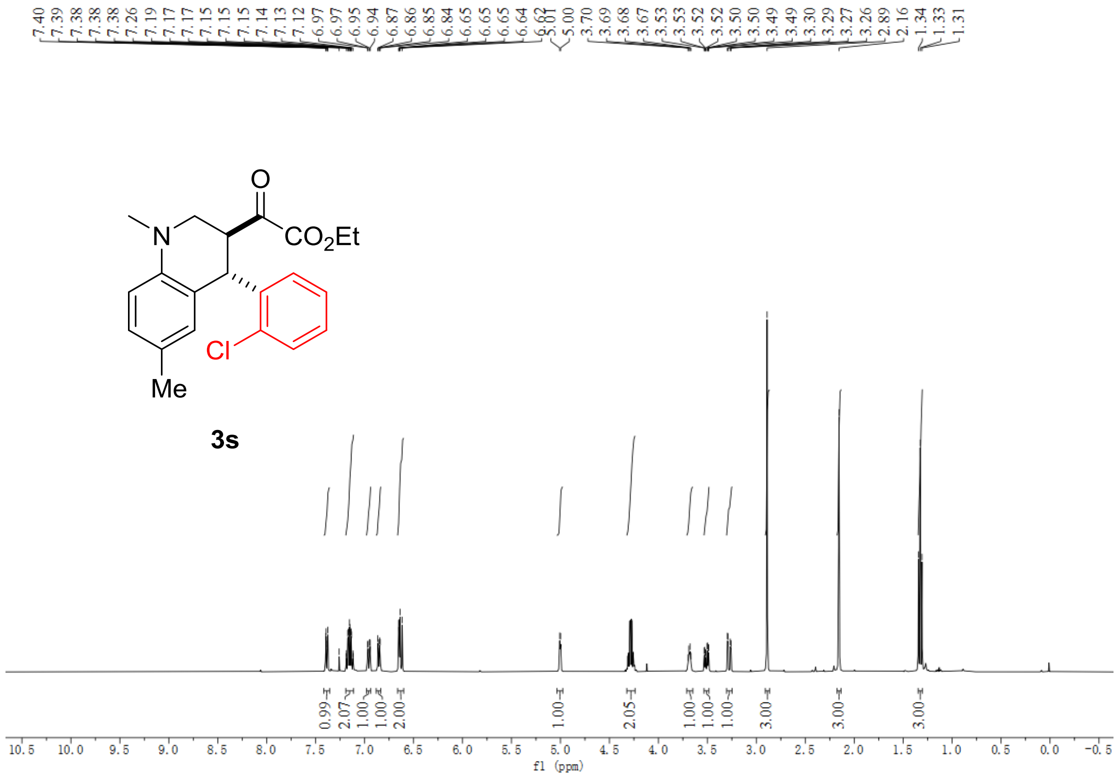

^{13}C-NMR of 3s (101 MHz, $CDCl_3$)

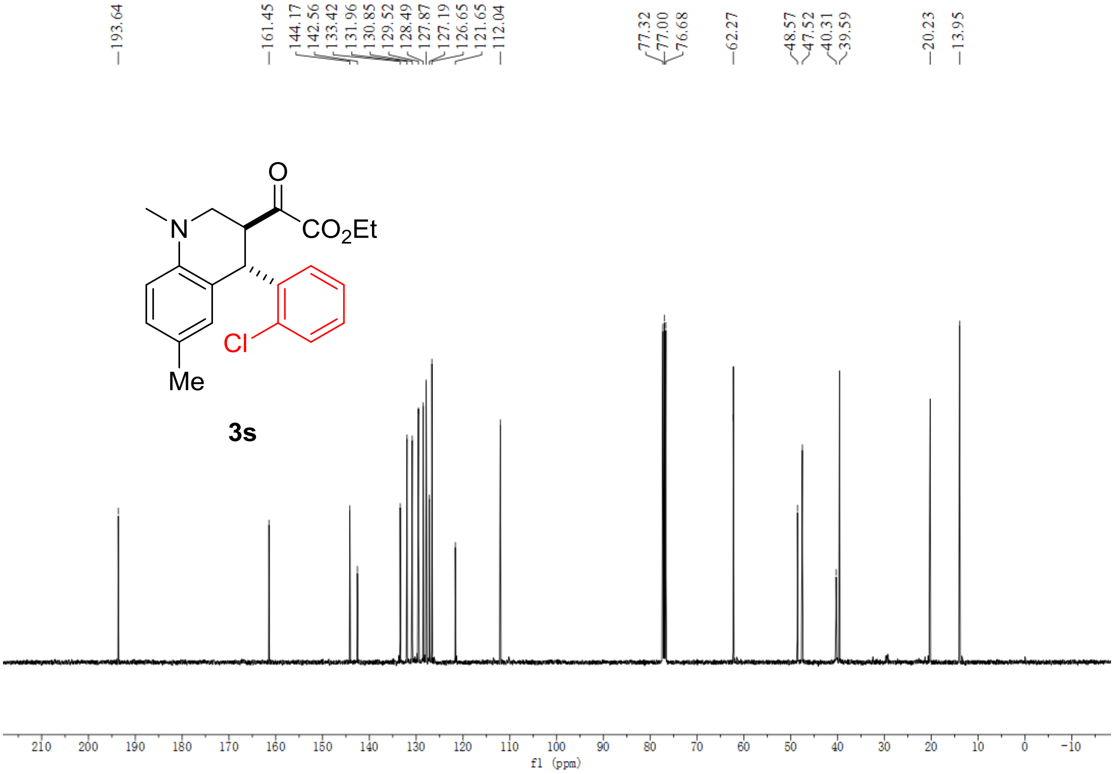

^{1}H-NMR of 3t (400 MHz, $CDCl_3$)

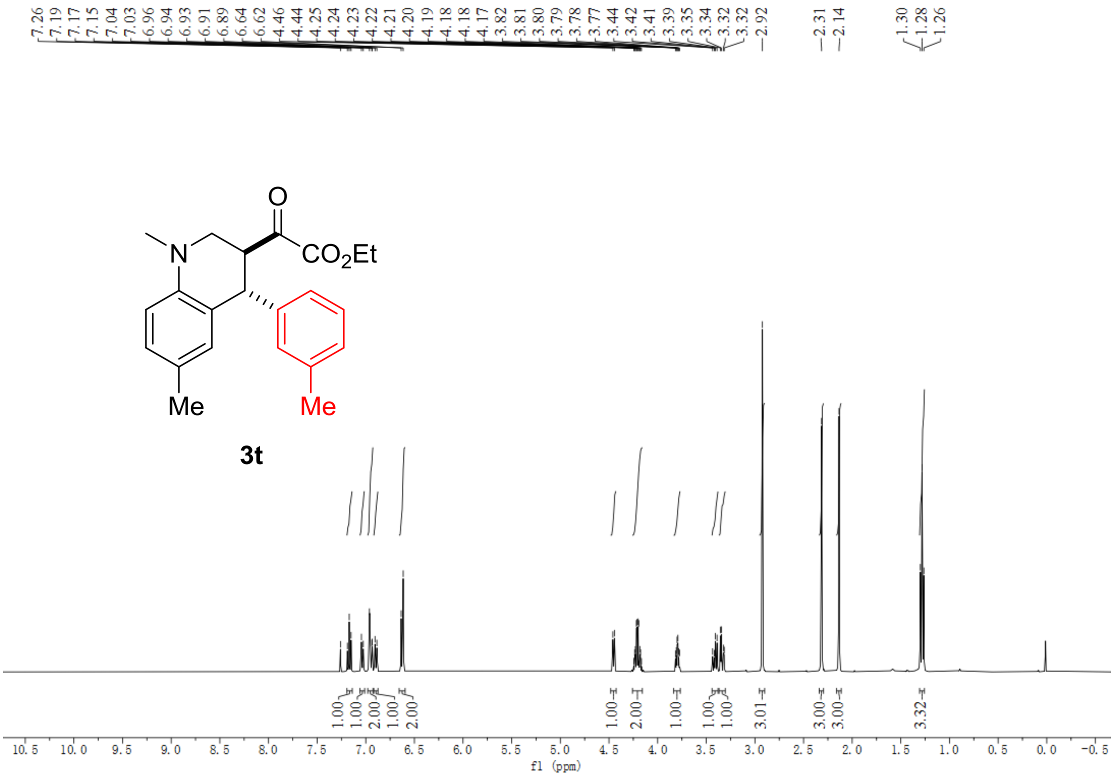

^{13}C-NMR of **3t** (101 MHz, $CDCl_3$)

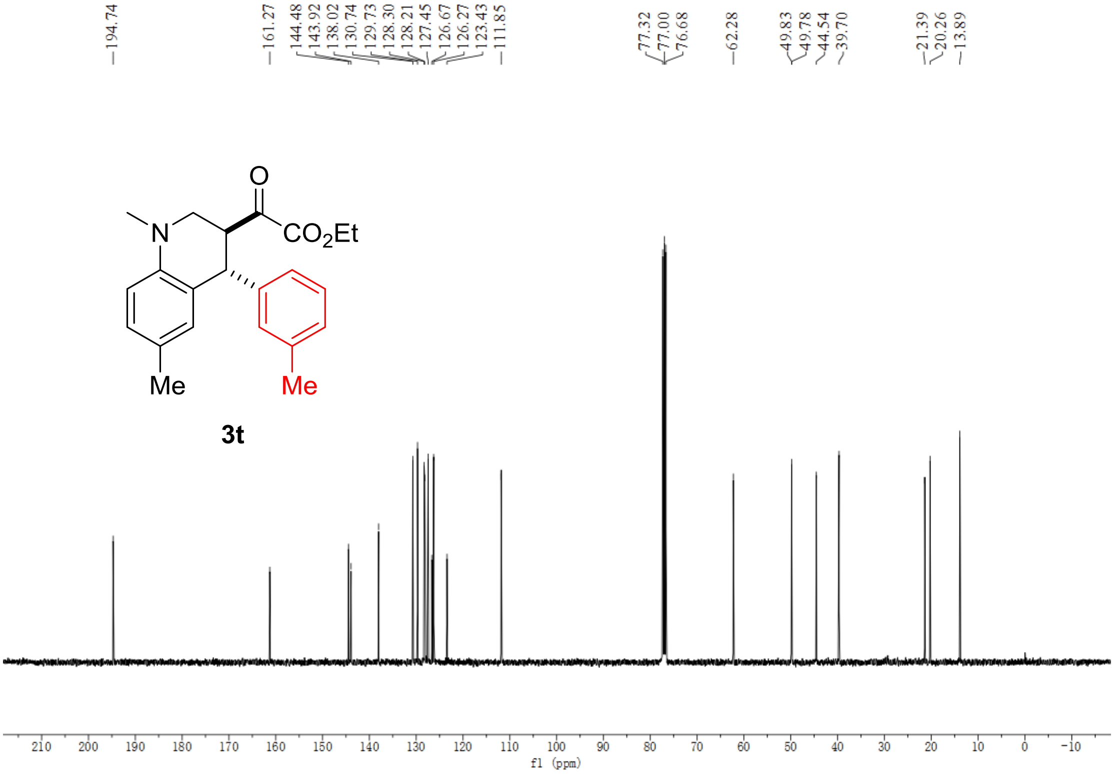

^{1}H-NMR of **3u** (400 MHz, $CDCl_3$)

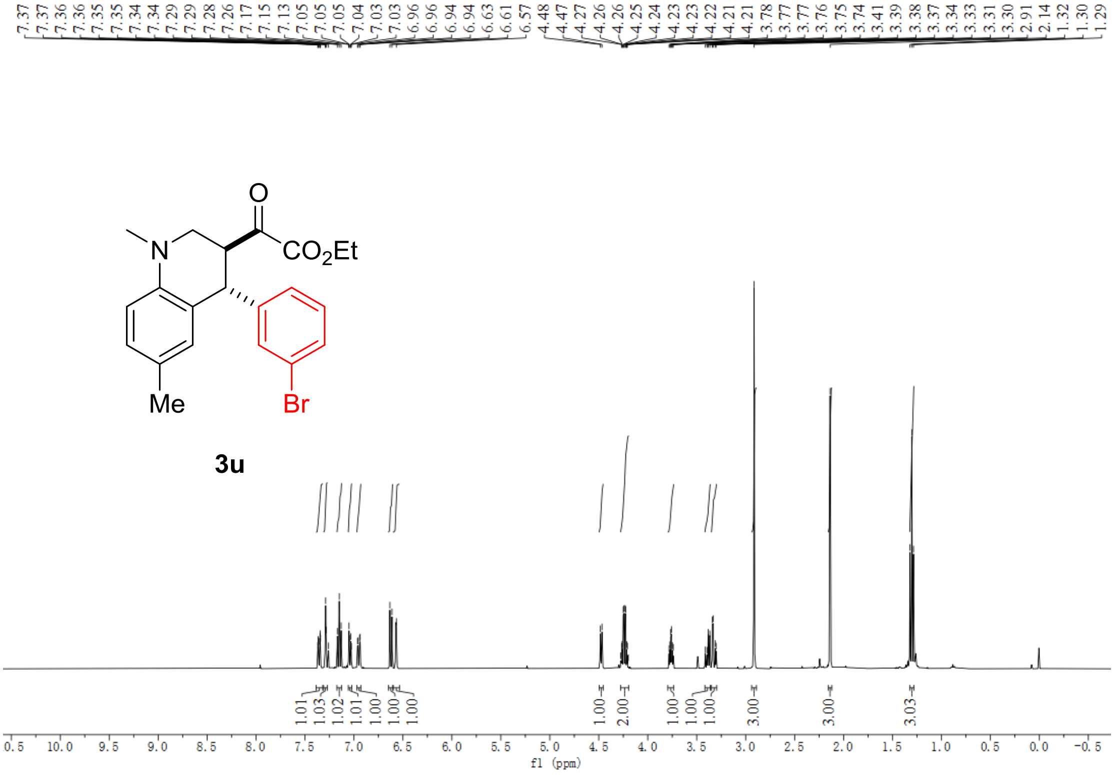

^{13}C-NMR of **3u** (101 MHz, $CDCl_3$)

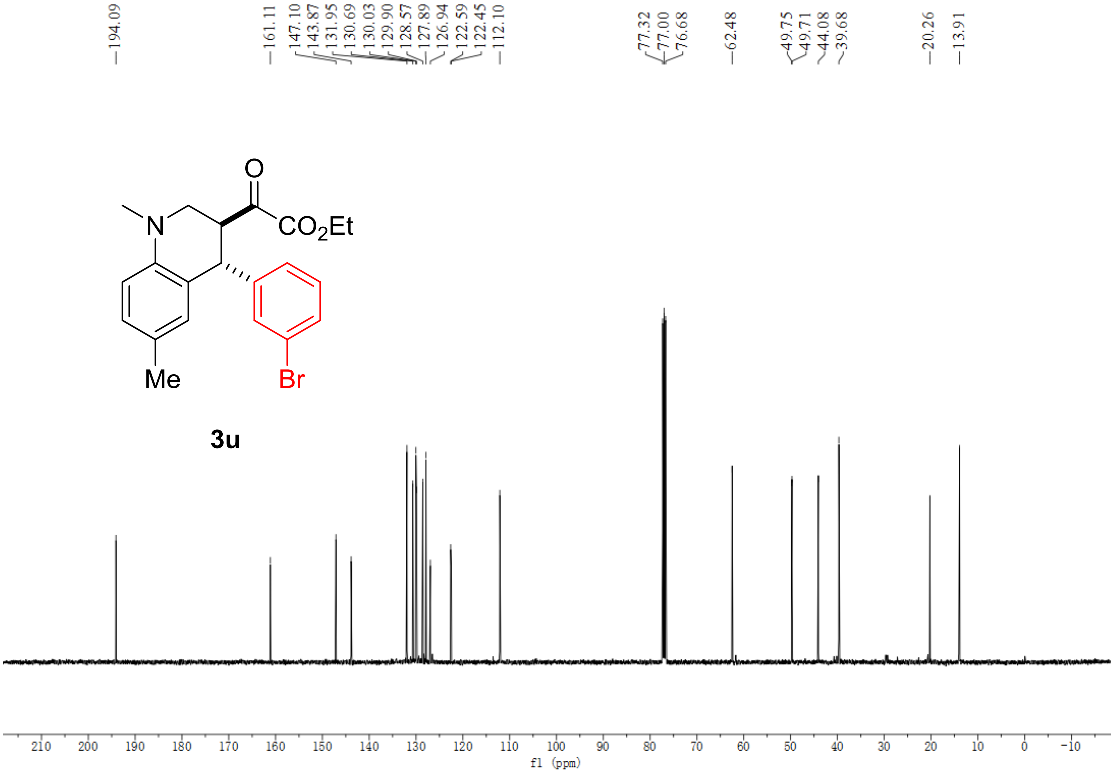

^{1}H-NMR of **3v** (400 MHz, $CDCl_3$)

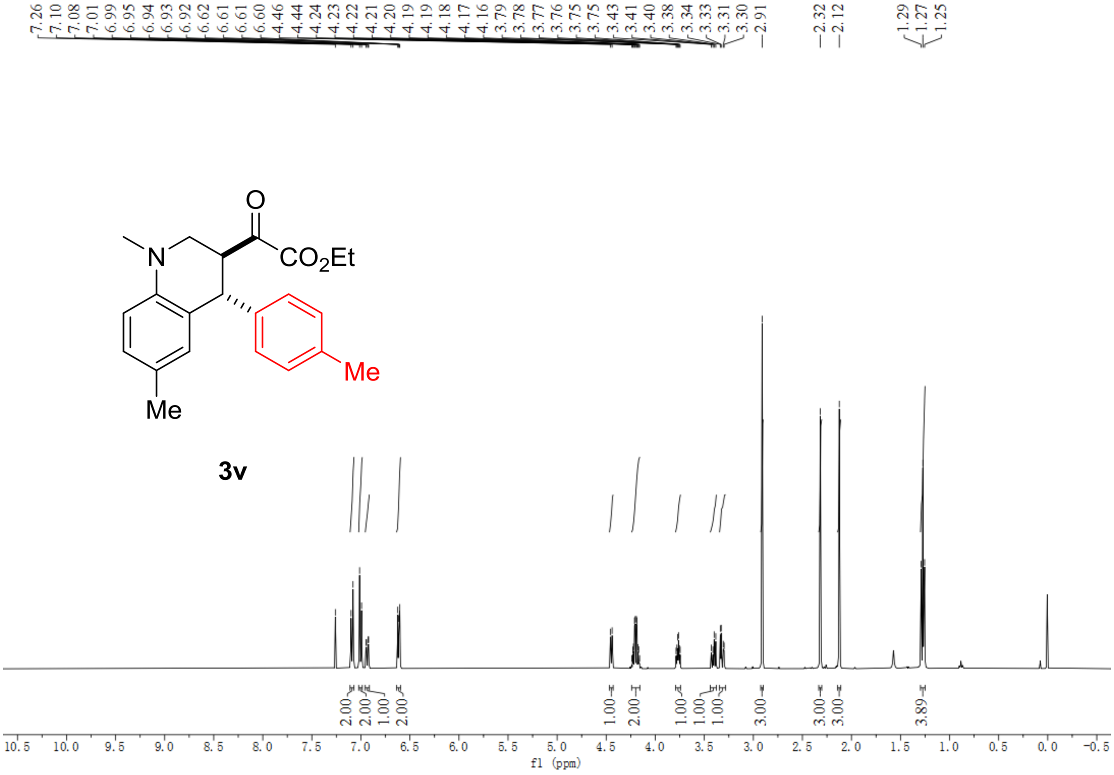

^{13}C-NMR of **3v** (101 MHz, $CDCl_3$)

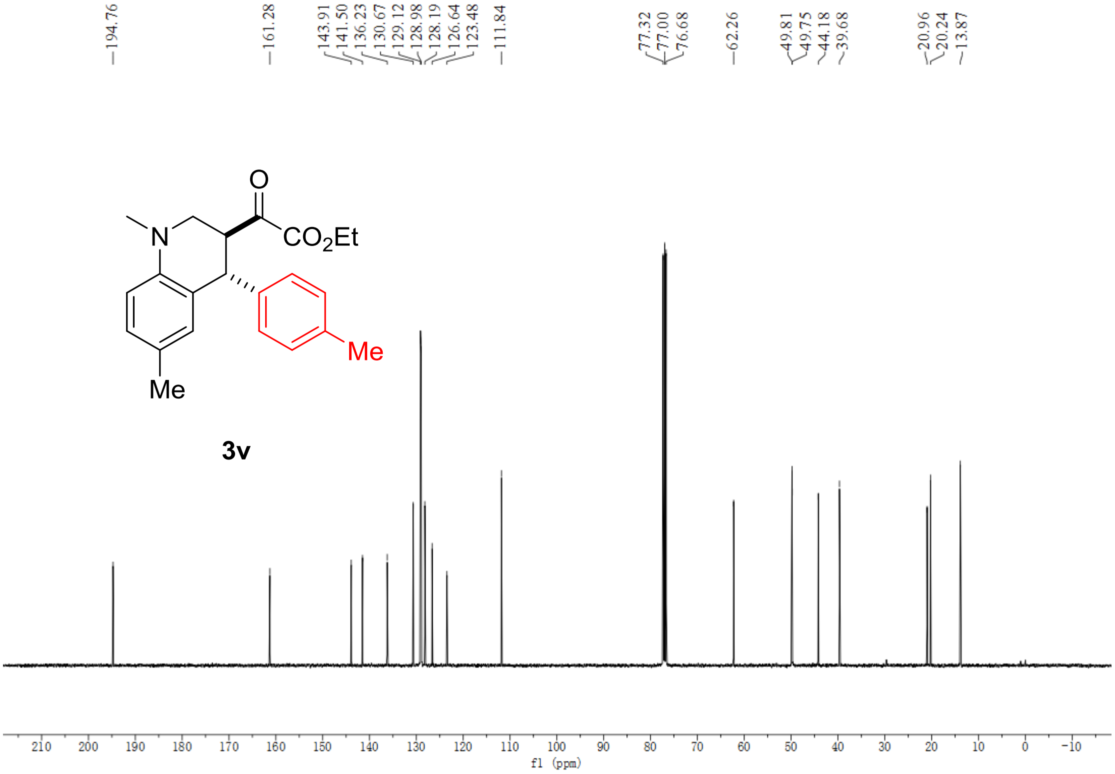

^{1}H-NMR of **3w** (400 MHz, $CDCl_3$)

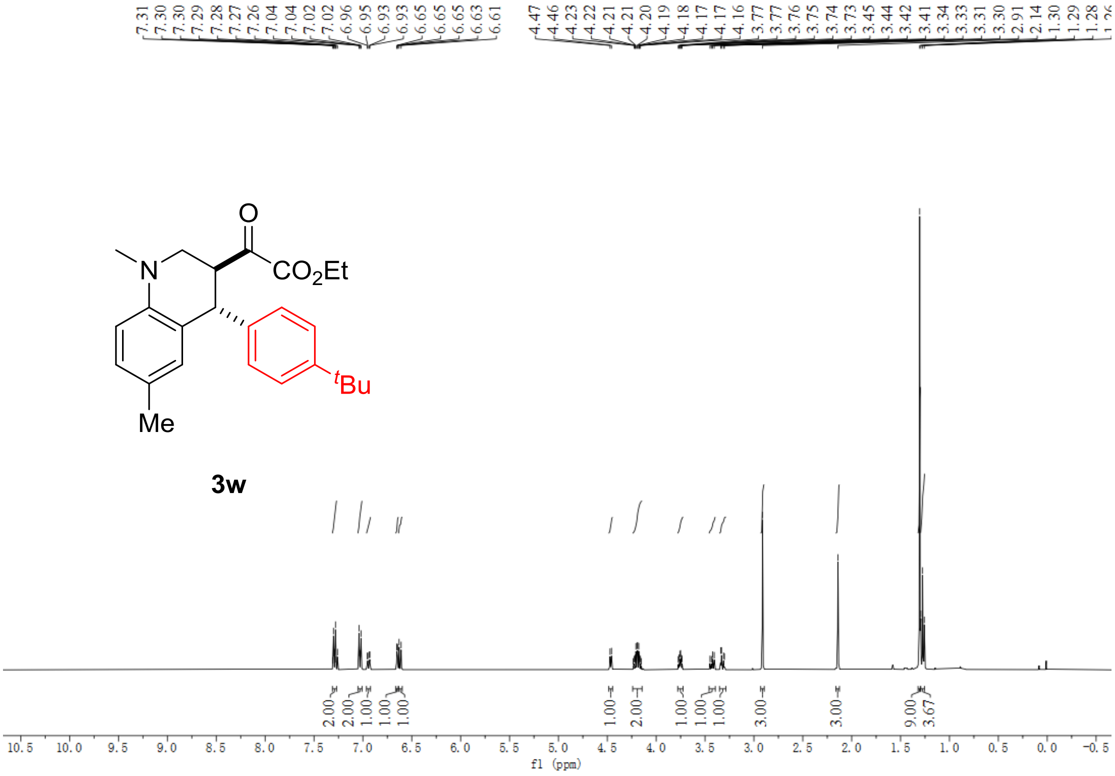

^{13}C-NMR of 3w (101 MHz, $CDCl_3$)

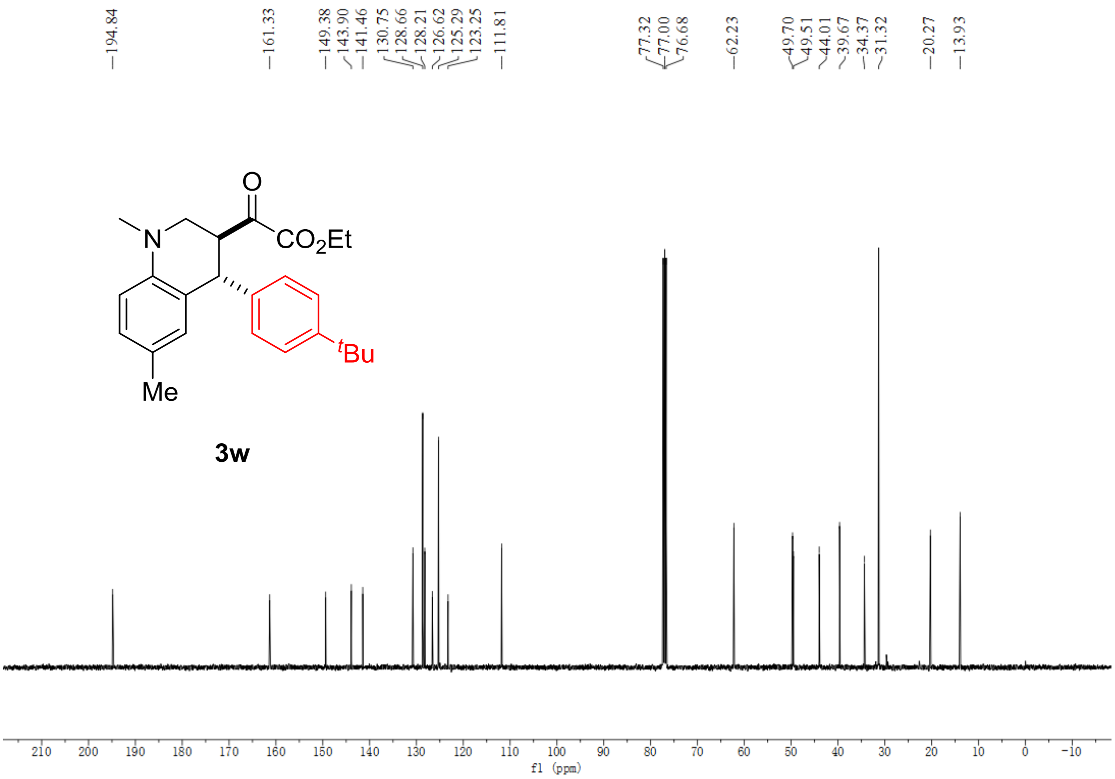

^{1}H-NMR of 3x (400 MHz, $CDCl_3$)

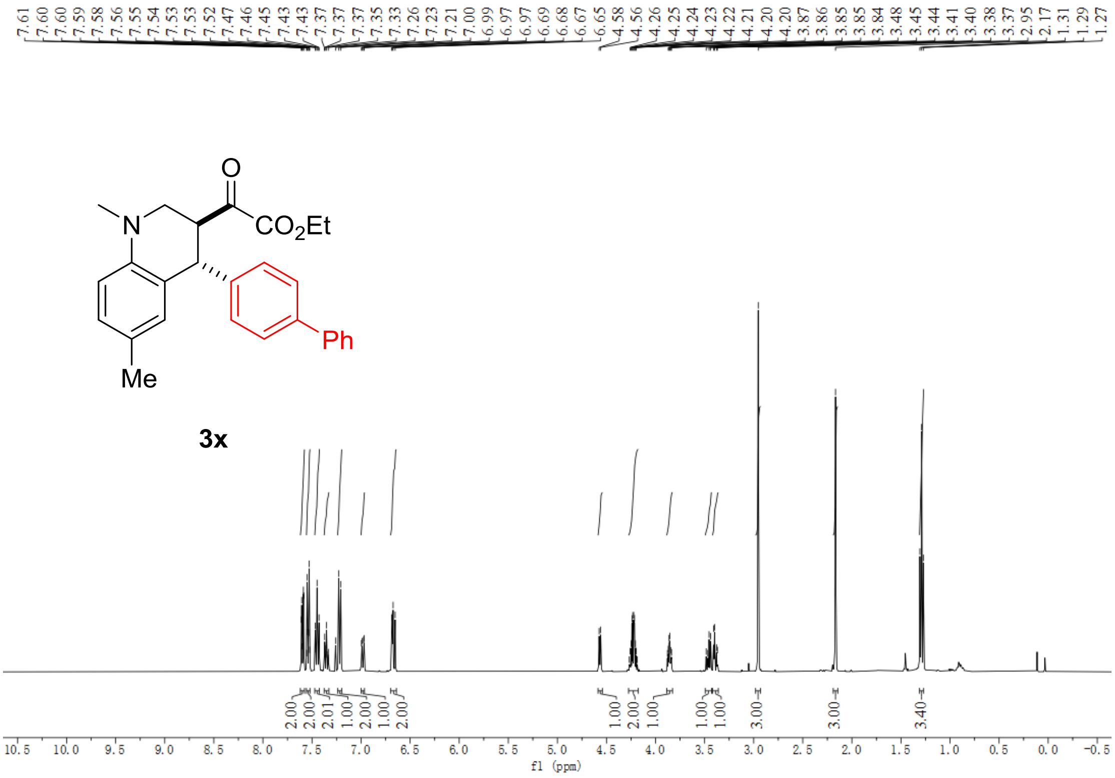

^{13}C-NMR of **3x** (101 MHz, $CDCl_3$)

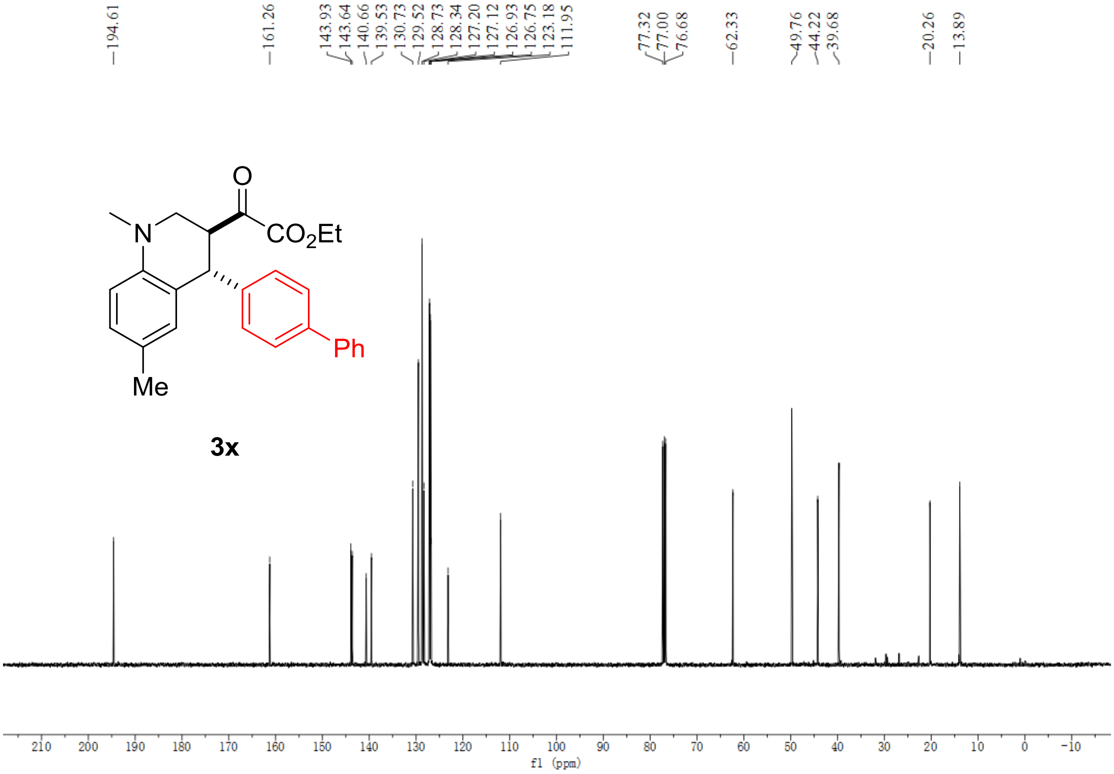

^{1}H-NMR of **3y** (400 MHz, $CDCl_3$)

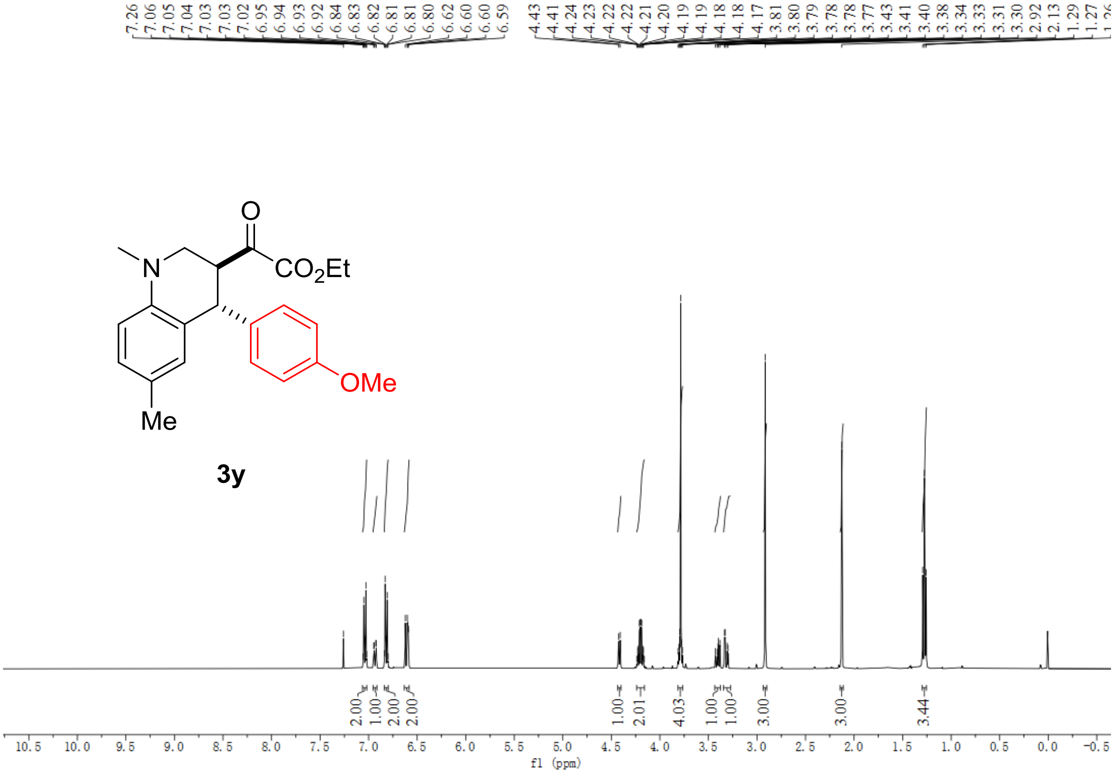

^{13}C-NMR of **3y** (101 MHz, $CDCl_3$)

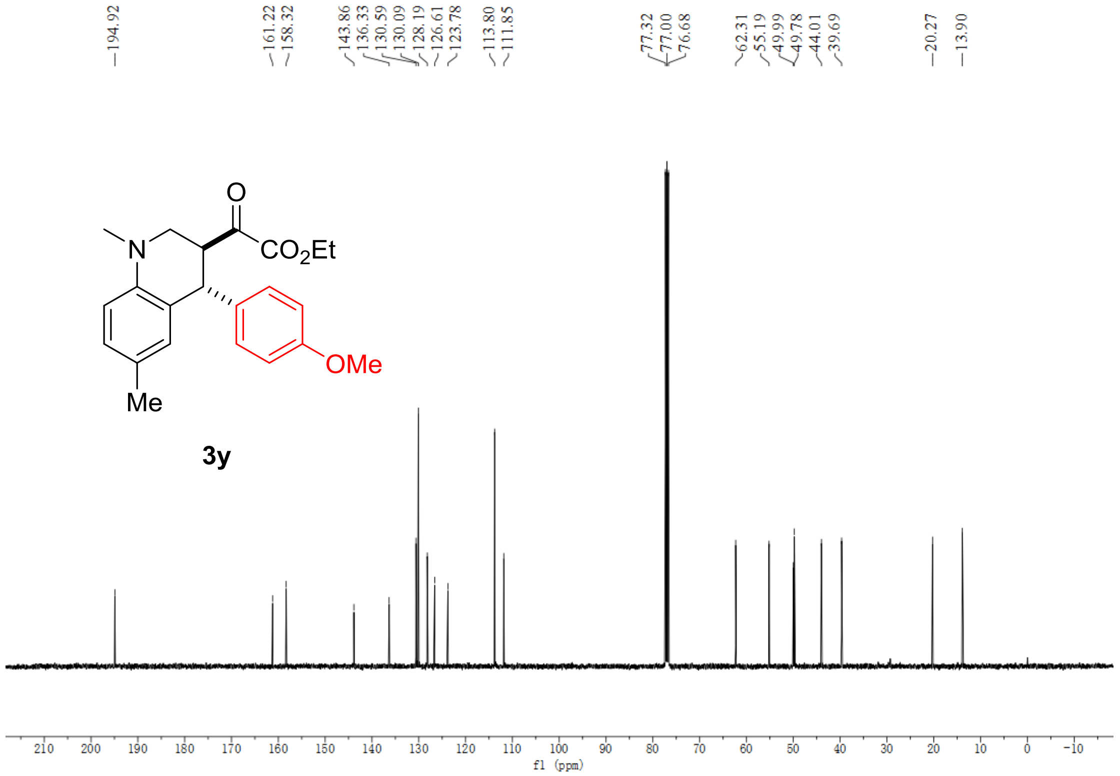

^{1}H-NMR of **3z** (400 MHz, $CDCl_3$)

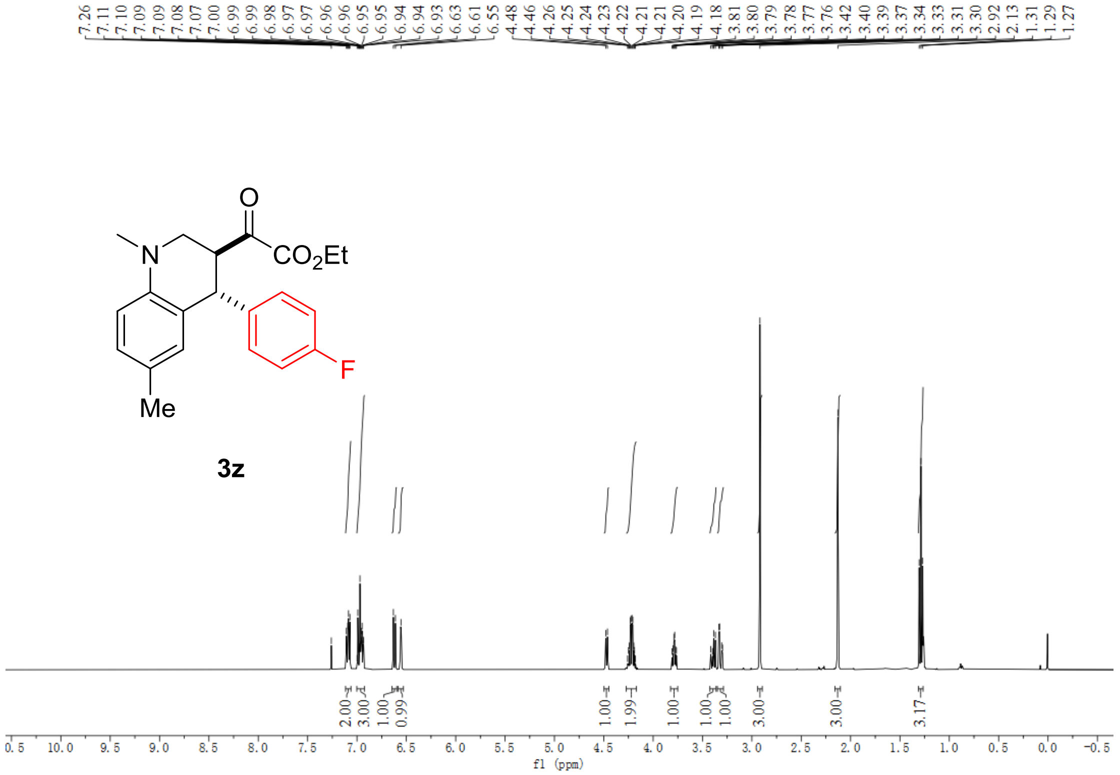

^{13}C-NMR of **3z** (101 MHz, $CDCl_3$)

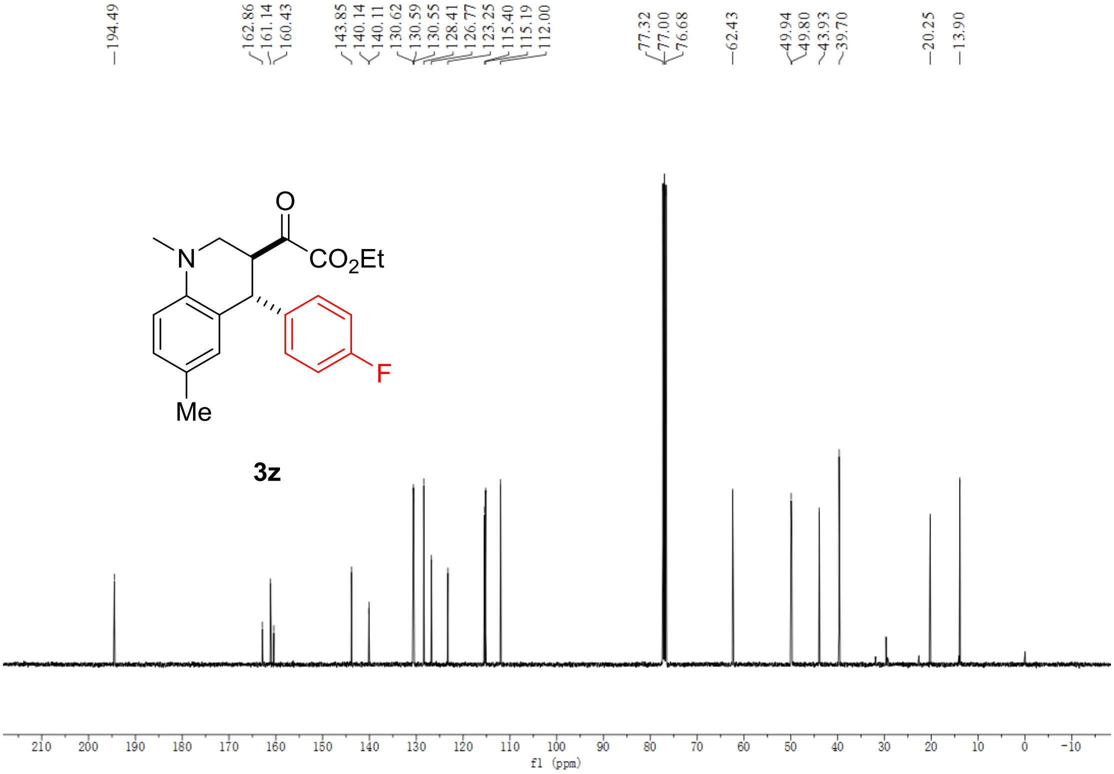

^{19}F-NMR of **3z** (377 MHz, $CDCl_3$)

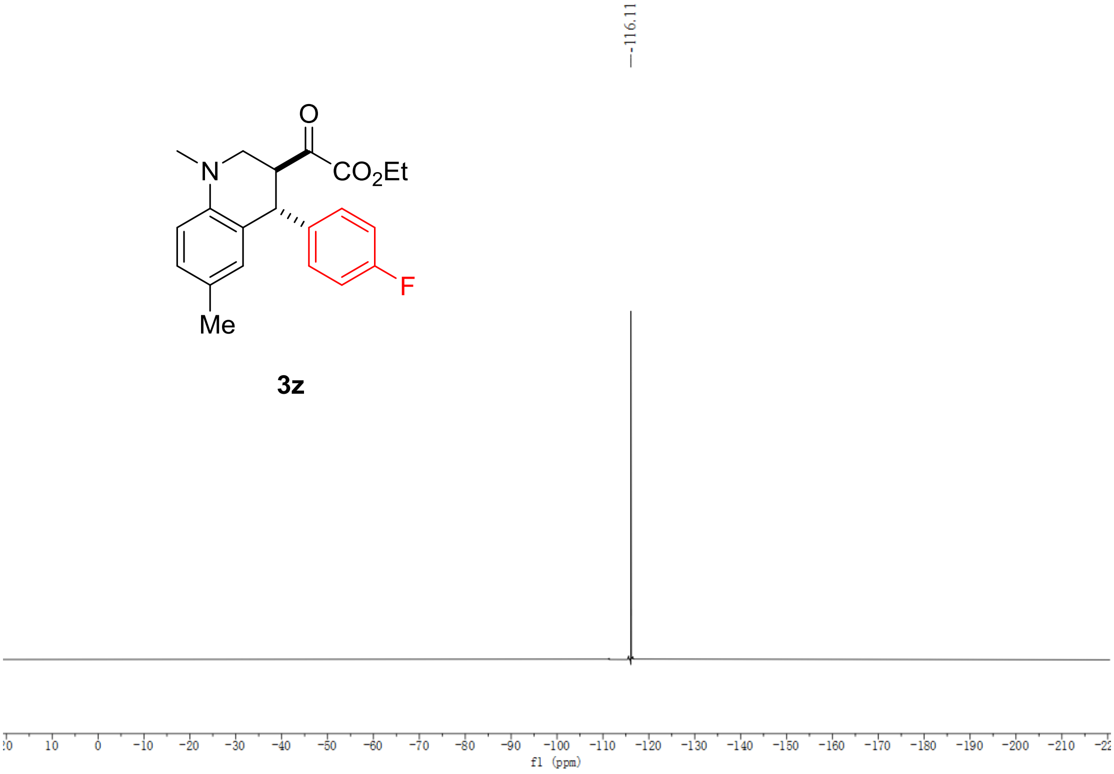

^{1}H-NMR of **3aa** (400 MHz, $CDCl_3$)

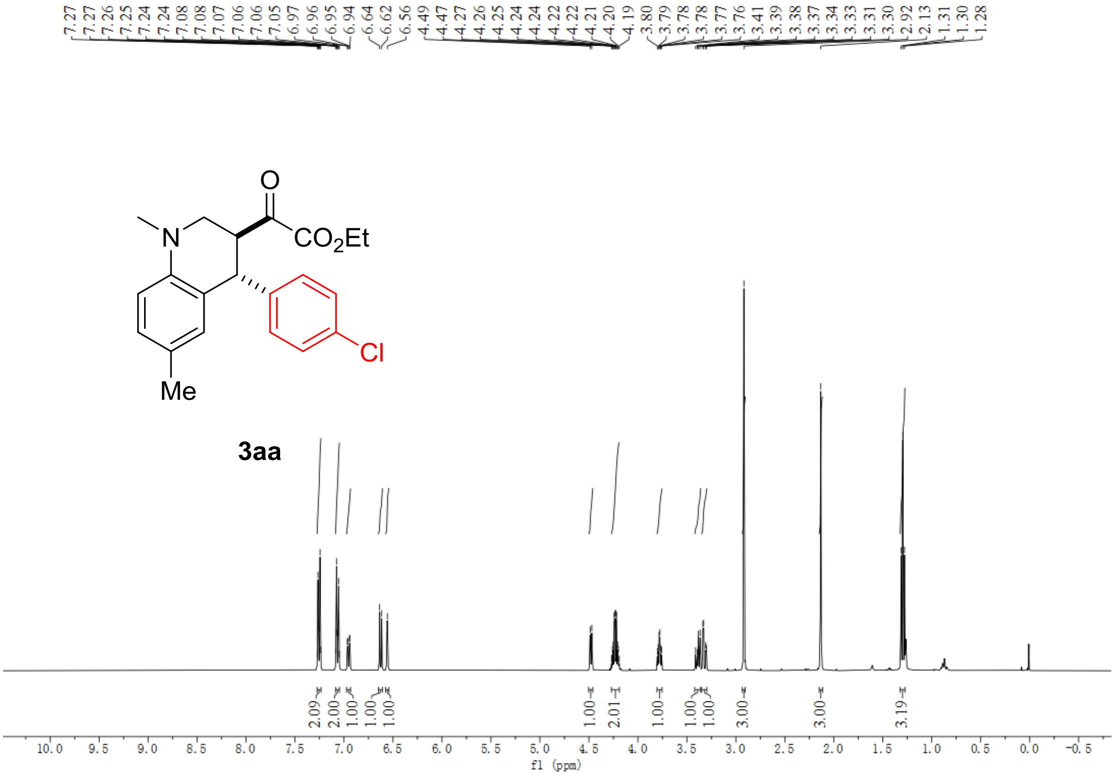

^{13}C-NMR of **3aa** (101 MHz, $CDCl_3$)

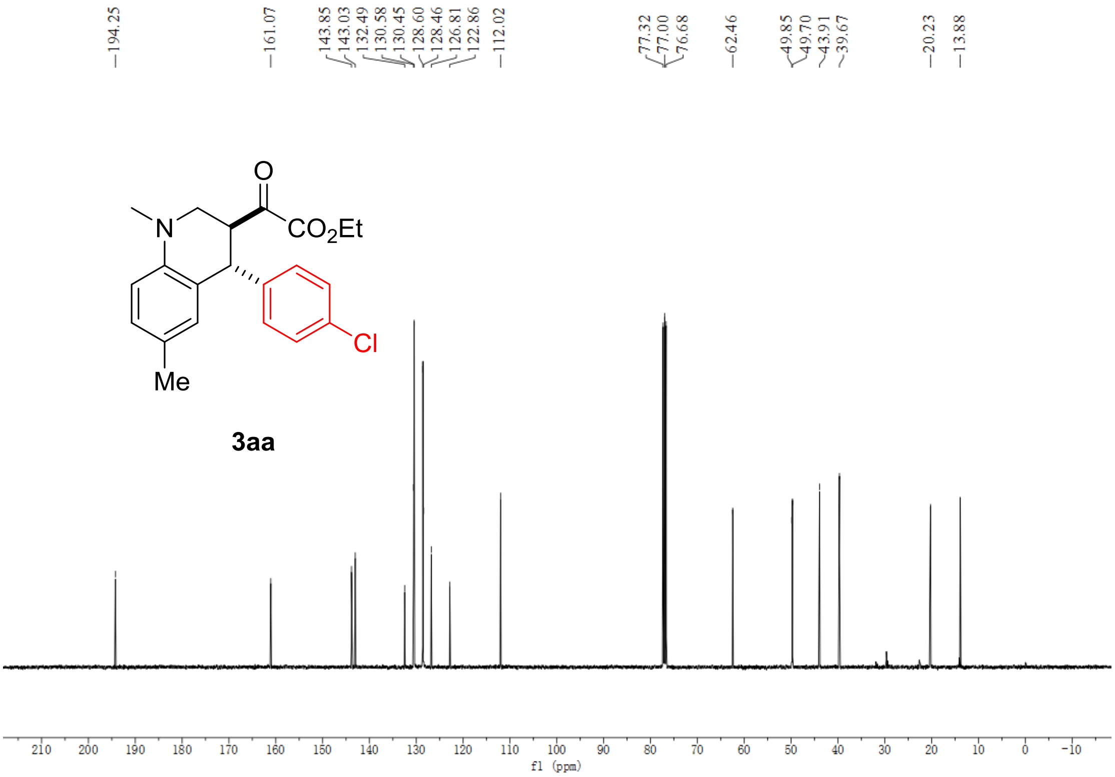

^{1}H-NMR of **3ab** (400 MHz, $CDCl_3$)

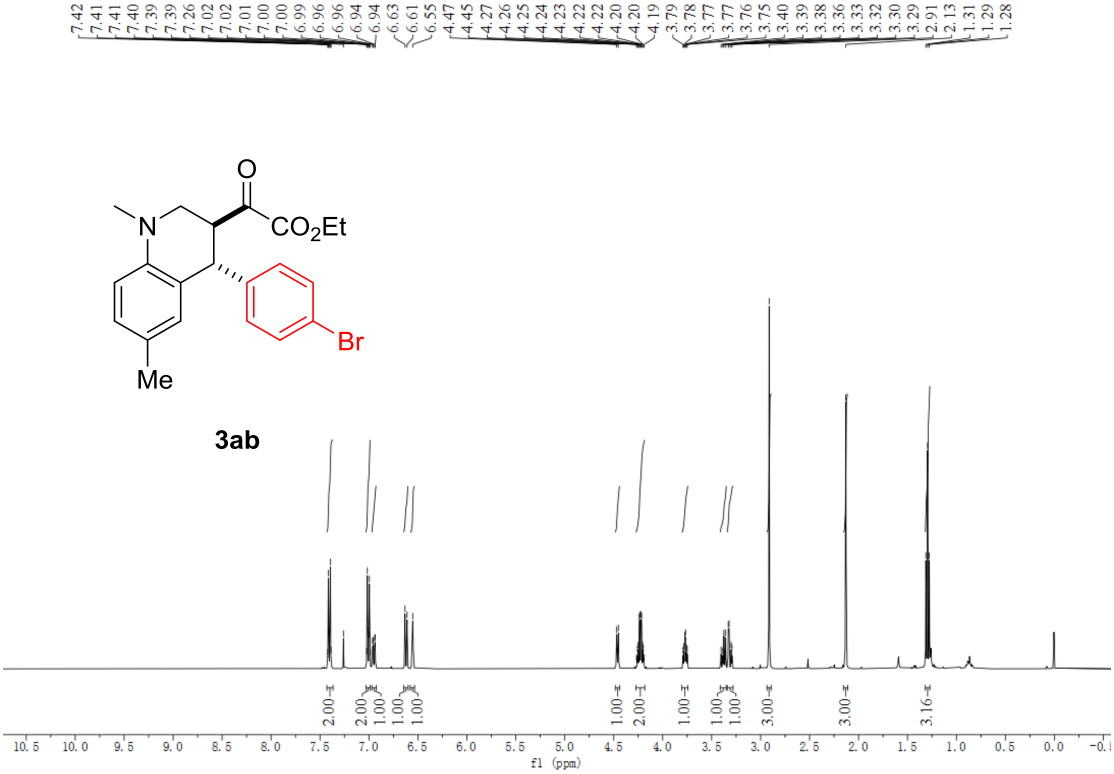

^{13}C-NMR of **3ab** (101 MHz, $CDCl_3$)

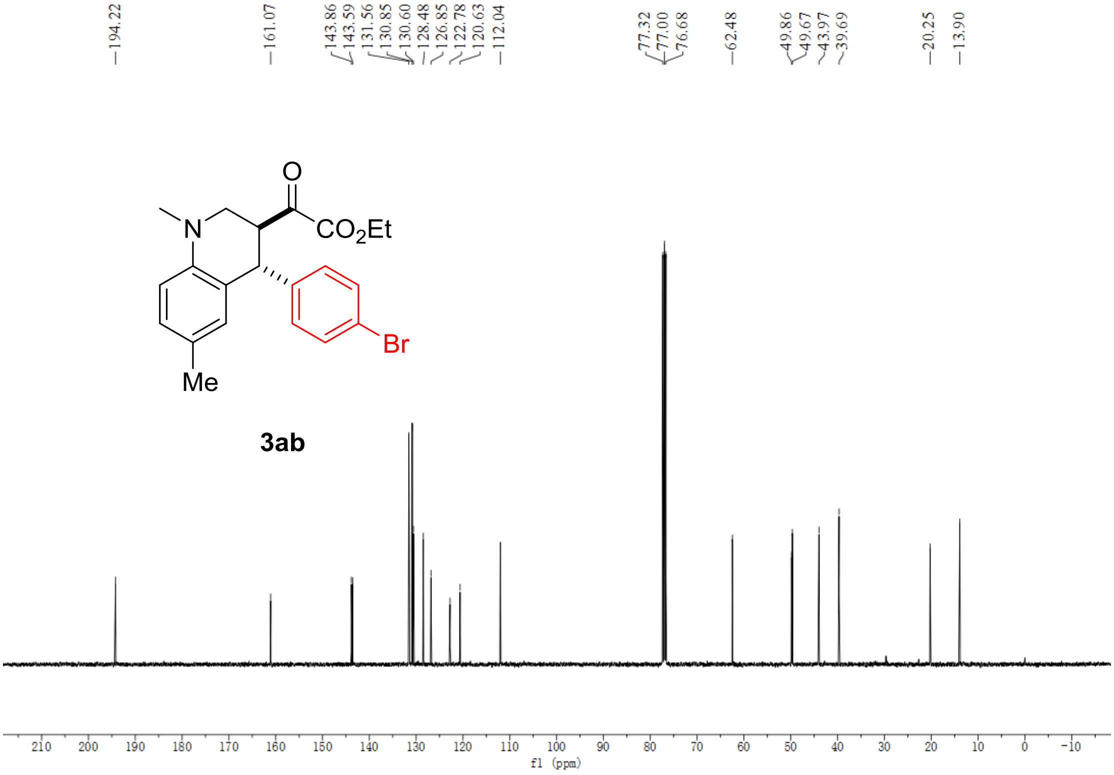

^{1}H-NMR of **3ac** (400 MHz, $CDCl_3$)

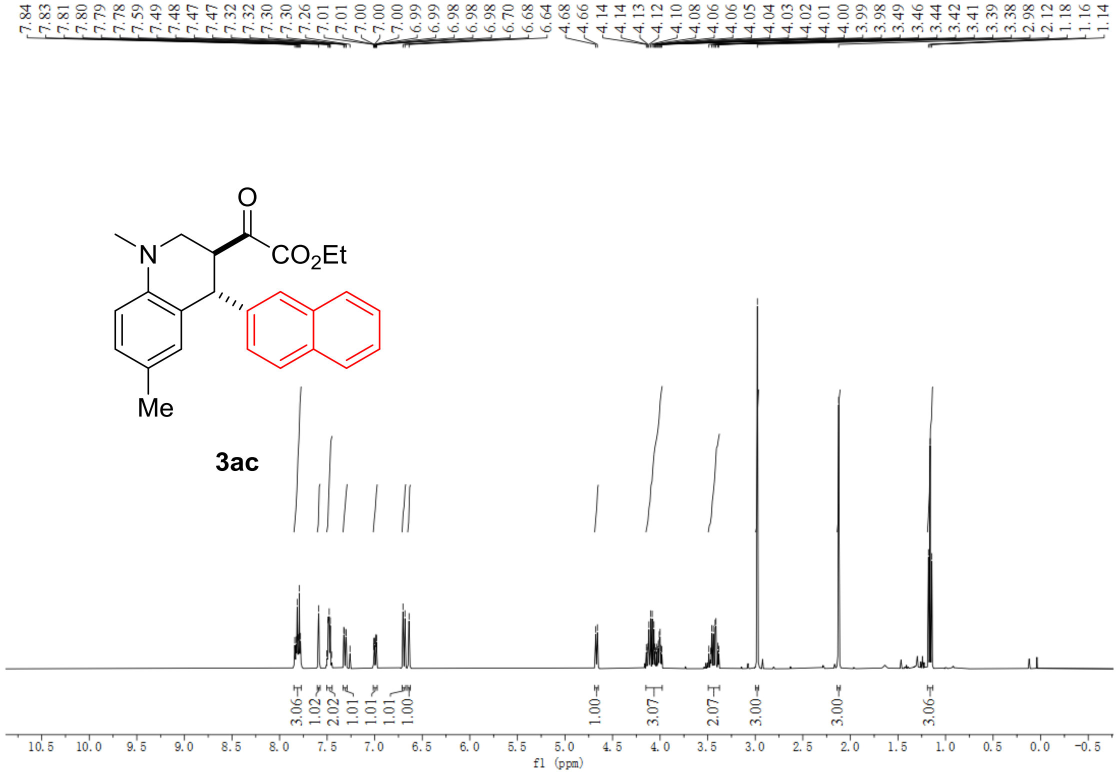

^{13}C-NMR of **3ac** (101 MHz, $CDCl_3$)

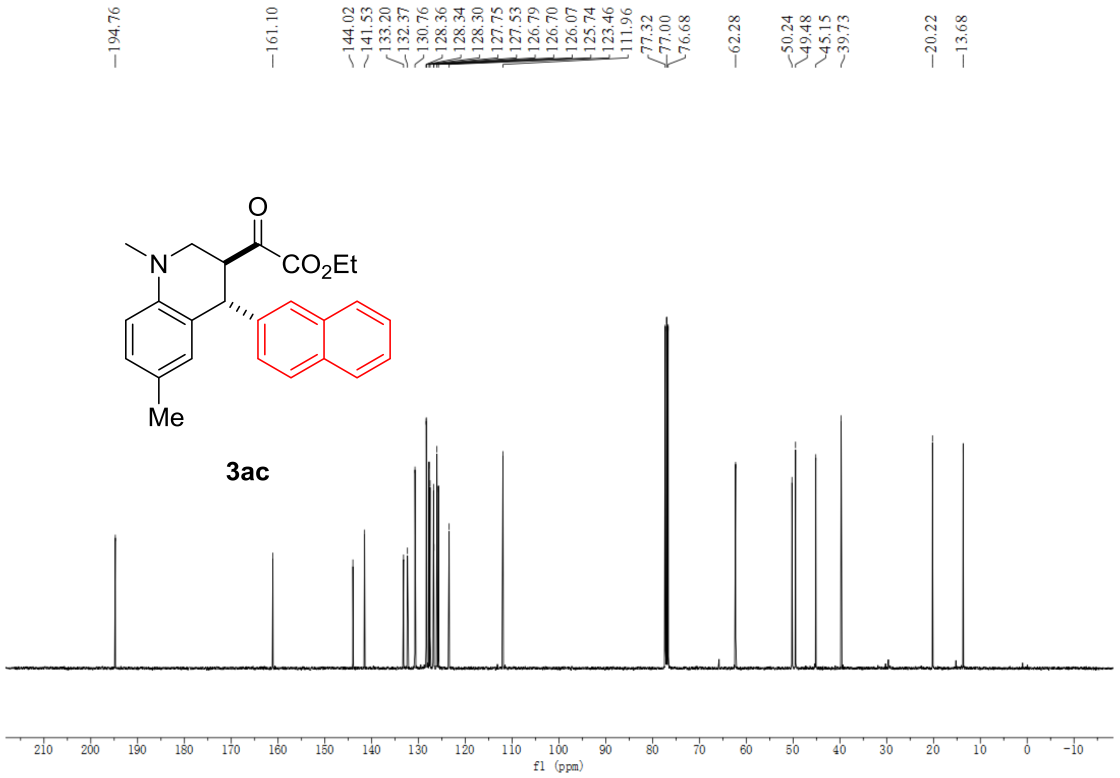

^{1}H-NMR of **3ad** (400 MHz, $CDCl_3$)

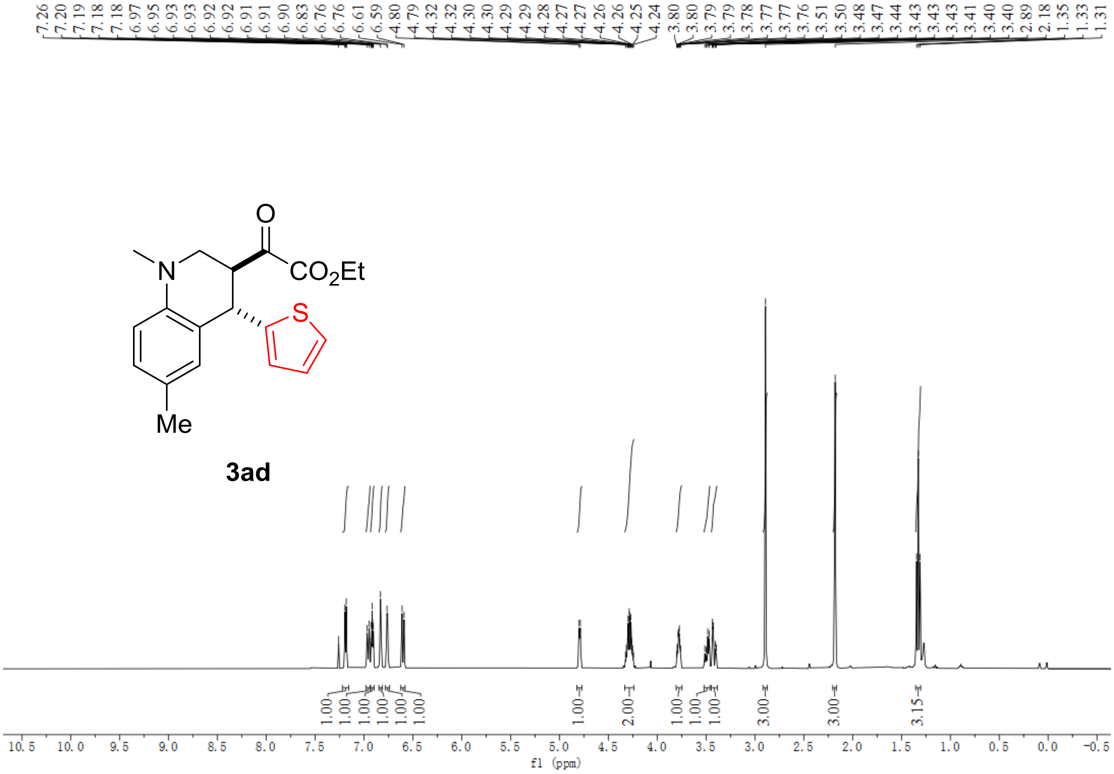

^{13}C-NMR of **3ad** (101 MHz, $CDCl_3$)

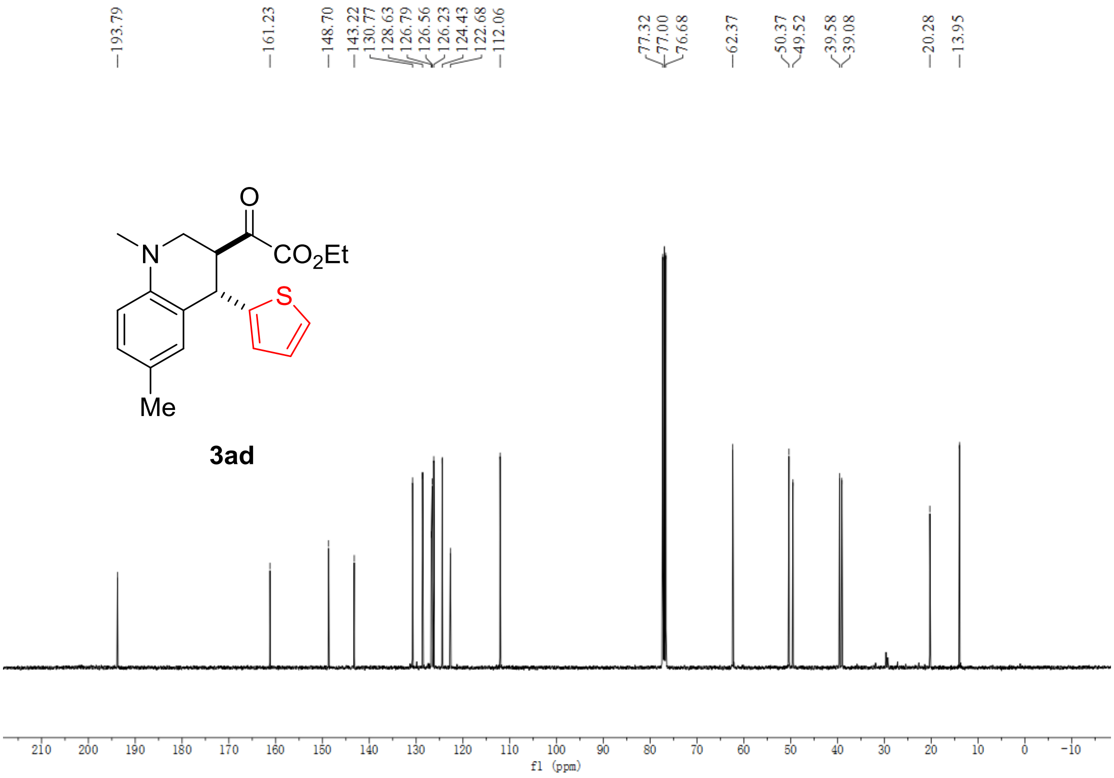

^{1}H-NMR of **3ae** (400 MHz, $CDCl_3$)

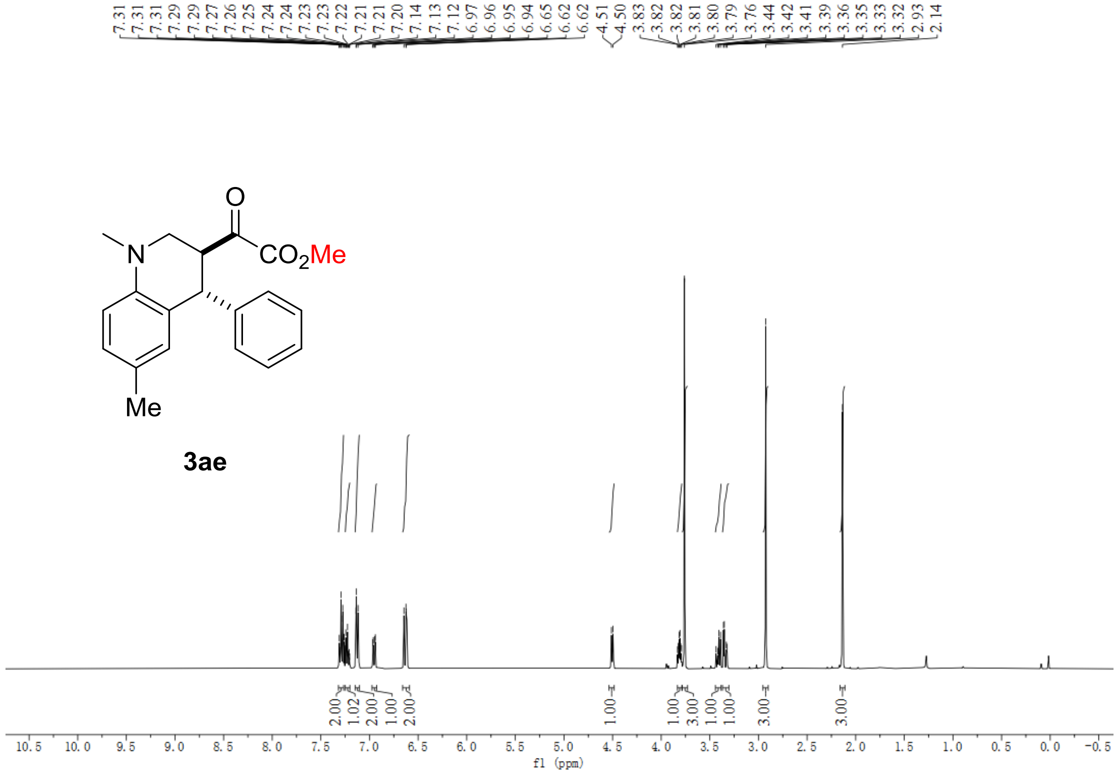

^{13}C-NMR of **3ae** (101 MHz, $CDCl_3$)

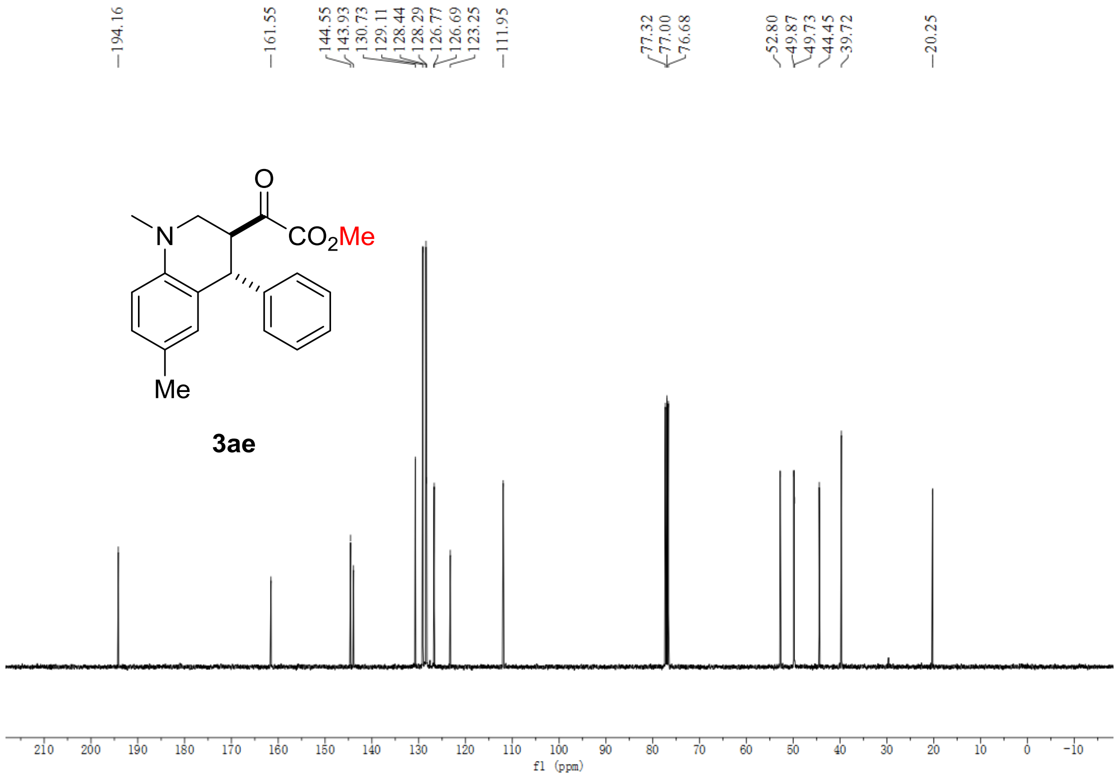

^{1}**H-NMR** of **4a** (400 MHz, $CDCl_3$)

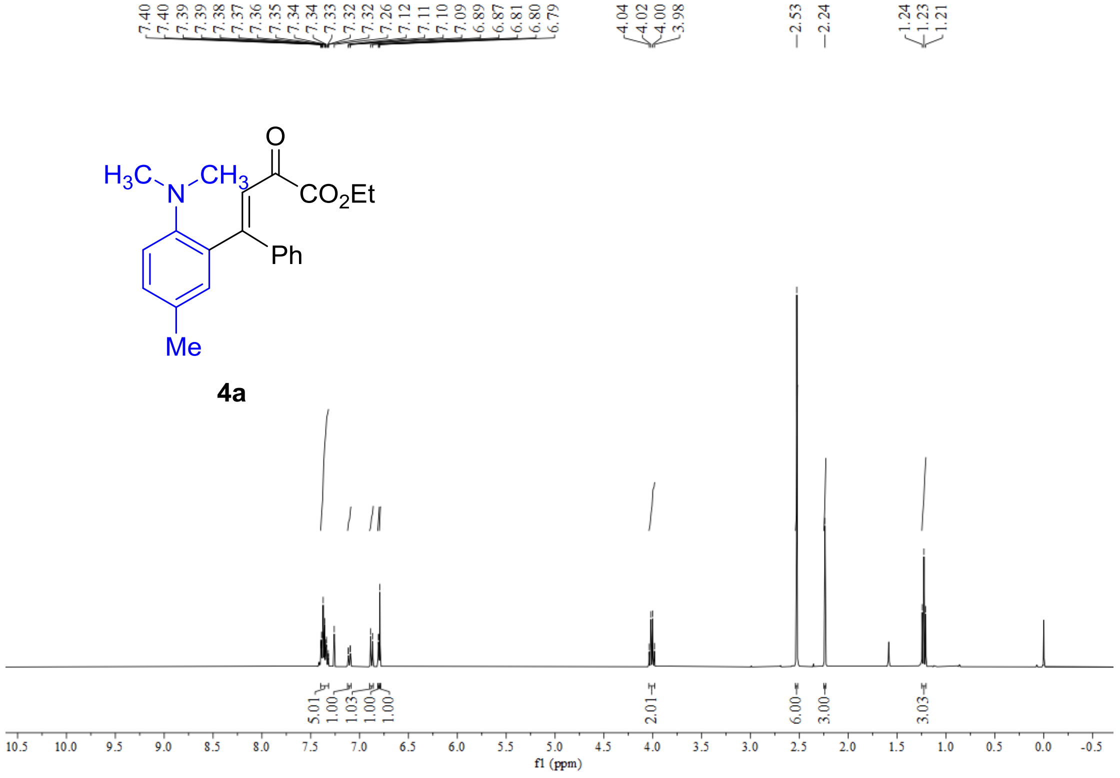

^{13}**C-NMR** of **4a** (101 MHz, $CDCl_3$)

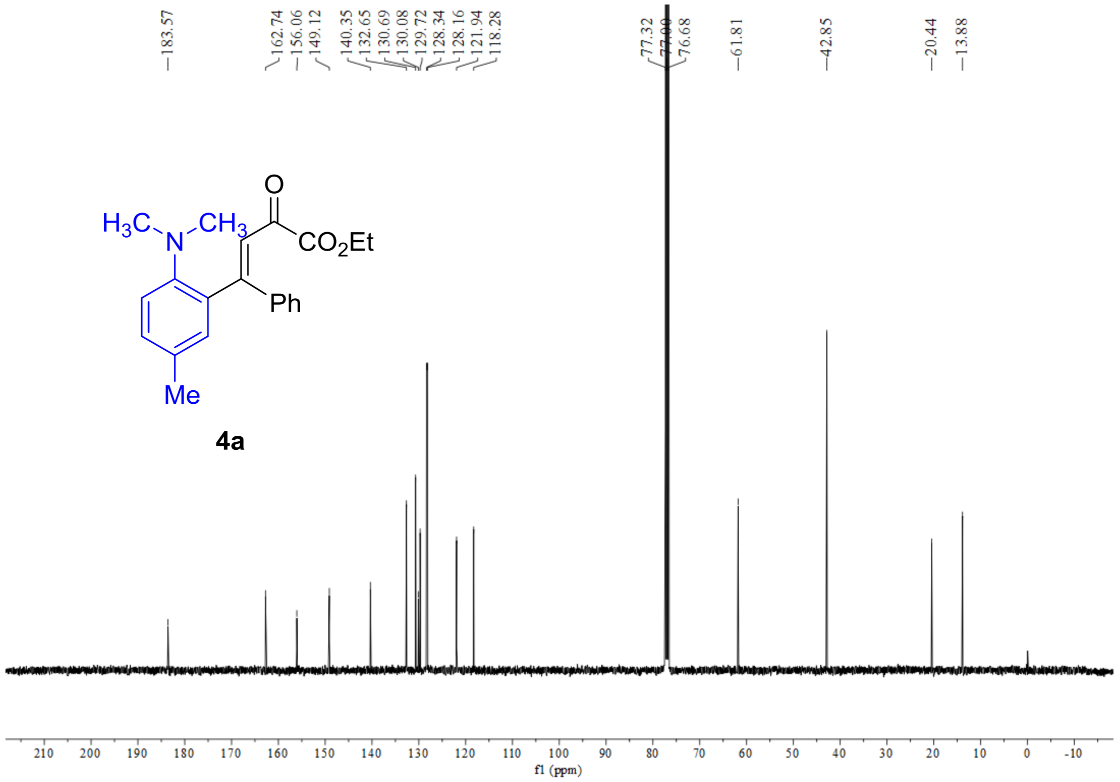

^{1}H-NMR of 6a (400 MHz, $CDCl_3$)

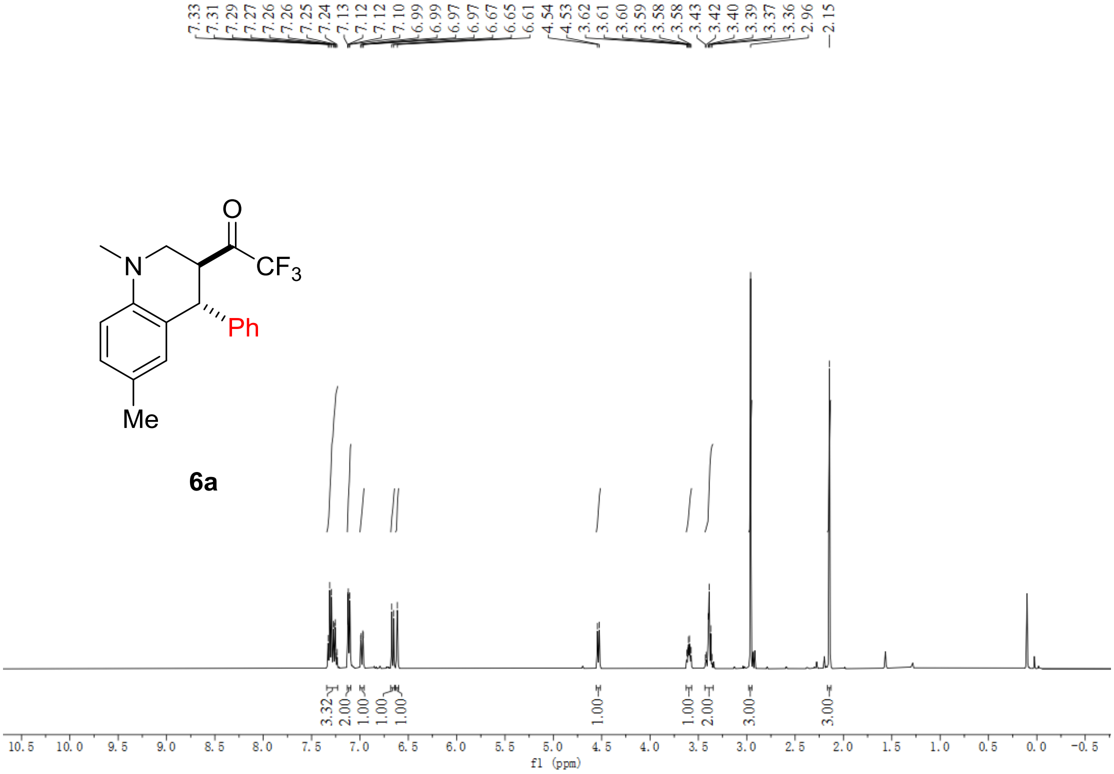

^{13}C-NMR of 6a (101 MHz, $CDCl_3$)

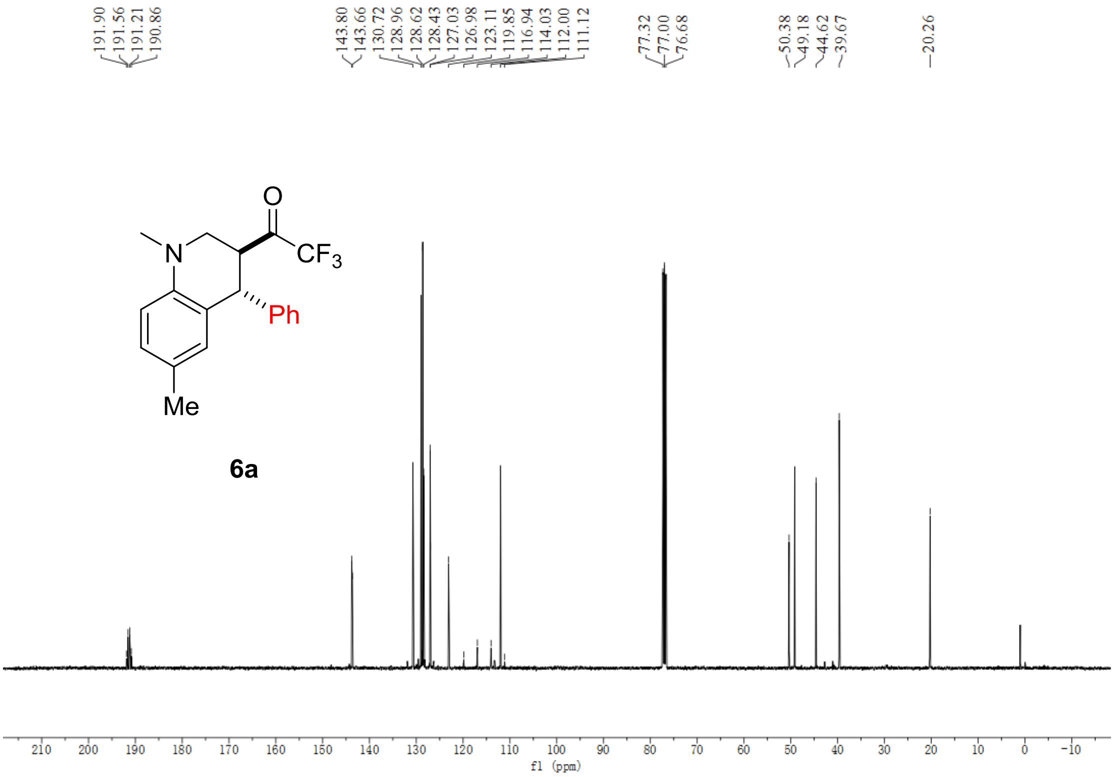

^{19}F-NMR of **6a** (377 MHz, $CDCl_3$)

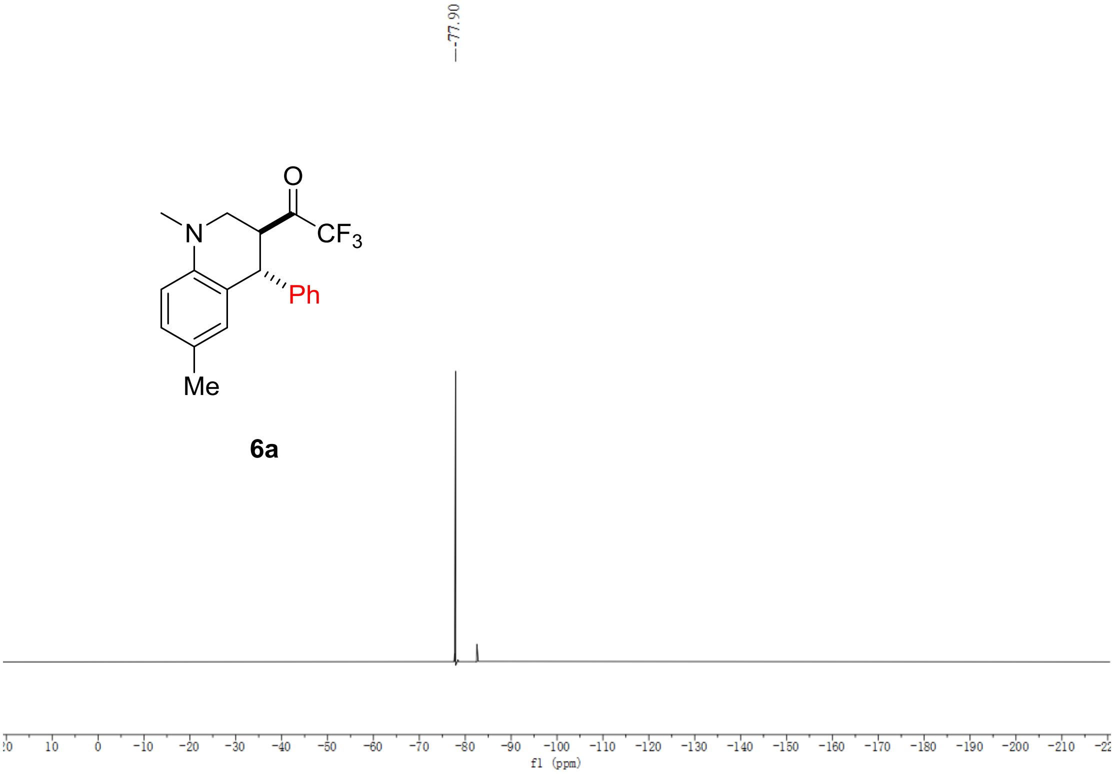

^{1}H-NMR of **6b** (400 MHz, $CDCl_3$)

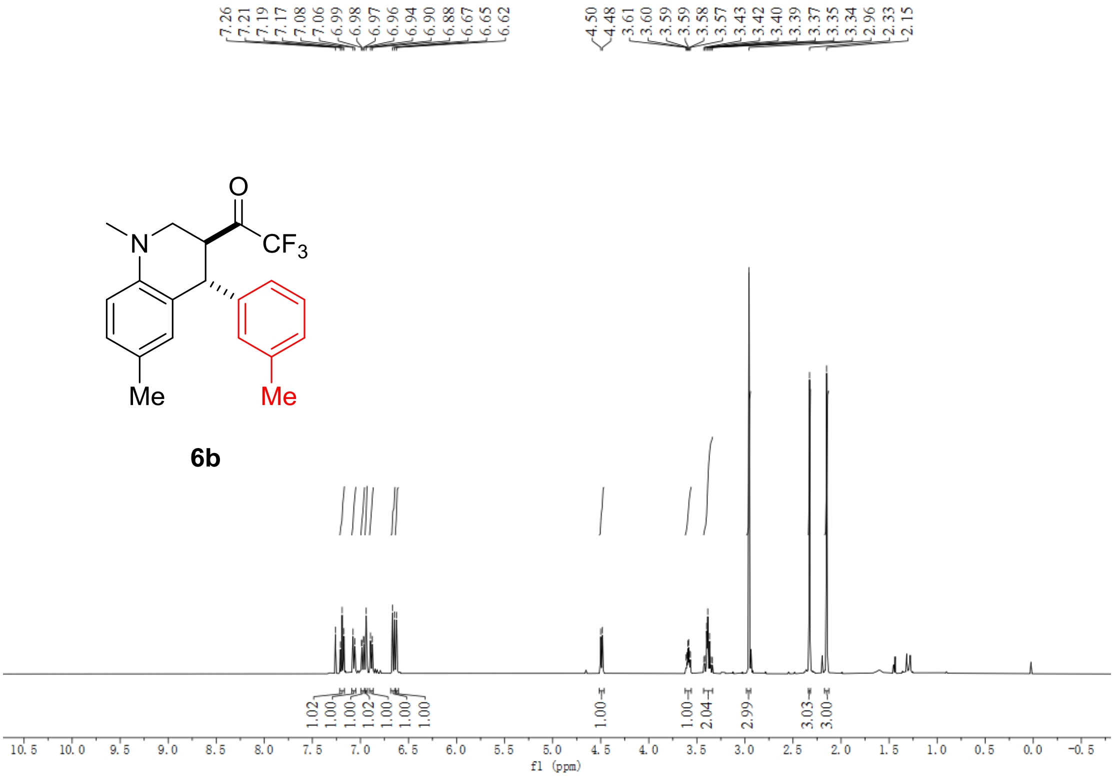

^{13}C-NMR of 6b (101 MHz, $CDCl_3$)

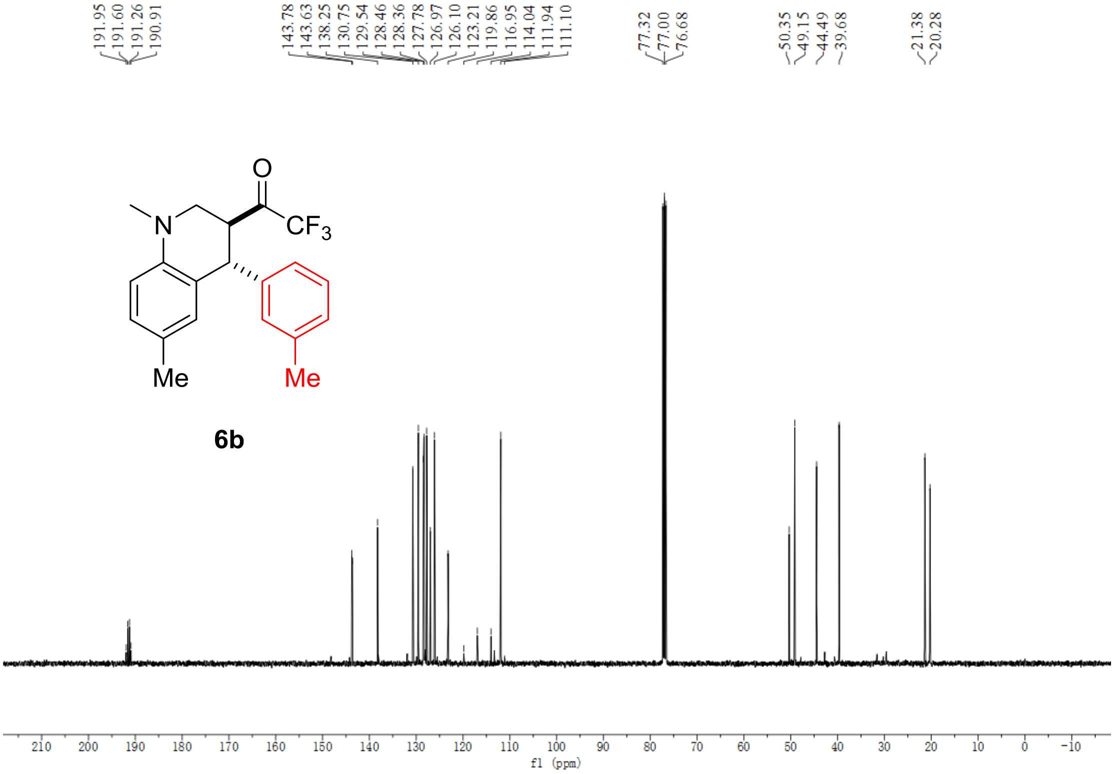

^{19}F-NMR of 6b (377 MHz, $CDCl_3$)

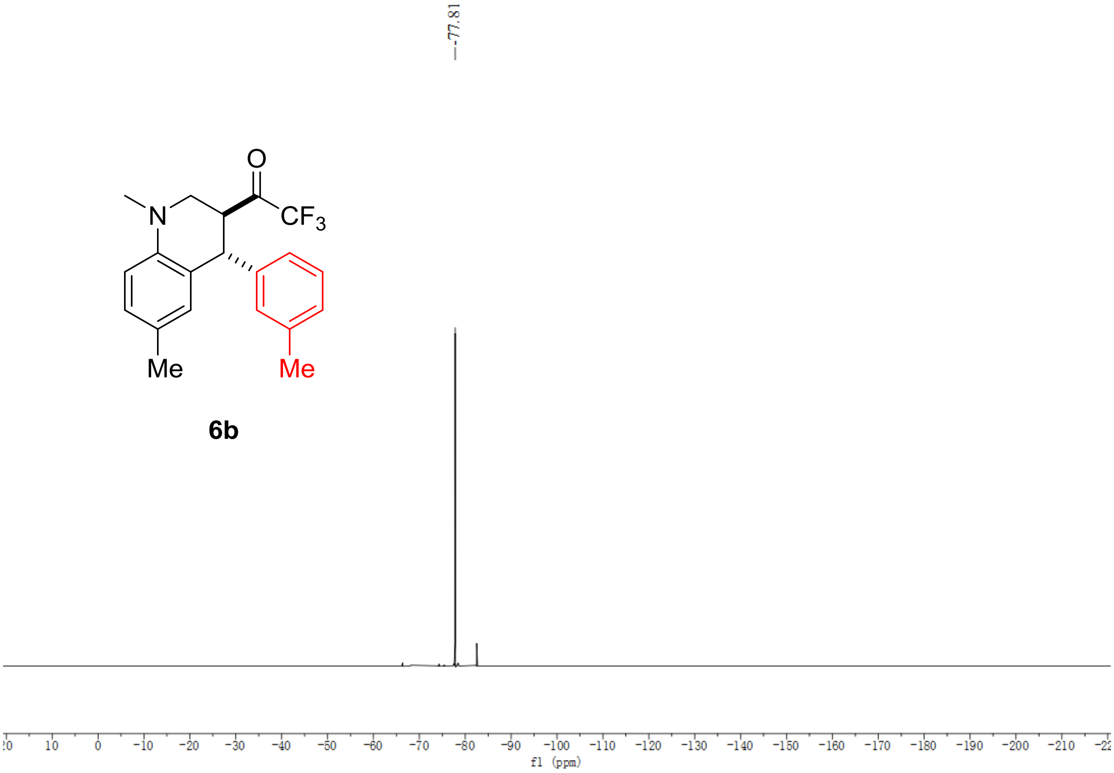

^{1}H-NMR of **6c** (400 MHz, $CDCl_3$)

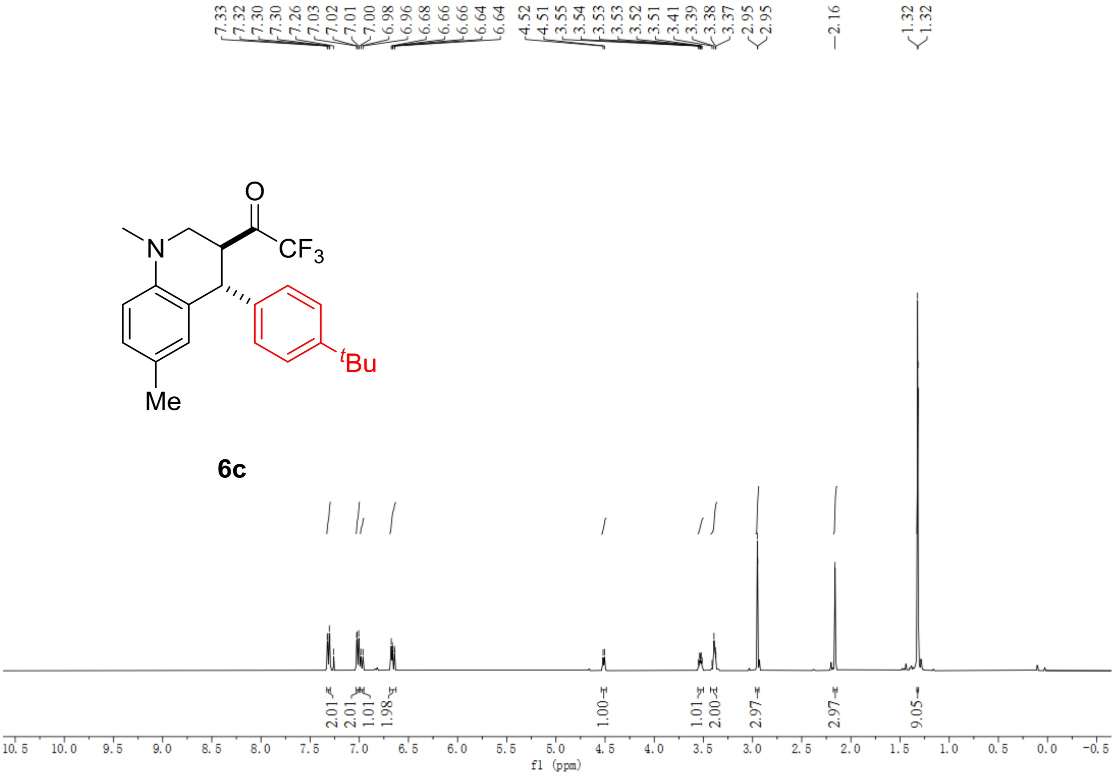

^{13}C-NMR of **6c** (101 MHz, $CDCl_3$)

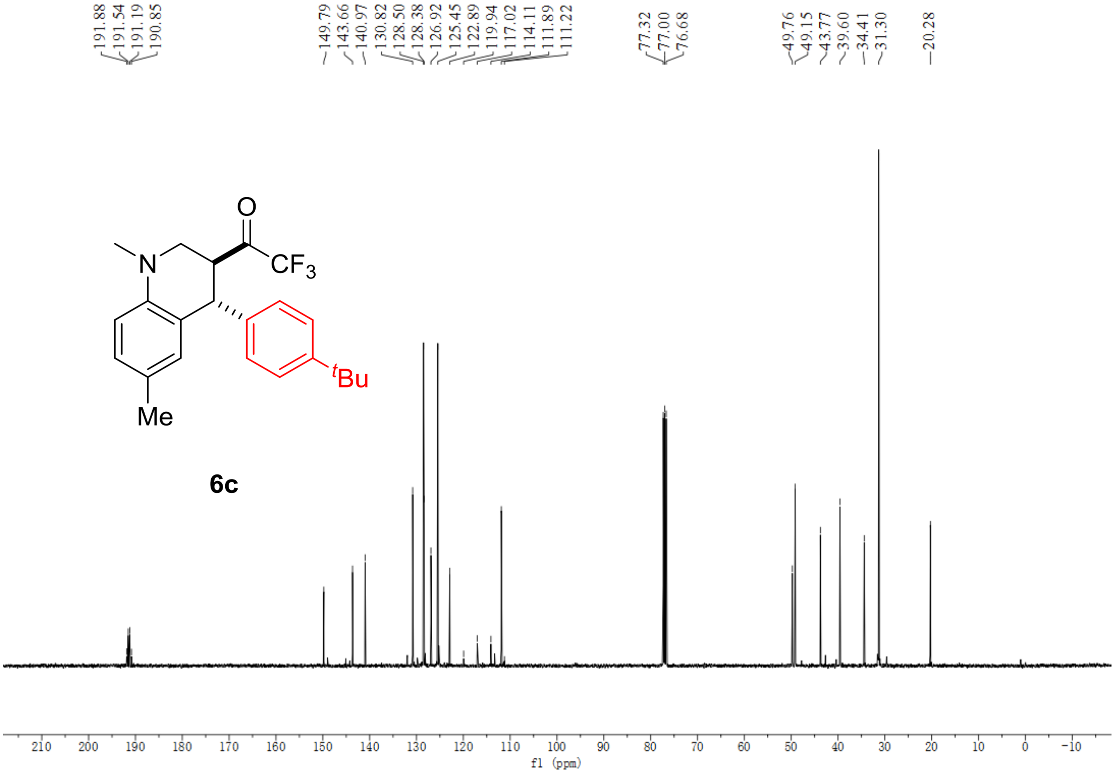

^{19}F-NMR of **6c** (377 MHz, $CDCl_3$)

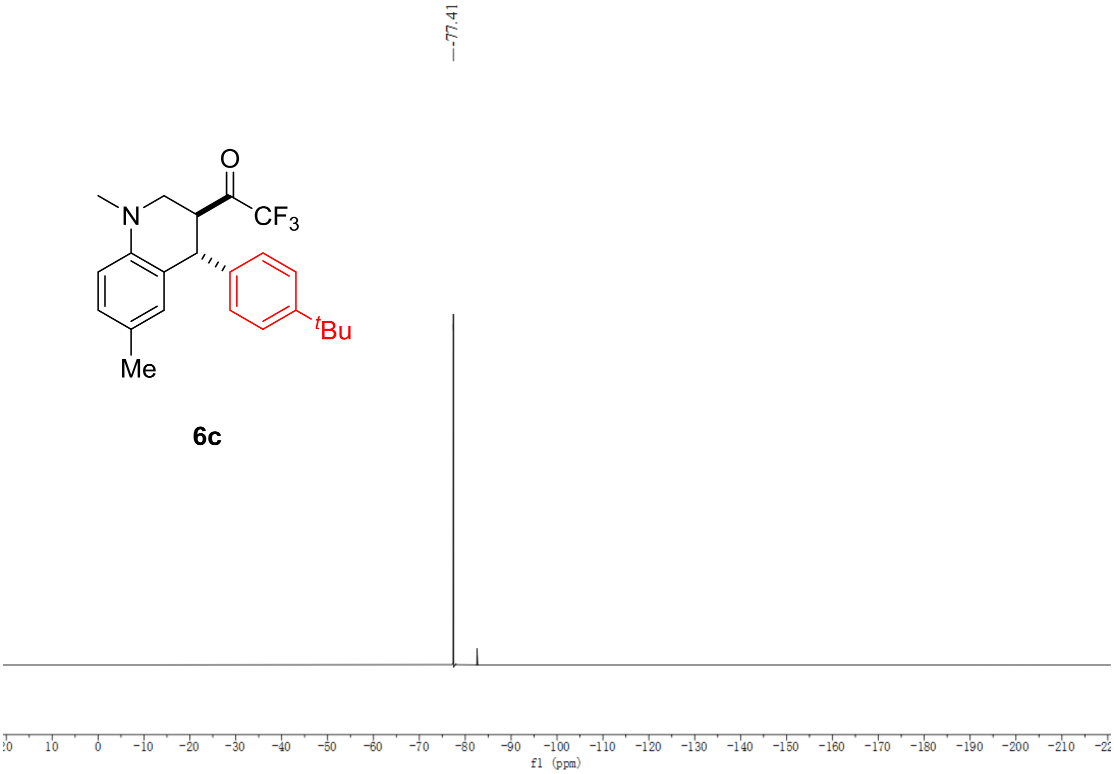

^{1}H-NMR of **6d** (400 MHz, $CDCl_3$)

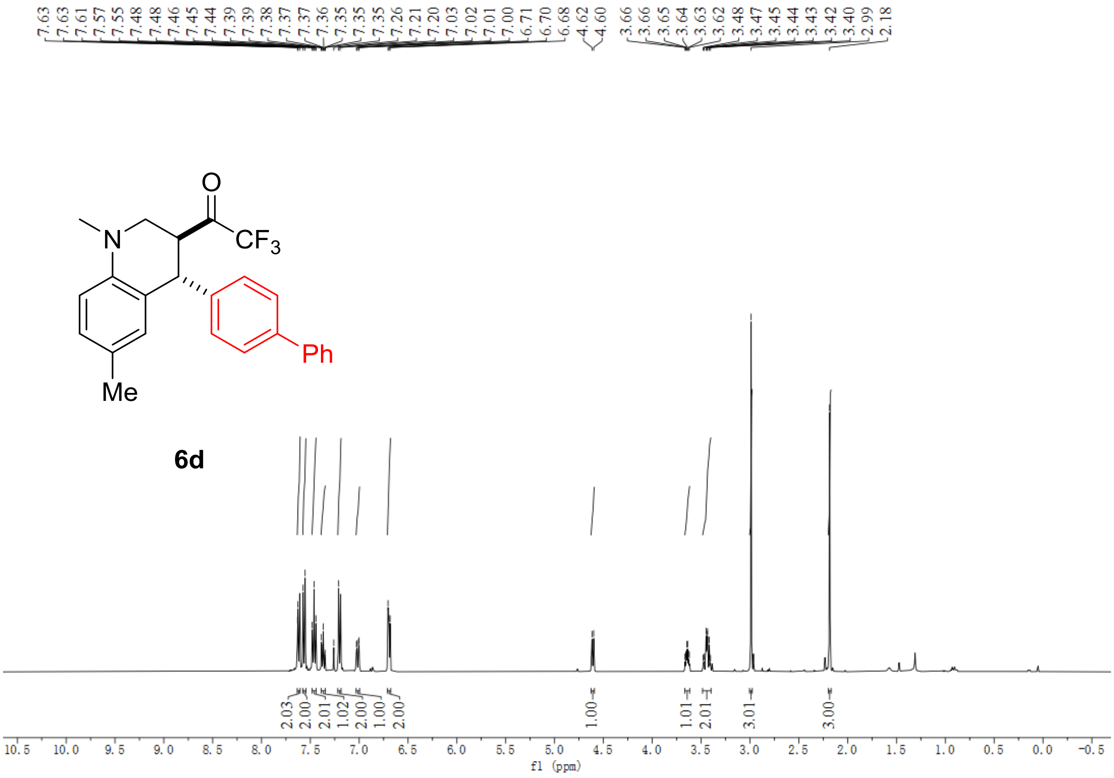

^{13}C-NMR of **6d** (101 MHz, $CDCl_3$)

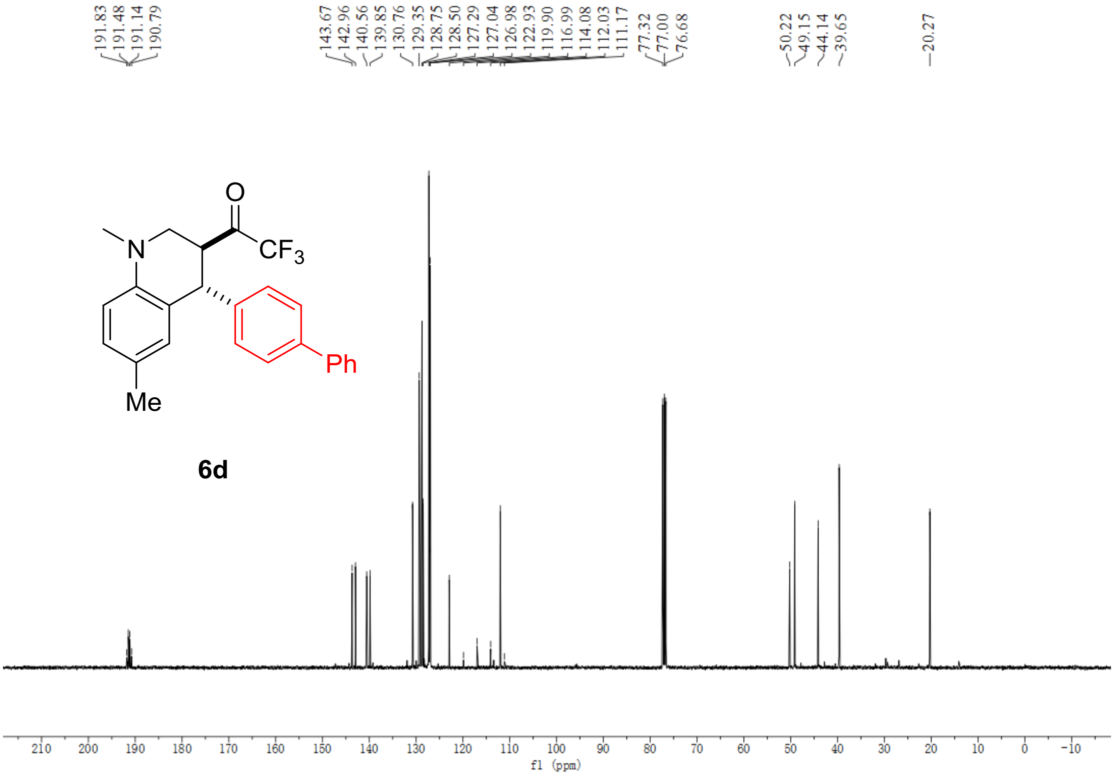

^{19}F-NMR of **6d** (377 MHz, $CDCl_3$)

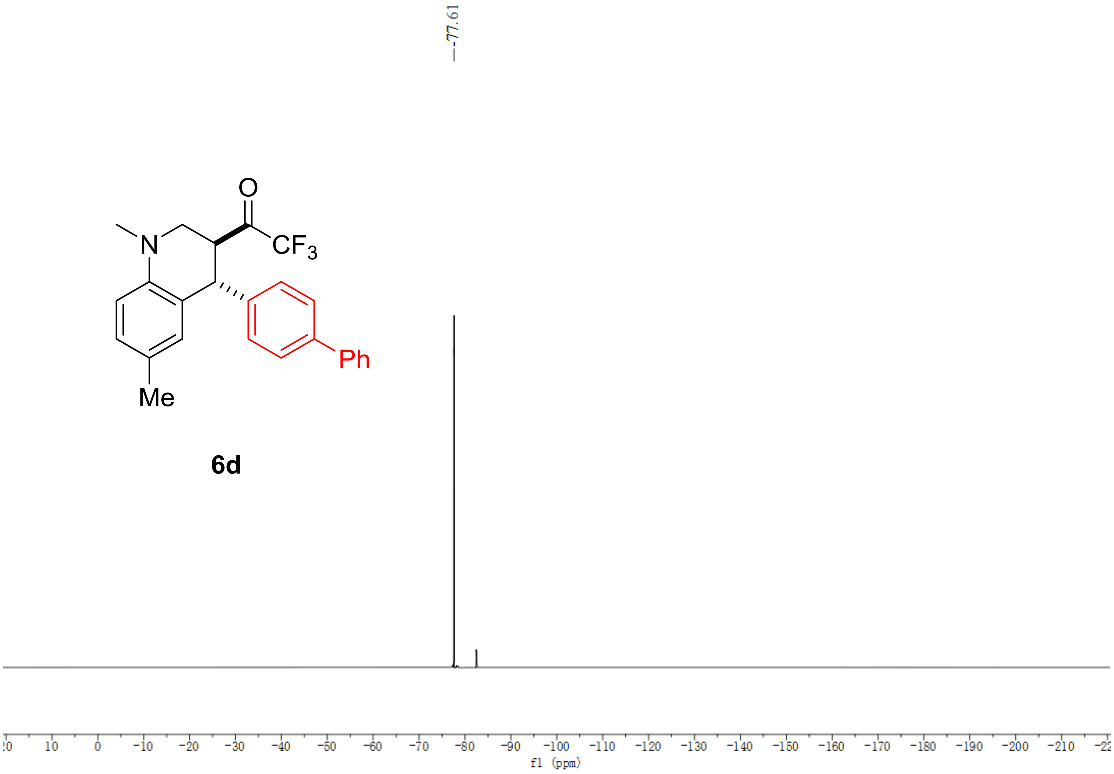

^{1}H-NMR of **6e** (400 MHz, $CDCl_3$)

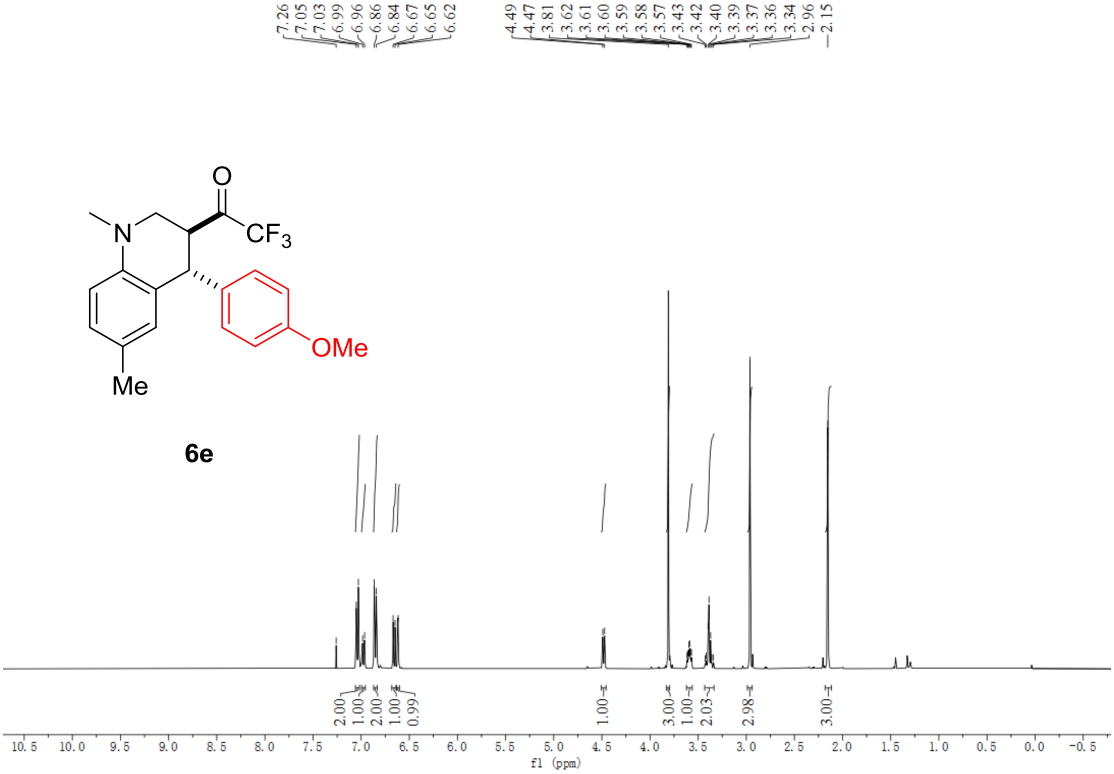

^{13}C-NMR of **6e** (101 MHz, $CDCl_3$)

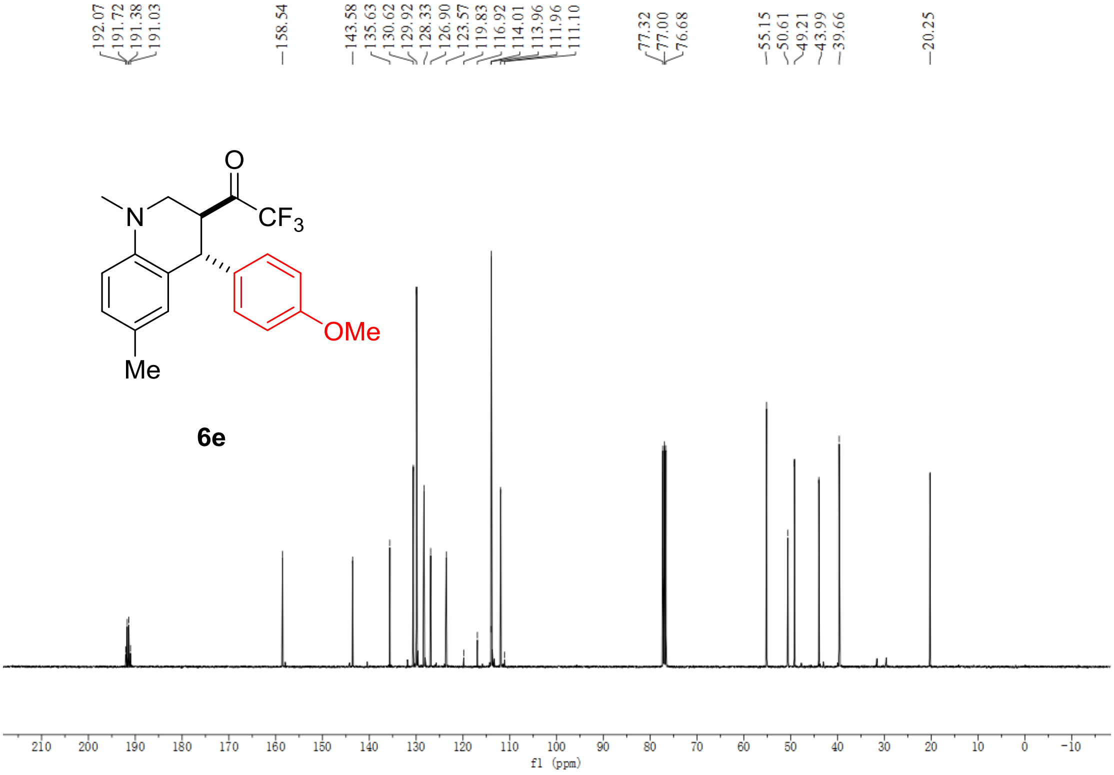

^{19}F-NMR of **6e** (377 MHz, $CDCl_3$)

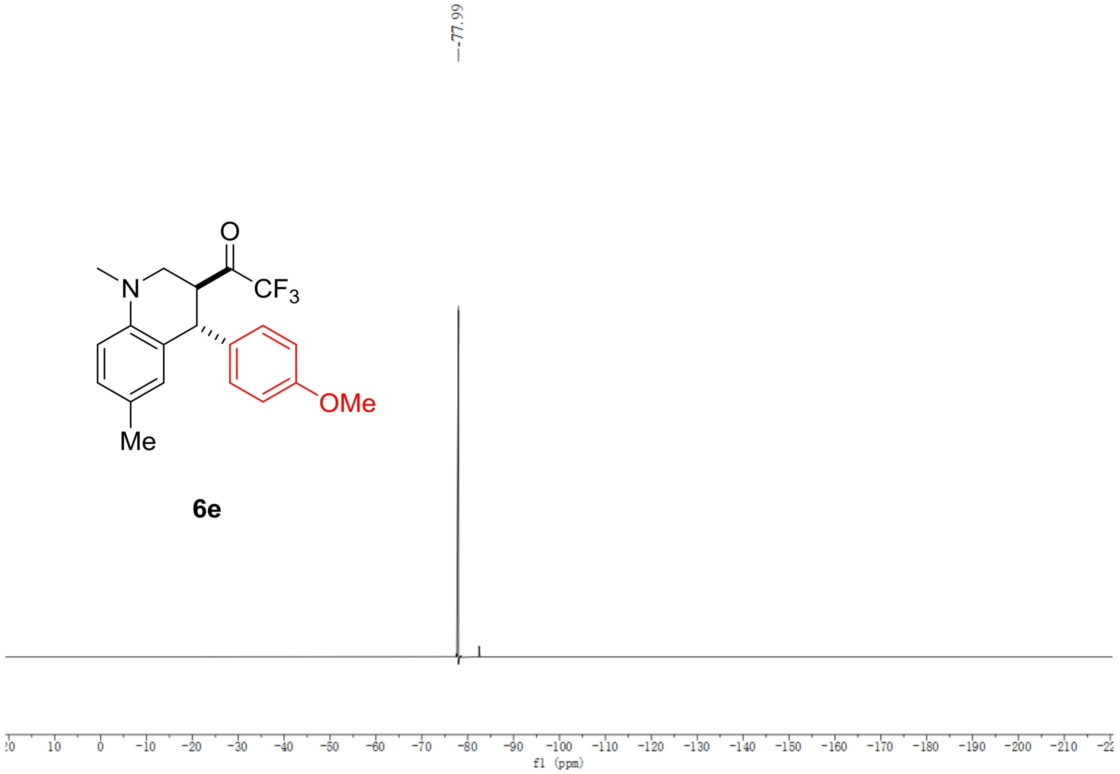

^{1}H-NMR of **6f** (400 MHz, $CDCl_3$)

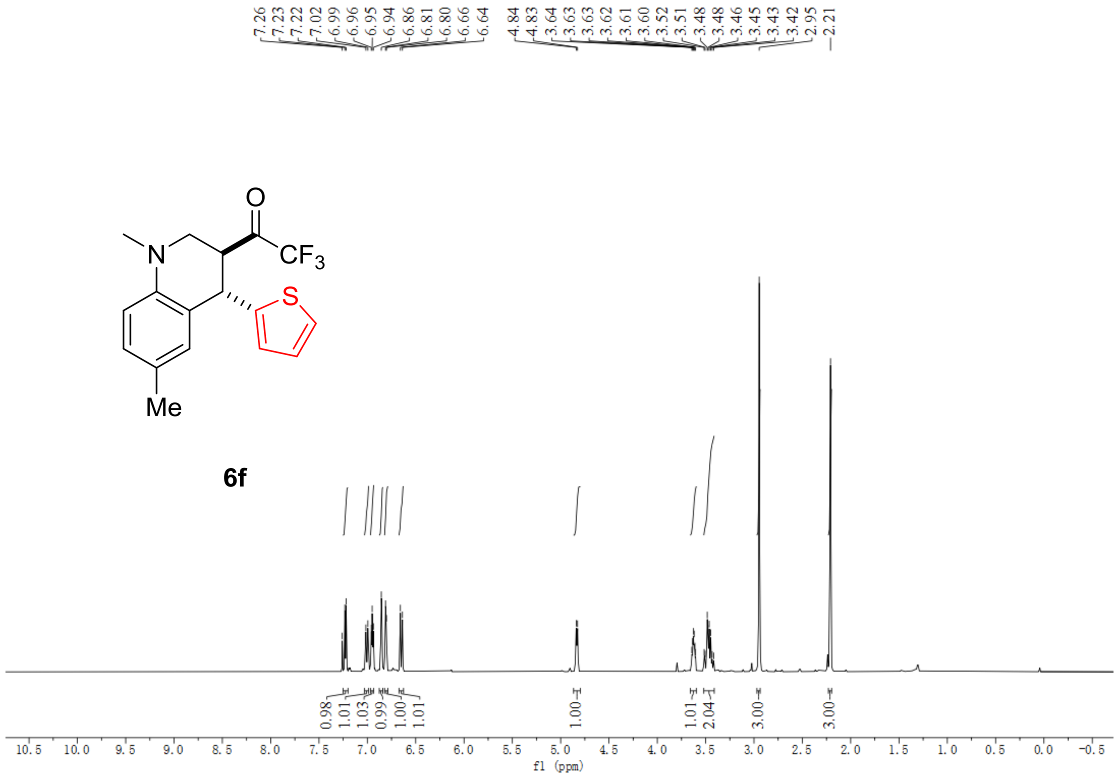

^{13}C-NMR of 6f (101 MHz, $CDCl_3$)

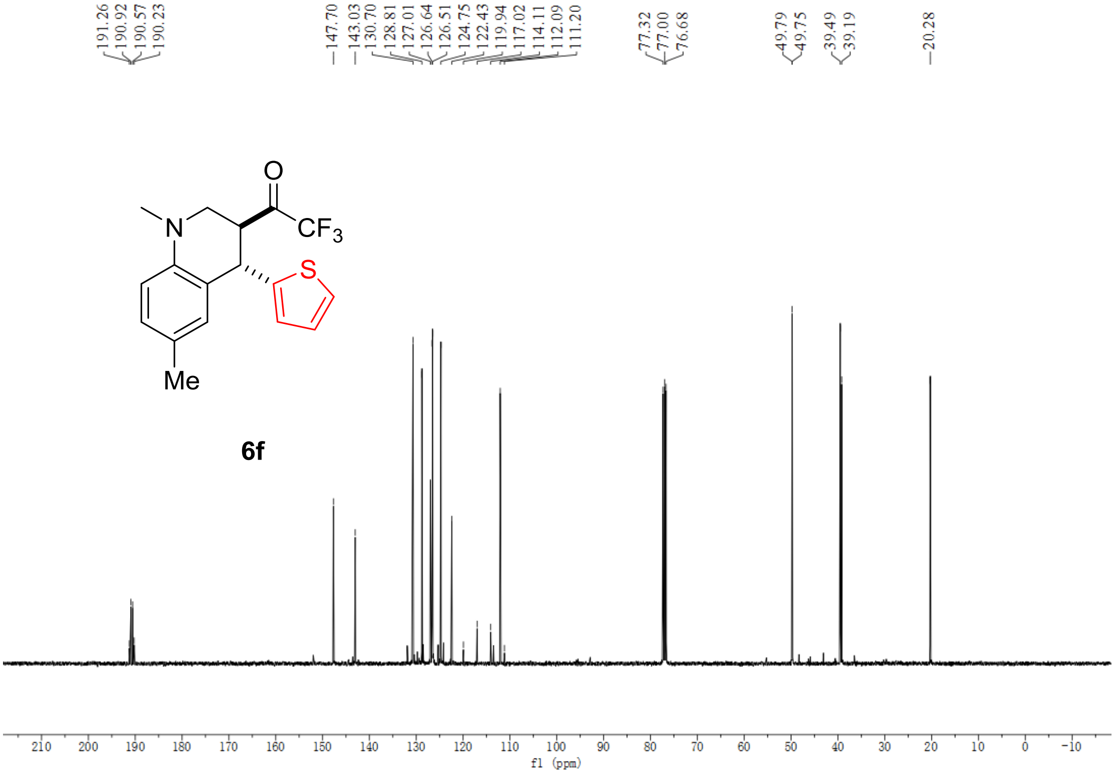

^{19}F-NMR of 6f (377 MHz, $CDCl_3$)

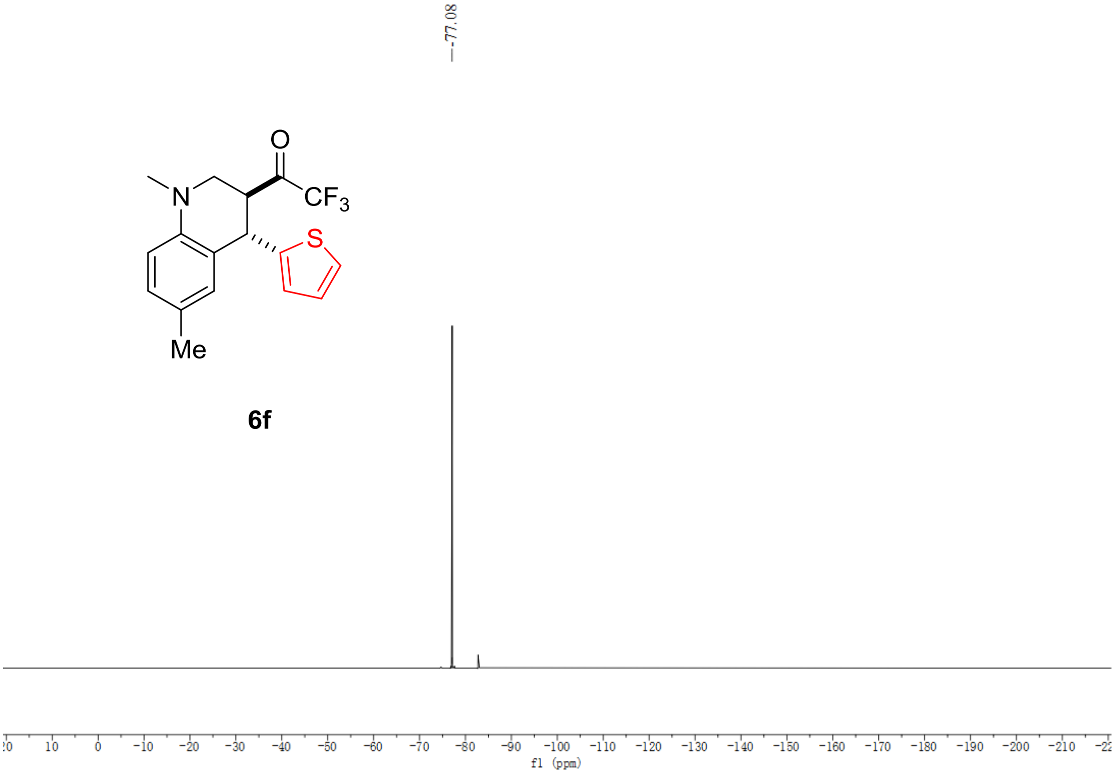

Supplement: SC-013-D1SC05629H-s001 [file SC-013-D1SC05629H-s001.pdf]
